# Ni-Catalyzed [2 + 2 + 2] Cycloaddition via the Capture of Azametallacyclopentadienes with Allyl Boronate: Facile Access to Fused Pyridine Derivatives

**DOI:** 10.3390/molecules30173629

**Published:** 2025-09-05

**Authors:** Kesi Du, Tao Zhu, Guangyu Li, Taohong Shi, Chunsheng Li, Siting Hu, Ruiran Gao, Zhao-Yang Wang, Jiuzhong Huang

**Affiliations:** 1 Guizhou Provincial Engineering Technology Research Center for Chemical Drug R&D, School of Pharmacy, Guizhou Medical University, Guiyang 550004, China; 2 Jiangxi Province Key Laboratory of Pharmacology of Traditional Chinese Medicine, School of Pharmacy, Gannan Medical University, Ganzhou 341000, China; 3 School of Environmental and Chemical Engineering, Zhaoqing University, Zhaoqing 526061, China; 4 School of Chemistry, South China Normal University, Guangzhou 510006, China

**Keywords:** [2 + 2 + 2] cycloaddition, nickel catalysis, azanickelcyclopentadiene, allyl boronate, fused pyridine derivatives

## Abstract

An unprecedented nickel-catalyzed [2 + 2 + 2] cycloaddition that enables efficient construction of fused pyridine frameworks with allyl boronate was reported. This transformation is proposed to occur through a mechanism involving aza-nickelacyclopentadiene intermediates, wherein the boryl group of the allyl boronate plays a critical role in enabling the following cyclization via the control experiments. This work not only expands the structural diversity accessible via transition-metal-catalyzed [2 + 2 + 2] cycloadditions but also showcases the untapped potential of unsaturated substrates in cycloaddition reactions.

## 1. Introduction

**Figure 1:**
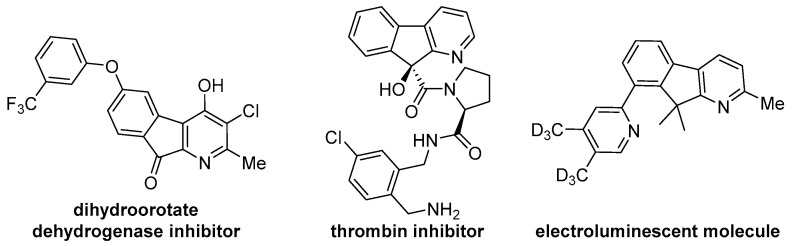
Pyridine-based functional molecules.

Fused pyridine skeleton represents a privileged scaffold prevalent in numerous heterocyclic compounds and natural products (Figure 1), serving as fundamental structural units in pharmaceuticals, functional materials, and as versatile ligands or catalysts [1,2,3,4,5,6,7]. Although significant progress has been made in developing synthetic strategies for pyridine derivatives [8,9,10,11,12,13], the catalytic construction of densely substituted pyridines remains a formidable challenge in synthetic chemistry.

De novo synthetic approaches offer distinct advantages for assembling pyridyl cores with diverse substitution patterns from readily accessible building blocks. In this context, transition-metal-catalyzed [2 + 2 + 2] cycloaddition of two alkyne units with a nitrile component has emerged as an atom-economical and versatile strategy for synthesizing six-membered cyclic and aromatic compounds [14,15,16].

**Scheme 1:**
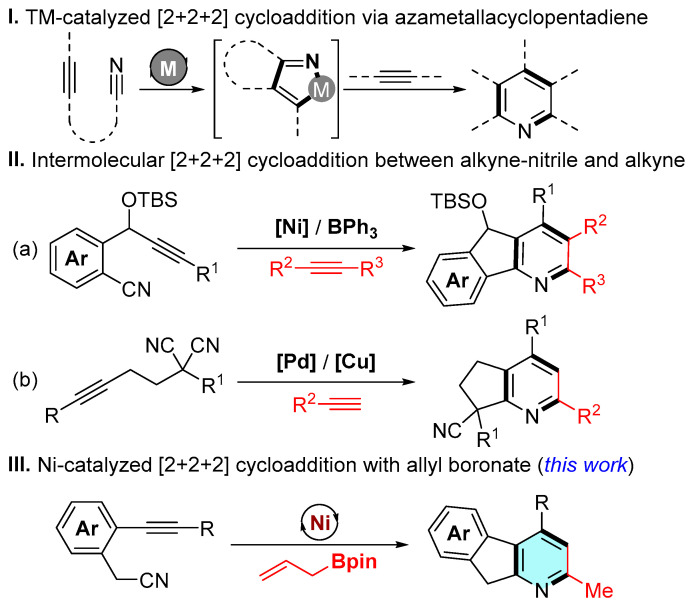
Background and project synopsis.

A key intermediate in these transformations is the azametallacyclopentadiene, featuring one M-C(sp^2^) and one M-N(sp^2^) bond, which forms via metal-mediated oxidative cyclometalation of two triple-bond units [17,18,19]. Typically, azametallacyclopentadienes subsequently undergo alkyne insertion to afford the final cyclic/aromatic products (Scheme 1I) [20,21,22,23]. Considerable efforts have explored diverse catalytic systems based on (aza)metallacyclopentadienes, with successful implementations reported using ruthenium [24], rhodium [25], iridium [26], cobalt [27], iron [28], nickel [29,30], and niobium [31] catalysts [14,15]. For instance, the Liu group reported a Ni/BPh_3_ co-catalyzed [2 + 2 + 2] cycloaddition of alkyne-nitriles with internal alkynes, providing an efficient route to fused pyridines (Scheme 1(IIa)) [32]. Recently, Liu and coauthors developed a palladium/copper dual-catalyzed [2 + 2 + 2] cycloaddition of alkyne-tethered malononitriles and alkynes (Scheme 1(IIb)) [33].

Notably, despite the diverse transformations of azametallacyclopentadienes in pyridine synthesis, alkene derivatives remained insurmountable substrates in this process due to subtle electrical property differences with the triple bond. Motivated by our interest in transition-metal-catalyzed transformations of unsaturated hydrocarbons for heterocycle assembly [34,35,36], we herein report a nickel-catalyzed [2 + 2 + 2] cycloaddition for constructing pyridine scaffolds using allyl boronate as cycloaddition partner (Scheme 1III).

## 2. Results and Discussion

The [2 + 2 + 2] cycloaddition of an alkyne-nitrile substrate with allyl boronate was investigated using a nickel catalytic system, affording **3a** in 80% isolated yield under optimal conditions (Table 1, entry 1). Firstly, control experiments established that the nickel catalyst, diphosphine ligand, and base were all essential for the conversion (entries 2–4). Replacing Ni(Ph_3_P)_2_Cl_2_ with Ni(acac)_2_ as the catalyst significantly decreased reaction efficiency (entry 5), while other ligands, such as DPEphos, dppp, dppf, and Cy_3_P, failed to produce the desired cycloaddition product (entries 6–9). Additionally, performing the transformation with Na_3_PO_4_ as a base, a very low yield is obtained (entry 10). Reactions in other solvents, particularly polar solvents, resulted in a complex mixture (entries 11–12). On the other hand, performing the reaction under air did not improve the outcome (entry 13), and lower reaction temperatures were detrimental to the [2 + 2 + 2] cycloaddition (entry 14). Therefore, the optimal reaction conditions were identified employing Ni(PPh_3_)_2_Cl_2_ as the catalyst, XantPhos as the ligand, and K_3_PO_4_ as the base in trifluorotoluene.

**Scheme 2:**
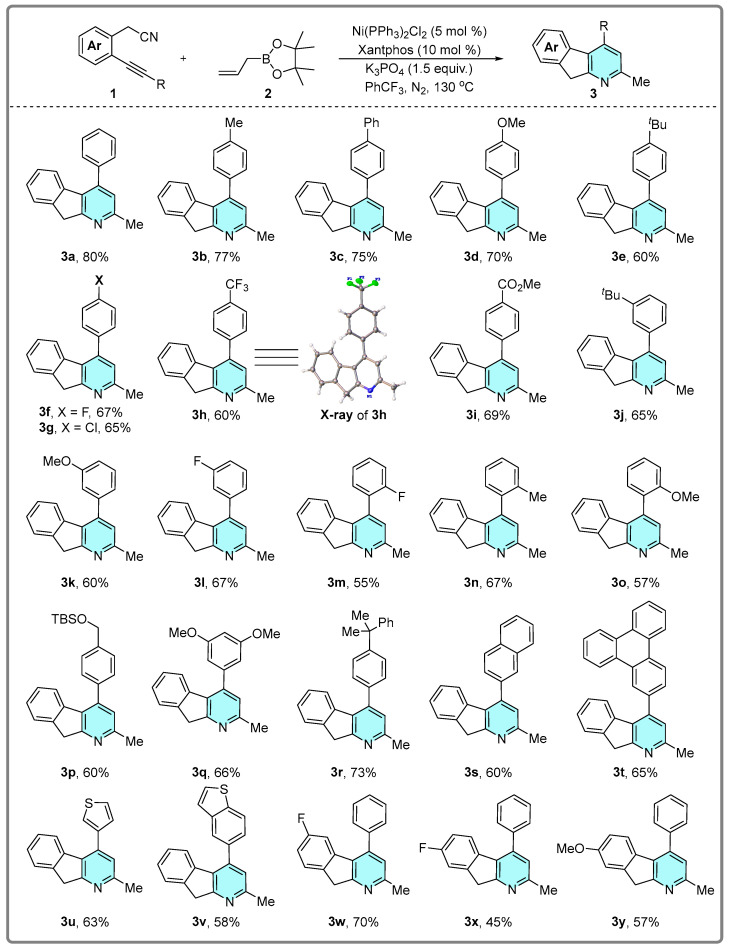
Substrate scope of the Ni-catalyzed [2 + 2 + 2] cycloaddition. The synthetic utility of this reaction was demonstrated through a gram-scale synthesis and product derivatizations. Compound **3a** was successfully synthesized on a gram scale under the optimized conditions, affording the product in good yield (Scheme 3I). Subsequently, compounds **3a** and **3i** were subjected to a copper-mediated oxidation system, yielding the corresponding oxidative products **4a** and **4i** in moderate to good yields (Scheme 3II).

After establishing optimal conditions, the scope of the nickel-catalyzed [2 + 2 + 2] cycloaddition was explored (Scheme 2). Generally, *para*-substituted internal alkynes bearing both electron-donating (Me, OMe, Ph, *^t^*Bu) and electron-withdrawing groups (F, Cl, CF_3_, CO_2_Me) proved compatible, affording tricyclic derivatives in moderate yields (**3b**–**3i**). The constitution of product **3h** was confirmed by X-ray crystallographic analysis (CCDC: 2474292). *meta*-Substituted substrates (*^t^*Bu, OMe, F) also exhibited good reactivity under standard conditions (**3j**–**3l**). Notably, *ortho*-substituted substrates afforded the desired products in lower yields (**3m**–**3o**), indicating steric hindrance influenced reaction efficiency. Significantly, fused pyridine tricyclic derivatives were synthesized from biaryl alkynes bearing diverse substituents (silyl ether, 2-naphthyl, triphenylenyl, thienyl, benzothienyl, etc.), yielding products **3p**–**3v** in moderate yields. Finally, variations in substituents on the acetonitrile-derived phenyl ring were well-tolerated with negligible impact on yields (**3w**–**3y**).

**Scheme 3:**
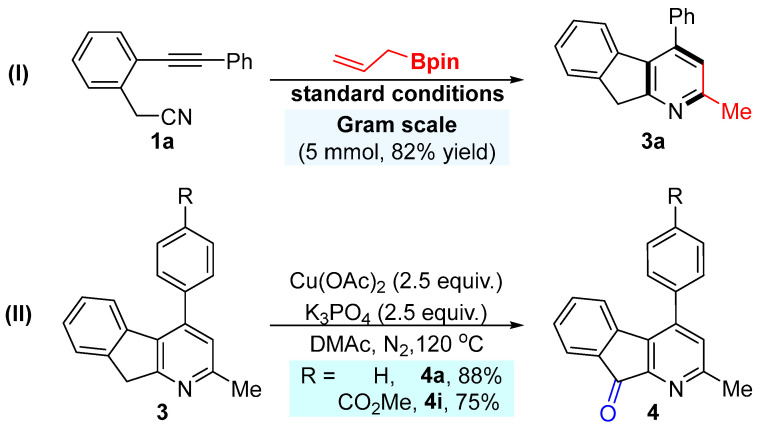
The gram-scale experiment and synthetic utility.

**Scheme 4:**
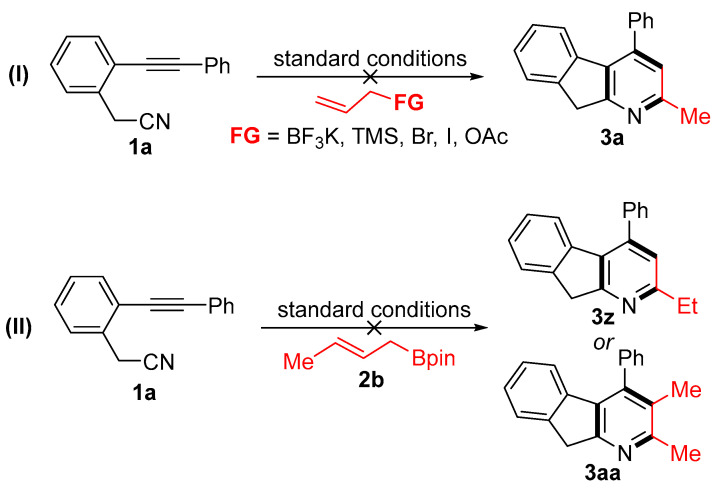
Control experiments.

To gain insight into the reaction mechanism, several control experiments were conducted (Scheme 4). First, various allyl derivatives, such as trifluoroborate, trimethylsilyl, halide, and acetate groups, were used in place of allyl boronate under the standard conditions (Scheme 4I). However, these failed to afford the desired product due to the low conversion of **1a**. Furthermore, crotyl boronate also failed to produce the fused pyridine products **3z** or **3aa** under the optimal conditions (Scheme 4II). These results indicate that both the boronate group and a terminal double bond are essential for the [2 + 2 + 2] cycloaddition.

**Scheme 5:**
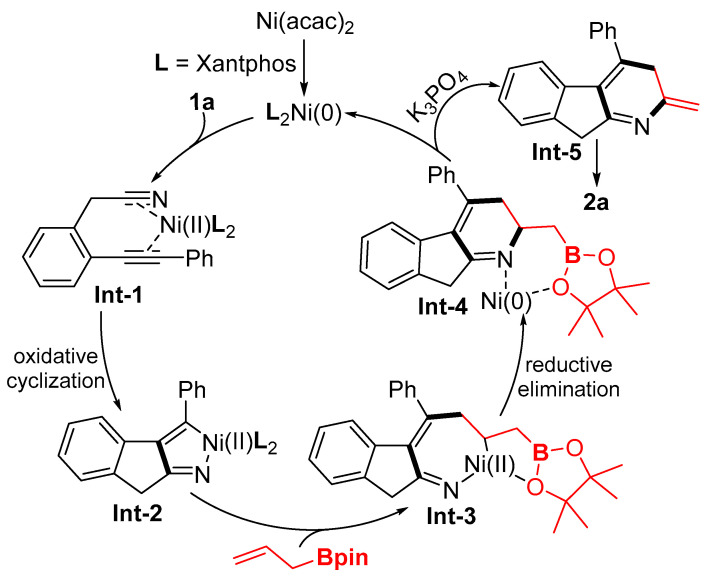
Plausible mechanistic pathways.

Based on experimental results and the previous literature [32,33,37,38], a plausible mechanism is proposed in Scheme 5. Initially, coordination of the substrate’s alkyne group to the nickel(0) catalyst forms intermediate **Int-1**. Subsequent oxidative hetero-cyclometallation then generates azanickelcyclopentadiene intermediate **Int-2**. This species undergoes migration insertion of allyl boronate’s double bond, yielding seven-membered aza-nickelacycle **Int-3**. Reductive elimination of **Int-3** releases both the active nickel(0) species and the six-membered framework complex **Int-4**. Finally, Int-4 undergoes base-facilitated olefin isomerization to afford the target product **3a**.

## 3. Materials and Methods

### 3.1. Materials

All the reagents were obtained from commercial sources and used directly without further purification, unless otherwise noted. Synthetic methods and spectral data for the start substrates were consistent with the methods and data reported in the studies. ^1^H NMR, ^19^F NMR, and ^13^C NMR spectra were recorded on a Bruker AVANCE III 400 MHz. ^1^H NMR, ^19^F NMR, and ^13^C NMR chemical shifts were determined relative to the internal standard TMS at δ 0.0. Chemical shifts (δ) are reported in ppm, and coupling constants (J) are reported in Hertz (Hz).

### 3.2. General Methods for the Preparation of Fused Pyridine Derivatives

To a 25 mL oven-dried Schlenk-tube with magnetic stirrer bar, substrate (**1**, 0.2 mmol), Ni(PPh_3_)Cl_2_ (5 mol%), Xantphos (10 mol%), K_3_PO_4_ (1.5 equiv), allyl boronate (**2**, 1.5 equiv, 0.3 mmol), and PhCF_3_ (2.0 mL) were successively added and vigorously stirred together in 130 °C oil bath under N_2_ atmosphere for 12 h. After the reaction was finished, the mixture was cooled to room temperature. The reaction was quenched with saturated NH_4_Cl aq. and extracted with EtOAc (3 × 25 mL). The combined ethyl acetate layer was washed with brine (25 mL) and dried over anhydrous Na_2_SO_4_. The solvent was removed under vacuum. The crude product was purified by flash column chromatography (eluting with petroleum ether/ethyl acetate) on silica gel to afford the product **3**.

*2-methyl-4-phenyl-9H-indeno [2,1-b]pyridine* (**3a**): Compound **3a** was prepared according to the general procedure using **1a** with allyl borate **2**. Purification by flash column chromatography (petroleum ether/ethyl acetate = 8/1, *v*/*v*) afforded **3a** as a brown solid, m.p. = 127.4—129.6 °C, 41.1 mg, 80% yield. ^1^H NMR (400 MHz, CDCl_3_) δ 7.56—7.46 (m, 6H), 7.26—7.22 (m, 1H), 7.12—7.07 (m, 2H), 7.01 (s, 1H), 4.02 (s, 2H), 2.65 (s, 3H). ^13^C NMR (101 MHz, CDCl_3_) δ 164.77, 155.93, 144.92, 141.38, 139.44, 138.83, 129.56, 128.71, 128.43, 128.41, 126.92, 126.55, 125.04, 122.84, 122.69, 38.85, 24.24. HRMS-ESI (*m*/*z*): calcd for C_19_H_15_N, [M+H]^+^: 258.1283, found, 258.1302.

*2-methyl-4-(p-tolyl)-9H-indeno [2,1-b]pyridine* (**3b**): Compound **3b** was prepared according to the general procedure using **1b** with allyl borate **2**. Purification by flash column chromatography (petroleum ether/ethyl acetate = 8/1, *v*/*v*) afforded **3b** as a brown solid, m.p. = 99.2—103.3 °C, 41.7 mg, 77% yield. ^1^H NMR (400 MHz, CDCl_3_) δ 7.54 (d, *J* = 7.5 Hz, 1H), 7.38 (d, *J* = 7.9 Hz, 2H), 7.32 (d, *J* = 7.8 Hz, 2H), 7.26—7.23 (m, 1H), 7.17 (d, *J* = 7.6 Hz, 1H), 7.11 (t, *J* = 7.5 Hz, 1H), 6.99 (s, 1H), 4.02 (s, 2H), 2.65 (s, 3H), 2.48 (s, 3H). ^13^C NMR (101 MHz, CDCl_3_) δ 164.70, 155.83, 145.09, 141.35, 139.55, 138.27, 135.85, 129.64, 129.38, 128.32, 126.86, 126.51, 125.01, 122.90, 122.83, 38.82, 24.19, 21.40. HRMS-ESI (*m*/*z*): calcd for C_20_H_17_N, [M+H]^+^: 272.1439, found, 272.1432.

*4-([1,1’-biphenyl]-4-yl)-2-methyl-9H-indeno [2,1-b]pyridine* (**3c**): Compound **3c** was prepared according to the general procedure using **1c** with allyl borate **2**. Purification by flash column chromatography (petroleum ether/ethyl acetate = 6/1, *v*/*v*) afforded **3c** as a brown solid, m.p. = 180.1—185.4 °C, 50 mg, 75% yield. ^1^H NMR (400 MHz, CDCl_3_) δ 7.79—7.70 (m, 4H), 7.60—7.55 (m, 3H), 7.50 (dd, *J* = 8.4, 6.9 Hz, 2H), 7.43—7.38 (m, 1H), 7.28 (dd, *J* = 7.5, 1.2 Hz, 1H), 7.25—7.22 (m, 1H), 7.15—7.10 (m, 1H), 7.06 (s, 1H), 4.04 (s, 2H), 2.67 (s, 3H). ^13^C NMR (101 MHz, CDCl_3_) δ 164.81, 155.92, 144.64, 141.40, 141.25, 140.44, 139.41, 137.70, 129.61, 128.97, 128.95, 127.70, 127.37, 127.16, 127.02, 126.61, 125.10, 122.93, 122.77, 38.84, 24.23. HRMS-ESI (*m*/*z*): calcd for C_25_H_19_N, [M+H]^+^: 334.1596, found, 334.1609.

*4-(4-methoxyphenyl)-2-methyl-9H-indeno [2,1-b]pyridine* (**3d**): Compound **3d** was prepared according to the general procedure using **1d** with allyl borate **2**. Purification by flash column chromatography (petroleum ether/ethyl acetate = 3/1, *v*/*v*) afforded **3d** as brown solid, m.p. = 133.2—135.4 °C, 40.2 mg, 70% yield. ^1^H NMR (400 MHz, CDCl_3_) δ 7.57—7.52 (m, 1H), 7.45—7.39 (m, 2H), 7.27 (d, *J* = 1.2 Hz, 1H), 7.24—7.18 (m, 1H), 7.12 (t, *J* = 7.6 Hz, 1H), 7.07—7.03 (m, 2H), 6.99 (s, 1H), 4.02 (s, 2H), 3.92 (s, 3H), 2.65 (s, 3H). ^3^C NMR (101 MHz, CDCl_3_) δ 164.72, 159.81, 155.82, 144.81, 141.35, 139.57, 131.07, 129.73, 129.71, 126.86, 126.52, 125.03, 122.90, 122.84, 114.10, 55.40, 38.83, 24.17. HRMS-ESI (*m*/*z*): calcd for C_25_H_19_N, [M+H]^+^: 288.1388, found, 288.1388.

*4-(4-(tert-butyl)phenyl)-2-methyl-9H-indeno [2,1-b]pyridine* (**3e**): Compound **3e** was prepared according to the general procedure using **1e** with allyl borate **2**. Purification by flash column chromatography (petroleum ether/ethyl acetate = 9/1, *v*/*v*) afforded **3e** as a brown solid, m.p. = 120.2-125.4 °C, 37.6 mg, 60% yield. ^1^H NMR (400 MHz, CDCl_3_) δ 7.57—7.50 (m, 3H), 7.46—7.40 (m, 2H), 7.26—7.23 (m, 1H), 7.20 (dt, *J* = 7.8, 1.0 Hz, 1H), 7.14—7.10 (m, 1H), 7.01 (s, 1H), 4.02 (s, 2H), 2.64 (s, 3H), 1.42 (s, 9H). ^13^C NMR (101 MHz, CDCl_3_) δ 164.72, 155.81, 151.58, 145.05, 141.35, 139.57, 135.77, 129.61, 128.12, 126.86, 126.52, 125.57, 125.00, 122.93, 122.91, 38.84, 34.80, 31.43, 24.17. HRMS-ESI (*m*/*z*): calcd for C_23_H_23_N, [M+H]^+^: 314.1909, found, 314.1928.

*4-(4-fluorophenyl)-2-methyl-9H-indeno [2,1-b]pyridine* (**3f**): Compound **3f** was prepared according to the general procedure using **1f** with allyl borate **2**. Purification by flash column chromatography (petroleum ether/ethyl acetate = 7/1, *v*/*v*) afforded **3f** as a brown solid, m.p. = 159.4—162.2 °C, 36.8 mg, 67% yield. ^1^H NMR (400 MHz, CDCl_3_) δ 7.56 (d, *J* = 7.5 Hz, 1H), 7.49—7.44 (m, 2H), 7.29—7.19 (m, 4H), 7.15—7.06 (m, 2H), 6.98 (s, 1H), 4.02 (s, 2H), 2.66 (s, 3H).^13^C NMR (101 MHz, CDCl_3_) δ 164.85, 162.88 (d, *J* = 247.8 Hz), 156.01, 143.85, 141.41, 139.25, 134.78, 130.24 (d, *J* = 8.3 Hz), 129.64, 126.84 (d, *J* = 45.5 Hz), 125.16, 122.74, 122.67, 115.90, 115.69, 38.83, 24.22. ^19^F NMR (377 MHz, CDCl_3_) δ -113.29. HRMS-ESI (*m*/*z*): calcd for C_19_H_14_FN, [M+H]^+^: 276.1189, found, 276.1188.

*4-(4-chlorophenyl)-2-methyl-9H-indeno [2,1-b]pyridine* (**3g**): Compound **3g** was prepared according to the general procedure using **1g** with allyl borate **2**. Purification by flash column chromatography (petroleum ether/ethyl acetate = 7/1, *v*/*v*) afforded **3g** as a brown solid, m.p. = 133.2—135.4 °C, 37.8 mg, 65% yield. ^1^H NMR (400 MHz, CDCl_3_) δ 7.58—7.49 (m, 3H), 7.46—7.42 (m, 2H), 7.29 (dd, *J* = 7.3, 1.5 Hz, 1H), 7.16—7.09 (m, 2H), 6.98 (s, 1H), 4.03 (s, 2H), 2.66 (s, 3H). ^13^C NMR (101 MHz, CDCl_3_) δ 164.87, 156.02, 141.41, 139.11, 137.21, 129.88, 129.52, 129.02, 127.16, 126.67, 125.19, 122.72, 122.57, 38.81, 24.21.HRMS-ESI (*m*/*z*): calcd for C_19_H_14_ClN, [M+H]^+^: 292.0893, found, 292.0893.

*2-methyl-4-(4-(trifluoromethyl)phenyl)-9H-indeno [2,1-b]pyridine* (**3h**): Compound **3h** was prepared according to the general procedure using **1h** with allyl borate **2**. Purification by flash column chromatography (petroleum ether/ethyl acetate = 7/1, *v*/*v*) afforded **3h** as a brown solid, m.p. = 139—142 °C, 39 mg, 60% yield. ^1^H NMR (400 MHz, CDCl_3_) δ 7.80 (d, *J* = 8.0 Hz, 2H), 7.60 (dd, *J* = 20.3, 7.8 Hz, 3H), 7.31—7.27 (m, 1H), 7.13 (t, *J* = 7.6 Hz, 1H), 7.05—6.97 (m, 2H), 4.05 (s, 2H), 2.68 (s, 3H). ^13^C NMR (101 MHz, CDCl_3_) δ 164.99, 156.14, 143.24, 142.50, 141.46, 138.90, 130.81, 129.39, 128.95, 127.31, 126.74, 125.77 (q, *J* = 3.7 Hz), 125.26, 122.62, 122.41, 38.82, 24.22. ^19^F NMR (377 MHz, CDCl_3_) δ -62.45. HRMS-ESI (*m*/*z*): calcd for C_20_H_14_F_3_N, [M+H]^+^: 326.1157, found, 326.1154.

*methyl 4-(2-methyl-9H-indeno [2,1-b]pyridin-4-yl)benzoate* (**3i**): Compound **3i** was prepared according to the general procedure using **1i** with allyl borate **2**. Purification by flash column chromatography (petroleum ether/ethyl acetate = 2/1, *v*/*v*) afforded **3i** as a brown solid, m.p. = 164.8—168.8 °C, 43.5 mg, 69% yield. ^1^H NMR (400 MHz, CDCl_3_) δ 8.23—8.18 (m, 2H), 7.59—7.55 (m, 3H), 7.27 (td, *J* = 7.4, 1.2 Hz, 2H), 7.10 (td, *J* = 7.6, 1.1 Hz, 1H), 7.05—6.99 (m, 2H), 4.04 (s, 2H), 3.99 (s, 3H), 2.67 (s, 3H). ^13^C NMR (101 MHz, CDCl_3_) δ 166.84, 164.89, 156.03, 143.76, 143.45, 141.43, 138.99, 130.17, 130.05, 129.39, 128.63, 127.22, 126.69, 125.19, 122.74, 122.30, 52.39, 38.81, 24.21. HRMS-ESI (*m*/*z*): calcd for C_21_H_17_NO_2_, [M+H]^+^: 316.1338, found, 316.1317.

*4-(3-(tert-butyl)phenyl)-2-methyl-9H-indeno [2,1-b]pyridine* (**3j**): Compound **3i** was prepared according to the general procedure using **1j** with allyl borate **2**. Purification by flash column chromatography (petroleum ether/ethyl acetate = 8/1, *v*/*v*) afforded **3j** as a brown solid, m.p. = 115.3—118.4 °C, 40.7 mg, 65% yield. ^1^H NMR (400 MHz, CDCl_3_) δ 7.57—7.44 (m, 4H), 7.32—7.27 (m, 1H), 7.25 (dd, *J* = 7.3, 1.5 Hz, 1H), 7.15—7.07 (m, 2H), 7.04 (s, 1H), 4.03 (s, 2H), 2.67 (s, 3H), 1.37 (s, 9H). ^13^C NMR (101 MHz, CDCl_3_) δ 164.71, 155.85, 151.46, 145.56, 141.37, 139.50, 138.31, 129.63, 128.56, 126.91, 126.45, 125.83, 125.44, 125.27, 125.03, 122.96, 122.78, 38.82, 34.92, 31.36, 24.21. HRMS-ESI (*m*/*z*): calcd for C_23_H_23_N, [M+H]^+^: 314.1909, found, 314.1891.

*4-(3-methoxyphenyl)-2-methyl-9H-indeno [2,1-b]pyridine* (**3k**): Compound **3k** was prepared according to the general procedure using **1k** with allyl borate **2**. Purification by flash column chromatography (petroleum ether/ethyl acetate = 3/1, *v*/*v*) afforded **3k** as a brown solid, m.p. = 119.4—120.2 °C, 34.4 mg, 60% yield. ^1^H NMR (400 MHz, CDCl_3_) δ 7.56 (d, *J* = 7.6 Hz, 1H), 7.44 (td, *J* = 7.9, 2.2 Hz, 1H), 7.31—7.24 (m, 2H), 7.17—7.11 (m, 2H), 7.09—7.00 (m, 4H), 4.04 (s, 2H), 3.85 (d, *J* = 2.2 Hz, 3H), 2.67 (d, *J* = 2.2 Hz, 3H). ^13^C NMR (101 MHz, CDCl_3_) δ 164.60, 159.76, 155.74, 144.93, 141.34, 140.06, 139.28, 129.86, 129.65, 127.02, 126.62, 125.05, 122.99, 122.65, 120.73, 114.28, 113.61, 55.40, 38.76, 24.12. HRMS-ESI (*m*/*z*): calcd for C_20_H_17_NO, [M+H]^+^: 288.1388, found, 288.1388.

*4-(3-fluorophenyl)-2-methyl-9H-indeno [2,1-b]pyridine* (**3l**): Compound **3l** was prepared according to the general procedure using **1l** with allyl borate **2**. Purification by flash column chromatography (petroleum ether/ethyl acetate = 7/1, *v*/*v*) afforded **3l** as a brown solid, m.p. = 130.2—135.2 °C, 36.9 mg, 67% yield. ^1^H NMR (400 MHz, CDCl_3_) δ 7.57 (dt, *J* = 7.5, 1.0 Hz, 1H), 7.53—7.47 (m, 1H), 7.31—7.27 (m, 2H), 7.24—7.18 (m, 2H), 7.16—7.07 (m, 2H), 7.00 (s, 1H), 4.04 (s, 2H), 2.67 (s, 3H). ^13^C NMR (101 MHz, CDCl_3_) δ 164.80, 162.85 (d, *J* = 248.0 Hz), 155.94, 143.55, 141.40, 140.84, 138.99, 130.42 (d, *J* = 8.2 Hz), 129.53, 126.95 (d, *J* = 50.2 Hz), 125.17, 124.26 (d, *J* = 2.9 Hz), 122.75, 122.50, 115.68, 115.49 (d, *J* = 5.2 Hz), 115.31, 38.77, 24.14. ^19^F NMR (377 MHz, CDCl_3_) δ -112.17. HRMS-ESI (*m*/*z*): calcd for C_19_H_14_FN, [M+H]^+^: 276.1189, found, 276.1188.

*4-(2-fluorophenyl)-2-methyl-9H-indeno [2,1-b]pyridine* (**3m**): Compound **3m** was prepared according to the general procedure using **1m** with allyl borate **2**. Purification by flash column chromatography (petroleum ether/ethyl acetate = 7/1, *v*/*v*) afforded **3m** as a brown solid, m.p. = 158.9—162.4 °C, 30.2 mg, 55% yield. ^1^H NMR (400 MHz, CDCl_3_) δ 7.58—7.47 (m, 2H), 7.40 (td, *J* = 7.4, 1.9 Hz, 1H), 7.34—7.26 (m, 2H), 7.24 (dd, *J* = 6.0, 1.1 Hz, 1H), 7.13 (td, *J* = 7.6, 1.1 Hz, 1H), 7.04 (s, 1H), 6.95 (d, *J* = 7.8 Hz, 1H), 4.04 (d, *J* = 3.5 Hz, 2H), 2.67 (s, 3H). ^13^C NMR (101 MHz, CDCl_3_) δ 164.62, 159.47 (d, *J* = 247.9 Hz), 155.89, 141.35, 139.27, 138.13, 130.82 (d, *J* = 3.3 Hz), 130.58, 130.50, 130.47, 126.99 (d, *J* = 33.3 Hz), 126.25 (d, *J* = 16.3 Hz), 125.06, 124.57 (d, *J* = 3.7 Hz), 123.01, 122.28, 116.10 (d, *J* = 21.5 Hz), 38.79, 24.23. ^19^F NMR (377 MHz, CDCl_3_) δ -114.66. HRMS-ESI (*m*/*z*): calcd for C_19_H_14_FN, [M+H]^+^: 276.1189, found, 276.1188.

*2-methyl-4-(o-tolyl)-9H-indeno [2,1-b]pyridine* (**3n**): Compound **3n** was prepared according to the general procedure using **1n** with allyl borate **2**. Purification by flash column chromatography (petroleum ether/ethyl acetate = 7/1, *v*/*v*) afforded **3n** as a brown solid, m.p. = 138.9—142.4 °C, 36.3 mg, 67% yield. ^1^H NMR (400 MHz, CDCl_3_) δ 7.55 (dt, *J* = 7.5, 1.0 Hz, 1H), 7.44—7.31 (m, 3H), 7.22 (ddd, *J* = 10.7, 7.5, 1.3 Hz, 3H), 7.08 (td, *J* = 7.6, 1.1 Hz, 1H), 6.97 (s, 1H), 6.63 (dt, *J* = 7.8, 0.9 Hz, 1H), 4.04 (d, *J* = 3.3 Hz, 2H), 2.67 (s, 3H), 2.09 (s, 3H). ^13^C NMR (101 MHz, CDCl_3_) δ 164.31, 155.98, 144.41, 141.20, 139.58, 138.23, 135.54, 130.28, 130.22, 128.45, 128.43, 126.94, 126.25, 124.94, 122.42, 122.23, 38.80, 24.27, 19.78. HRMS-ESI (*m*/*z*): calcd for C_20_H_17_N, [M+H]^+^: 272.1439, found, 272.1432.

*4-(2-methoxyphenyl)-2-methyl-9H-indeno [2,1-b]pyridine* (**3o**): Compound **3o** was prepared according to the general procedure using **1o** with allyl borate **2**. Purification by flash column chromatography (petroleum ether/ethyl acetate = 3/1, *v*/*v*) afforded **3o** as a brown solid, m.p. = 152.1—153.3 °C, 32.8 mg, 57% yield. ^1^H NMR (400 MHz, CDCl_3_) δ 7.55—7.46 (m, 2H), 7.29—7.26 (m, 1H), 7.23 (dd, *J* = 7.5, 1.2 Hz, 1H), 7.13—7.05 (m, 3H), 7.02 (s, 1H), 6.86 (d, *J* = 7.8 Hz, 1H), 4.03 (d, *J* = 6.9 Hz, 2H), 3.69 (s, 3H), 2.66 (s, 3H). ^13^C NMR (101 MHz, CDCl_3_) δ 164.02, 156.53, 141.20, 139.88, 130.26, 130.04, 127.49, 126.77, 126.62, 124.82, 123.23, 122.43, 120.97, 110.94, 55.56, 38.72, 24.17. HRMS-ESI (*m*/*z*): calcd for C_20_H_17_NO, [M+H]^+^: 288.1388, found, 288.1388.

*4-(4-(((tert-butyldimethylsilyl)oxy)methyl)phenyl)-2-methyl-9H-indeno.[2,1-b]pyridine* (**3p**): Compound **3p** was prepared according to the general procedure using **1p** with allyl borate **2**. Purification by flash column chromatography (petroleum ether/ethyl acetate = 10/1, *v*/*v*) afforded **3p** as Brown oil, 48.1 mg, 60% yield. ^1^H NMR (400 MHz, CDCl_3_) δ 7.38 (dt, *J* = 7.5, 1.0 Hz, 1H), 7.33—7.27 (m, 4H), 7.11—7.06 (m, 2H), 6.99—6.89 (m, 2H), 6.84 (s, 1H), 4.71 (s, 2H), 3.86 (s, 2H), 2.49 (s, 3H), 0.82 (s, 9H). ^13^C NMR (101 MHz, CDCl_3_) δ 164.69, 155.82, 144.99, 141.82, 141.35, 139.45, 137.36, 129.67, 128.32, 126.91, 126.52, 126.32, 125.01, 122.93, 122.79, 64.82, 38.80, 26.00, 24.17, 18.49, -5.16. HRMS-ESI (*m*/*z*): calcd for C_26_H_31_NOSi, [M+H]^+^: 402.2253, found, 402.2273.

*4-(3,5-dimethoxyphenyl)-2-methyl-9H-indeno [2,1-b]pyridine* (**3q**): Compound **3q** was prepared according to the general procedure using **1q** with allyl borate **2**. Purification by flash column chromatography (petroleum ether/ethyl acetate = 2/1, *v*/*v*) afforded **3q** as a brown solid, m.p. = 133.4—135.5 °C, 41.9 mg, 66% yield. ^1^H NMR (400 MHz, CDCl_3_) δ 7.56 (d, *J* = 7.5 Hz, 1H), 7.30—7.27 (m, 1H), 7.26—7.21 (m, 1H), 7.15 (t, *J* = 7.5 Hz, 1H), 7.03 (s, 1H), 6.63—6.58 (m, 3H), 4.03 (s, 2H), 3.83 (s, 6H), 2.66 (s, 3H). ^13^C NMR (101 MHz, CDCl_3_) δ 164.68, 160.98, 155.84, 144.86, 141.34, 140.71, 139.28, 129.49, 126.98, 126.66, 125.03, 123.12, 122.40, 106.24, 100.62, 55.54, 38.80, 24.20. HRMS-ESI (*m*/*z*): calcd for C_21_H_19_NO_2_, [M+H]^+^: 318.1494, found, 318.1496.

*2-methyl-4-(4-(2-phenylpropan-2-yl)phenyl)-9H-indeno [2,1-b]pyridine* (**3r**): Compound **3r** was prepared according to the general procedure using **1r** with allyl borate **2**. Purification by flash column chromatography (petroleum ether/ethyl acetate = 5/1, *v*/*v*) afforded **3r** as a brown solid, m.p. = 130.8—131.8 °C, 54.7 mg, 73% yield. ^1^H NMR (400 MHz, CDCl_3_) δ 7.55 (d, *J* = 7.5 Hz, 1H), 7.44—7.35 (m, 4H), 7.33 (d, *J* = 5.2 Hz, 4H), 7.24—7.20 (m, 1H), 7.19—7.09 (m, 2H), 7.02 (s, 1H), 4.02 (s, 2H), 2.65 (s, 3H), 1.78 (s, 6H). ^13^C NMR (101 MHz, CDCl_3_) δ 164.68, 155.79, 151.16, 150.46, 144.98, 141.36, 139.50, 135.98, 129.65, 128.14, 128.11, 127.15, 126.91, 126.86, 126.52, 125.84, 125.04, 122.88, 43.06, 38.82, 30.81, 24.18. HRMS-ESI (*m*/*z*): calcd for C_28_H_25_N, [M+H]^+^: 376.2065, found, 376.2085.

*2-methyl-4-(naphthalen-2-yl)-9H-indeno [2,1-b]pyridine* (**3s**): Compound **3s** was prepared according to the general procedure using **1s** with allyl borate **2**. Purification by flash column chromatography (petroleum ether/ethyl acetate = 10/1, *v*/*v*) afforded **3s** as a brown solid, m.p. = 138.0—142.8 °C, 36.8 mg, 60% yield. ^1^H NMR (400 MHz, CDCl_3_) δ 8.02—7.95 (m, 3H), 7.93—7.89 (m, 1H), 7.63—7.56 (m, 4H), 7.26 (dd, *J* = 14.7, 1.4 Hz, 2H), 7.14—7.02 (m, 3H), 4.09 (s, 2H), 2.70 (s, 3H). ^13^C NMR (101 MHz, CDCl_3_) δ 164.74, 155.86, 141.39, 139.35, 136.24, 133.37, 133.10, 128.40, 128.28, 127.92, 127.43, 127.04, 126.66, 126.64, 126.51, 125.08, 123.02, 122.96, 38.84, 24.20. HRMS-ESI (*m*/*z*): calcd for C_25_H_17_N, [M+H]^+^: 308.1439, found, 308.1460.

*2-methyl-4-(triphenylen-2-yl)-9H-indeno [2,1-b]pyridine* (**3t**): Compound **3t** was prepared according to the general procedure using **1t** with allyl borate **2**. Purification by flash column chromatography (petroleum ether/ethyl acetate = 5/1, *v*/*v*) afforded **3t** as a brown solid, m.p. = 143—147 °C, 52.9 mg, 65% yield. ^1^H NMR (400 MHz, CDCl_3_) δ 8.90—8.53 (m, 7H), 7.83—7.55 (m, 7H), 7.27—7.15 (m, 4H), 7.02 (t, *J* = 7.7 Hz, 1H), 4.11 (s, 2H), 2.76—2.70 (m, 3H). ^13^C NMR (101 MHz, CDCl_3_) δ 164.94, 156.06, 144.90, 141.45, 139.44, 137.48, 130.08, 130.05, 130.03, 129.81, 129.78, 129.55, 129.45, 127.68, 127.66, 127.50, 127.46, 127.43, 127.06, 126.74, 125.14, 123.88, 123.51, 123.48, 123.45, 123.36, 122.88, 122.85, 38.91, 24.30. HRMS-ESI (*m*/*z*): calcd for C_31_H_21_N, [M+H]^+^: 408.1751, found, 408.1765.

*2-methyl-4-(thiophen-3-yl)-9H-indeno [2,1-b]pyridine* (**3u**): Compound **3u** was prepared according to the general procedure using **1u** with allyl borate **2**. Purification by flash column chromatography (petroleum ether/ethyl acetate = 4/1, *v*/*v*) afforded **3u** as a brown solid, m.p. = 138.9—142.2 °C, 33.2 mg, 63% yield. ^1^H NMR (400 MHz, CDCl_3_) δ 7.58—7.54 (m, 1H), 7.51 (dd, *J* = 4.9, 3.0 Hz, 1H), 7.45 (dd, *J* = 3.0, 1.3 Hz, 1H), 7.30—7.27 (m, 2H), 7.20—7.15 (m, 1H), 7.05 (s, 1H), 4.02 (s, 2H), 2.65 (s, 3H). ^13^C NMR (101 MHz, CDCl_3_) δ 141.29, 139.32, 139.10, 128.17, 127.11, 126.73, 126.38, 125.09, 123.81, 122.91, 122.76, 38.74, 24.01. HRMS-ESI (*m*/*z*): calcd for C_17_H_13_NS, [M+H]^+^: 264.0847, found, 264.0823.

*4-(benzo[b]thiophen-5-yl)-2-methyl-9H-indeno [2,1-b]pyridine* (**3v**): Compound **3v** was prepared according to the general procedure using **1v** with allyl borate **2**. Purification by flash column chromatography (petroleum ether/ethyl acetate = 5/1, *v*/*v*) afforded **3v** as **a** brown solid, m.p. = 147.9—150.2 °C, 36.3 mg, 58% yield. ^1^H NMR (400 MHz, CDCl_3_) δ 8.02 (d, *J* = 8.1 Hz, 1H), 7.94 (d, *J* = 1.7 Hz, 1H), 7.58—7.54 (m, 2H), 7.47 (dt, *J* = 8.4, 1.6 Hz, 1H), 7.41 (d, *J* = 5.4 Hz, 1H), 7.25 (dd, *J* = 7.4, 1.8 Hz, 1H), 7.08 (dt, *J* = 7.9, 5.4 Hz, 3H), 4.05 (s, 2H), 2.68 (d, *J* = 1.2 Hz, 3H). ^13^C NMR (101 MHz, CDCl_3_) δ 164.77, 155.89, 145.02, 141.39, 139.91, 139.77, 139.43, 134.96, 129.80, 127.53, 126.99, 126.59, 125.08, 124.79, 124.09, 123.36, 123.11, 122.90, 122.78, 38.85, 24.24. HRMS-ESI (*m*/*z*): calcd for C_21_H_15_NS, [M+H]^+^: 314.1003, found, 314.1021.

*6-fluoro-2-methyl-4-phenyl-9H-indeno [2,1-b]pyridine* (**3w**): Compound **3w** was prepared according to the general procedure using **1w** with allyl borate **2**. Purification by flash column chromatography (petroleum ether/ethyl acetate = 7/1, *v*/*v*) afforded **3w** as a brown solid, m.p. = 120.3—124.5 °C, 38.5 mg, 70% yield. ^1^H NMR (400 MHz, CDCl_3_) δ 7.53 (pd, *J* = 4.7, 1.7 Hz, 3H), 7.50—7.44 (m, 3H), 7.03 (s, 1H), 6.97—6.91 (m, 1H), 6.76 (dd, *J* = 9.9, 2.5 Hz, 1H), 3.98 (s, 2H), 2.66 (s, 3H). ^13^C NMR (101 MHz, CDCl_3_) δ 165.45, 161.79 (d, *J* = 242.5 Hz), 156.58, 145.29, 141.09 (d, *J* = 9.4 Hz), 138.17, 136.65, 136.62, 129.03, 129.00, 128.88, 128.73, 128.29, 125.92 (d, *J* = 8.9 Hz), 122.80, 113.83 (d, *J* = 23.0 Hz), 109.90 (d, *J* = 24.3 Hz), 38.24, 24.28. ^19^F NMR (377 MHz, CDCl_3_) δ -115.19. HRMS-ESI (*m*/*z*): calcd for C_19_H_14_FN, [M+H]^+^: 276.1189, found, 276.1188.

*7-fluoro-2-methyl-4-phenyl-9H-indeno [2,1-b]pyridine* (**3x**): Compound **3x** was prepared according to the general procedure using **1x** with allyl borate **2**. Purification by flash column chromatography (petroleum ether/ethyl acetate = 6/1, *v*/*v*) afforded **3x** as a brown solid, m.p. = 123.4—125.5 °C, 24.7 mg, 45% yield. ^1^H NMR (400 MHz, CDCl_3_) δ 7.58—7.45 (m, 6H), 7.04 (s, 1H), 6.95 (td, *J* = 8.7, 2.5 Hz, 1H), 6.76 (dd, *J* = 9.9, 2.5 Hz, 1H), 3.99 (s, 2H), 2.67 (s, 3H). ^13^C NMR (101 MHz, CDCl_3_) δ 165.39, 161.79 (d, *J* = 242.4 Hz), 156.52, 145.36, 141.06 (d, *J* = 9.2 Hz), 138.14, 136.61, 128.89, 128.75, 128.28, 125.93 (d, *J* = 9.1 Hz), 122.86, 113.86 (d, *J* = 22.9 Hz), 109.91 (d, *J* = 24.4 Hz), 38.20, 24.23.^19^F NMR (377 MHz, CDCl_3_) δ -115.19. HRMS-ESI (*m*/*z*): calcd for C_19_H_14_FN, [M+H]^+^: 276.1189, found, 276.1188.

*7-methoxy-2-methyl-4-phenyl-9H-indeno [2,1-b]pyridine* (**3y**): Compound **3y** was prepared according to the general procedure using **1y** with allyl borate **2**. Purification by flash column chromatography (petroleum ether/ethyl acetate = 3/1, *v*/*v*) afforded **3y** as a brown solid, m.p. = 117.3—120.5 °C, 32.7 mg, 57% yield. ^1^H NMR (400 MHz, CDCl_3_) δ 7.57—7.48 (m, 2H), 7.40 (td, *J* = 7.4, 1.9 Hz, 1H), 7.33—7.27 (m, 2H), 7.26—7.23 (m, 1H), 7.13 (td, *J* = 7.6, 1.1 Hz, 1H), 7.04 (s, 1H), 6.95 (d, *J* = 7.8 Hz, 1H), 4.04 (d, *J* = 3.5 Hz, 2H), 2.67 (s, 3H). ^13^C NMR (101 MHz, CDCl_3_) δ 192.69, 164.06, 159.19, 143.31, 138.81, 132.15, 129.83, 128.73, 128.42, 128.40, 123.64, 122.68, 112.81, 110.44, 55.46, 38.88, 23.94. HRMS-ESI (*m*/*z*): calcd for C_20_H_17_NO, [M+H]^+^: 288.1388, found, 288.1388.

### 3.3. General Procedure for the Derivatization of Pyridine Products

To a 25 mL Schlenk tube with a magnetic stirrer bar, substrates (**3**, 0.2 mmol), Cu(OAc)_2_ (1.5 equiv, 0.3 mmol), K_3_PO_4_ (2.5 equiv), and DMAc (2.0 mL) were successively added and vigorously stirred together in a 120 °C oil bath under N_2_ atmosphere for 8 h. After the reaction was finished, the mixture was cooled to room temperature. The reaction was quenched with saturated NH_4_Cl aq. and extracted with EtOAc (3 × 20 mL). The combined ethyl acetate layer was washed with brine (15 mL) and dried over anhydrous Na_2_SO_4_. The solvent was removed under vacuum. The crude product was purified by flash column chromatography (eluting with petroleum ether/ethyl acetate) on silica gel to afford the product **4**.

*2-methyl-4-phenyl-9H-indeno [2,1-b]pyridin-9-one* (**4a**): Compound **4a** was prepared according to the general procedure using **3a**. Purification by flash column chromatography (petroleum ether/ethyl acetate = 3/1, *v*/*v*) afforded **4a** as a light yellow solid, m.p. = 116.5—119.5 °C, 47.7 mg, 88% yield. ^1^H NMR (400 MHz, Chloroform-*d*) δ 7.71 (dd, *J* = 5.7, 3.0 Hz, 1H), 7.57—7.46 (m, 5H), 7.26—7.23 (m, 2H), 7.07 (s, 1H), 6.90 (dd, *J* = 6.4, 2.6 Hz, 1H), 2.66 (s, 3H). ^13^C NMR (101 MHz, Chloroform-*d*) δ 193.02, 159.49, 153.24, 145.59, 142.21, 137.26, 135.11, 134.70, 132.45, 129.20, 129.11, 128.99, 128.17, 128.05, 124.69, 123.14, 24.12. HRMS-ESI (*m*/*z*): calcd for C_19_H_13_NO, [M+H]^+^: 272.1070, found, 272.1061.

*methyl 4-(2-methyl-9-oxo-9H-indeno [2,1-b]pyridin-4-yl)benzoate* (**4i**): Compound **4i** was prepared according to the general procedure using **3i**. Purification by flash column chromatography (petroleum ether/ethyl acetate = 1/1, *v*/*v*) afforded **4i** as a light yellow solid, m.p. = 112.5—114.5 °C, 49.3 mg, 75% yield. ^1^H NMR (400 MHz, Chloroform-*d*) δ 8.28—8.17 (m, 2H), 7.73 (d, *J* = 6.6 Hz, 1H), 7.61—7.54 (m, 2H), 7.28 (d, *J* = 9.0 Hz, 2H), 7.08 (s, 1H), 6.84 (d, *J* = 6.8 Hz, 1H), 4.00 (d, *J* = 1.5 Hz, 3H), 2.68 (s, 3H). ^13^C NMR (101 MHz, Chloroform-*d*) δ 192.69, 166.54, 159.72, 153.34, 144.38, 141.82, 141.80, 135.21, 134.48, 132.44, 130.84, 130.26, 129.47, 128.40, 127.54, 124.87, 123.04, 52.48, 24.15. HRMS-ESI (*m*/*z*): calcd for C_21_H_15_NO_3_, [M+H]^+^: 330.1125, found, 330 1112.

## 4. Conclusions

In summary, we have developed a nickel-catalyzed [2 + 2 + 2] cycloaddition of alkynes, nitriles, and allyl boronates using a concise and stable catalytic system. This protocol employs readily available starting materials, enabling the efficient synthesis of fused pyridine derivatives with good functional group compatibility and excellent regioselectivity. Mechanistic studies revealed the essential roles of the terminal double bond and the Bpin group. Collectively, these findings provide fundamental insights into the [2 + 2 + 2] cycloaddition mechanism and the reactivity of azametallacyclopentadienes, thereby opening new avenues for metal-catalyzed cycloadditions of unsaturated compounds.

## Figures and Tables

**Table 1:** Optimization of the reaction conditions.

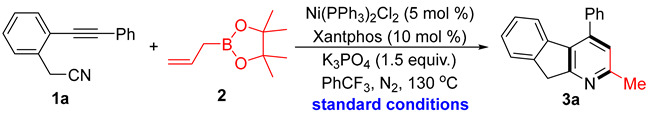		
Entry *^a^*	Variation from Standard Conditions	Yield of 3a *^b^*
1	none	84% (80%)
2	w/o Ni(PPh_3_)_2_Cl_2_	N.R.
3	w/o XantPhos	N.R.
4	w/o K_3_PO_4_	N.R.
5	Ni(acac)_2_ instead of Ni(PPh_3_)_2_Cl_2_	55%
6	DPEphos instead of XantPhos	30%
7	dppp instead of XantPhos	35%
8	dppf instead of XantPhos	<5%
9	Cy_3_P instead of XantPhos	32%
10	Na_3_PO_4_ instead of K_3_PO_4_	15%
11	Toluene instead of PhCF_3_	70%
12	CH_3_CN instead of PhCF_3_	25%
13	under air atmosphere	72%
14	reaction temperature 120 °C	70%

*^a^* Conditions: **1a** (0.2 mmol), **2** (1.5 equiv), catalyst (5 mol %), ligand (10 mol %), base (1.5 equiv), solvent (2.0 mL) under N_2_ atmosphere at 130 °C for 24 h in seal tube; *^b^* Yields were determined by ^1^H NMR with CH_2_Br_2_ as internal standard, isolated yield is in the parentheses; N.R. = no reaction.

## Data Availability

Data are contained within the article and Supplementary Materials.

## Supplementary Materials

The following supporting information can be downloaded at: https://www.mdpi.com/article/10.3390/molecules30173629/s1, the X-ray of **3h**, NMR data and spectra of the catalytic products. References [36,37,39,40,41,42,43] are cited in the Supplementary Materials.
