# Supplementary material for: Ni-Catalyzed [2 + 2 + 2] Cycloaddition via the Capture of Azametallacyclopentadienes with Allyl Boronate: Facile Access to Fused Pyridine Derivatives
Source: Molecules. 2025 Sep 5;30(17):3629. doi: 10.3390/molecules30173629 (PMC12430644; doi:10.3390/molecules30173629)

# Supporting Information

## Ni-Catalyzed [2+2+2] Cycloaddition via the Capture of Azametallacyclopentadienes with Allyl Boronate: Facile Access to Fused Pyridine Derivatives

Kesi Du[1,+], Tao Zhu[2,+], Guangyu Li[2], Taohong Shi[2], Chunsheng Li[3,*], Siting Hu[3], Ruiran Gao[1], Zhao-Yang Wang[4,*], Jiuzhong Huang[2,*]

[1] Guizhou Provincial Engineering Technology Research Center for Chemical Drug R&D, School of Pharmacy, Guizhou Medical University, Guiyang 550004, China;
[2] Jiangxi Province Key Laboratory of Pharmacology of Traditional Chinese Medicine, School of Pharmacy, Gannan Medical University, Ganzhou 341000, China;
[3] School of Environmental and Chemical Engineering, Zhaoqing University, Zhaoqing 526061, China;
[4] School of Chemistry, South China Normal University, Guangzhou 510006, China;
* Correspondence: huangjz@gmu.edu.cn; wangzy@scnu.edu.cn; lichunsheng@zqu.edu.cn
+ Equal contribution

## Table of Contents

**General information ........................................................................2**

**General procedure for the synthesis of reaction substrates..................2**

**Characterization data for new substrates ............................................3**

**Optimization conditions of the [2+2+2] cycloaddition reaction ...........4**

**X-ray Crystallographic data of compound 3h ......................................5**

**NMR Spectra for all compounds ...........................................................7**

## General information

$^{1}$H NMR, $^{13}$C NMR, $^{11}$B NMR and $^{19}$F NMR spectra were recorded on a Bruker Ascend™ 400M spectrometer at ambient temperature in chloroform-*d* and tetramethylsilane (TMS) as an internal standard unless otherwise noted, the chemical shifts of $^{1}$H NMR, $^{13}$C NMR are referenced to signals at 7.26 and 77.0 ppm, respectively. Data for $^{1}$H NMR are reported as follows: chemical shift (δ ppm), multiplicity, integration, and coupling constant (Hz). Data for $^{13}$C NMR are reported in terms of chemical shift (δ ppm), multiplicity, and coupling constant (Hz). Data for $^{11}$B NMR and $^{19}$F NMR are reported in terms of chemical shift (δ ppm). Multiplicity was indicated as follows: s (singlet), d (doublet), t (triplet), q (quartet), m (multiplet), td (triplet of doublets), dt (doublet of triplets), ddd (doublet of doublet of doublets). The data of HRMS were carried out on a high-resolution mass spectrometer (LCMS-IT-TOF). Melting points were determined with Büchi Melting Point B-545 instrument. The data of X-ray were performed on a BRUKER Single Crystal X-Ray Diffractometer, Germany (model of the instrument-AXS D8 Quest System). TLC was performed by using commercially prepared 200-300 mesh silica gel plates and visualization effected at 254 nm. Unless stated otherwise, all reagents and solvents were purchased from commercial suppliers and used without further purification.

## General procedure for the synthesis of reaction substrates

Synthetic methods and spectral data for **1a**[1], **1b**[2], **1c**[3], **1d**[1], **1e**[1], **1f**[2], **1g**[2], **1h**[2], **1i**[4], **1j**[5], **1k**[6], **1l**[2], **1m**[2], **1n**[3], **1o**[7], **1p**[2], **1q**[5], **1r**[7], **1s**[5], **1t**[4], **1u**[3], **1v**[3] were consistent with the methods and data reported in the literatures.

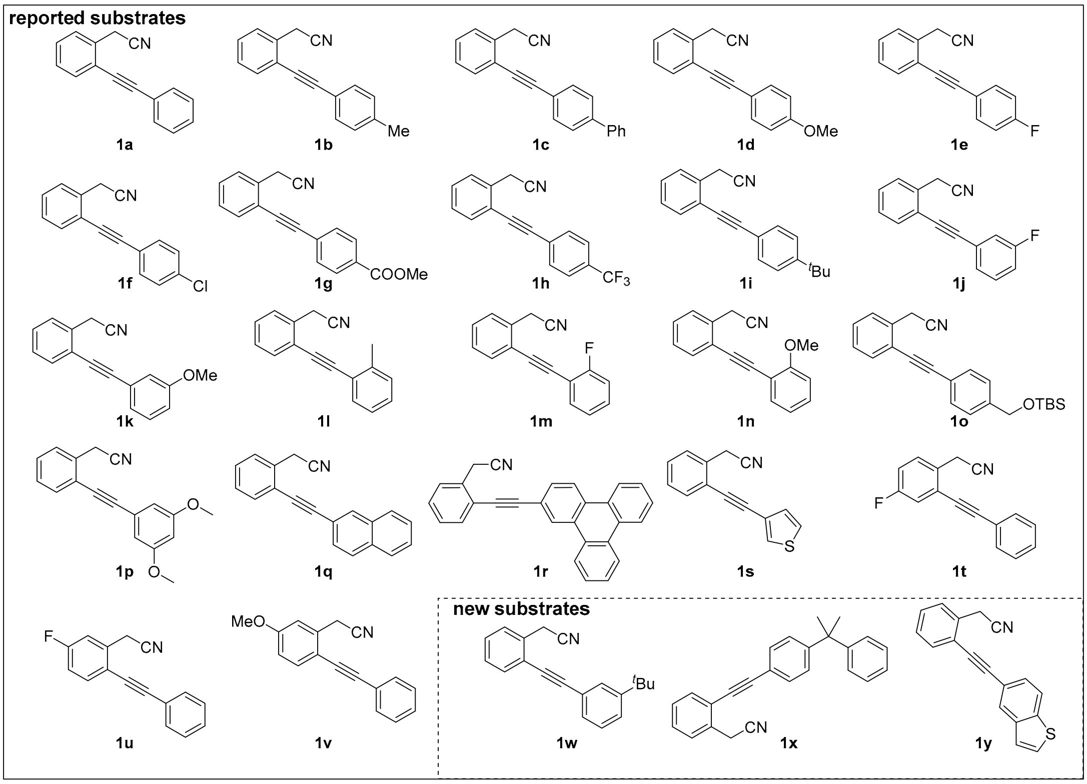

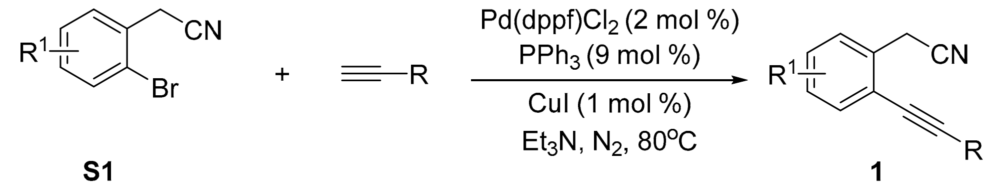

To a 25 mL sealed tube with magnetic stirrer bar, 2-(2-bromophenyl)acetonitrile (**S1**, 5 mmol), terminal alkyne (1.2 equiv), Pd(dppf)$Cl_2$ (2 mol %), $PPh_3$ (9 mol %), CuI (1 mol %), and $Et_3N$ (15 mL) were successively added and vigorously stirred together in 80 °C oil bath under $N_2$ atmosphere. After the reaction was finished, the mixture was cooled to room temperature. The reaction was quenched with saturated $NH_4Cl$ aq. and extracted with EtOAc (3×35 mL). The combined ethyl acetate layer was washed with brine (35 mL) and dried over anhydrous $Na_2SO_4$. The solvent was removed under vacuum. The crude product was purified by flash column chromatography (eluting with petroleum ether/ethyl acetate) on silica gel to afford the product **1**.

## Characterization data for new substrates

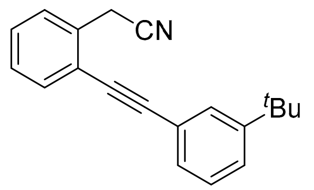

**2-(2-((3-(tert-butyl)phenyl)ethynyl)phenyl)acetonitrile (1w)**

Compound **1w** was prepared according to the general procedure using 2-(2-bromophenyl)acetonitrile with 1-(tert-butyl)-3-ethynylbenzene. Purification by flash column chromatography (petroleum ether/ethyl acetate = 8/1, v/v) afforded **1w** as brown solid, m.p. = 170.5-173.6 °C. $^1$H NMR (400 MHz, $CDCl_3$) δ 7.60 – 7.53 (m, 2H), 7.48 – 7.45 (m, 1H), 7.41 – 7.27 (m, 6H), 3.94 (s, 2H), 1.33 (s, 9H). $^{13}$C NMR (101 MHz, $CDCl_3$) δ 151.54, 133.91, 133.72, 132.43, 131.84, 131.83, 129.03, 128.91, 128.83, 128.66, 128.57, 128.41, 128.24, 126.28, 122.99, 122.18, 117.63, 96.39, 85.57, 34.78, 31.31, 22.88. HRMS-ESI (m/z): calcd for $C_{20}H_{19}N$, $[M+H]^+$: 274.1596, found, 274.1593.

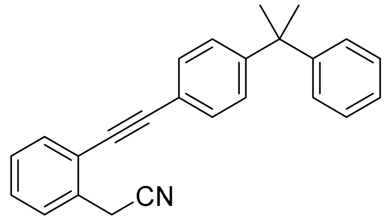

**2-(2-((4-(2-phenylpropan-2-yl)phenyl)ethynyl)phenyl)acetonitrile (1x)**

Compound **1x** was prepared according to the general procedure using 2-(2-bromophenyl)acetonitrile with 1-ethynyl-4-(2-phenylpropan-2-yl)benzene. Purification by flash column chromatography (petroleum ether/ethyl acetate = 7/1, v/v) afforded **1x** as brown oil. $^1$H NMR (400 MHz, $CDCl_3$) δ 7.48 (dd, $J$ = 7.5, 1.6 Hz, 1H), 7.43 – 7.36 (m, 3H), 7.32 – 7.27 (m, 1H), 7.25 – 7.09 (m, 8H), 3.88 (s, 2H), 1.61 (s, 6H). $^{13}$C NMR (101 MHz, $CDCl_3$) δ 150.81, 148.96, 131.27, 130.58, 130.27, 127.86, 127.11, 127.08, 126.01, 125.71, 124.82, 121.89, 118.64, 116.46, 94.74, 42.07, 29.50, 21.74. HRMS-ESI (m/z): calcd for $C_{25}H_{21}N$, $[M+H]^+$: 336.1752, found, 336.1751.

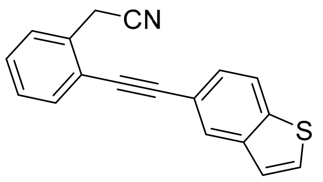


**2-(2-(benzo[b]thiophen-5-ylethynyl)phenyl)acetonitrile (1y)**

Compound **1y** was prepared according to the general procedure using 2-(2-bromophenyl)acetonitrile with 5-ethynylbenzo[b]thiophene. Purification by flash column chromatography (petroleum ether/ethyl acetate = 3/1, v/v) afforded **1y** as white solid, m.p. = 139.5-144.1 °C. $^{1}$H NMR (400 MHz, $CDCl_3$) δ 8.03 (d, $J$ = 1.4 Hz, 1H), 7.86 (d, $J$ = 8.4 Hz, 1H), 7.59 (dd, $J$ = 7.3, 1.8 Hz, 1H), 7.52 – 7.46 (m, 3H), 7.40 – 7.31 (m, 3H), 3.99 (s, 2H). $^{13}$C NMR (101 MHz, $CDCl_3$) δ 140.23, 139.62, 132.38, 131.68, 129.00, 128.24, 127.72, 127.13, 127.01, 123.75, 122.95, 122.69, 118.37, 117.58, 96.13, 85.63, 22.89. HRMS-ESI (m/z): calcd for $C_{18}H_{11}NS$, $[M+H]^+$: 274.0690, found, 274.0664.

## Optimization conditions of the [2+2+2] cycloaddition reaction

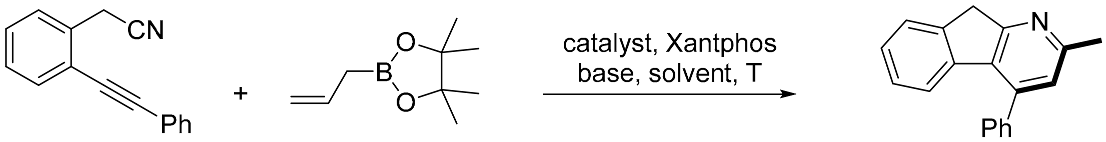


**1a** **2** **3a**

| Entry $^{a}$ | catalyst | base | solvent | yield of 3a (%) |
|---|---|---|---|---|
| 1 | $Ni(PPh_3)_2Cl_2$ | $K_3PO_4$ | Toluene | 70 |
| 2 | $Ni(PPh_3)_2Cl_2$ | $K_3PO_4$ | $PhCF_3$ | 80 |
| 3 | $Ni(PPh_3)_2Cl_2$ | $K_3PO_4$ | cyclohexane | 75 |
| 4 | $Ni(PPh_3)_2Cl_2$ | $K_3PO_4$ | THF | 72 |
| 5 | $Ni(PPh_3)_2Cl_2$ | $K_3PO_4$ | 1,4-dioxane | N.R. |
| 6 | $Ni(PPh_3)_2Cl_2$ | $K_3PO_4$ | $CH_3CN$ | 25 |
| 7 | $Ni(PPh_3)_2Cl_2$ | $K_3PO_4$ | DMF | N.R. |
| 8 | $Ni(PPh_3)_2Cl_2$ | $K_3PO_4$ | DCE | N.R. |
| 9 | $Ni(PPh_3)_2Cl_2$ | $K_3PO_4$ | DMSO | N.R. |
| 10 | $Ni(PPh_3)_2Cl_2$ | $K_3PO_4$ | DME | 65 |
| 11 | $Ni(acac)_2$ | $K_3PO_4$ | $PhCF_3$ | 55 |
| 12 | $Ni(OAc)_2{\cdot}4H_2O$ | $K_3PO_4$ | $PhCF_3$ | 76 |
| 13 | $NiCl_2{\cdot}6H_2O$ | $K_3PO_4$ | $PhCF_3$ | 50 |
| 14 | $Ni(Cy_3P)_2Cl_2$ | $K_3PO_4$ | $PhCF_3$ | 79 |
| 15 | $Ni(COD)_2$ | $K_3PO_4$ | $PhCF_3$ | N.R. |
| 16 | $Ni(PPh_3)_4$ | $K_3PO_4$ | $PhCF_3$ | 60 |
| 17 | $Pd(PPh_3)_2Cl_2$ | $K_3PO_4$ | $PhCF_3$ | N.R. |
| 18 | $Pd(PPh_3)_4$ | $K_3PO_4$ | $PhCF_3$ | N.R. |
| 19 | $Ni(PPh_3)_2Cl_2$ | $Li_3PO_4$ | $PhCF_3$ | N.R. |
| 20 | $Ni(PPh_3)_2Cl_2$ | $Na_3PO_4$ | $PhCF_3$ | 15 |

| | | | | |
|---|---|---|---|---|
| 21 | $Ni(PPh_3)_2Cl_2$ | $K_2CO_3$ | $PhCF_3$ | 30 |
| 22 | $Ni(PPh_3)_2Cl_2$ | NaOH | $PhCF_3$ | N.R. |
| 23 | $Ni(PPh_3)_2Cl_2$ | $Cs_2CO_3$ | $PhCF_3$ | 20 |
| 24 | $Ni(PPh_3)_2Cl_2$ | $^{t}$BuOK | $PhCF_3$ | trace |
| 25 | $Ni(PPh_3)_2Cl_2$ | MeOK | $PhCF_3$ | trace |
| 26 | $Ni(PPh_3)_2Cl_2$ | KF | $PhCF_3$ | N.R. |
| 27[b] | $Ni(PPh_3)_2Cl_2$ | $K_3PO_4$ | $PhCF_3$ | N.R. |
| 28[c] | $Ni(PPh_3)_2Cl_2$ | $K_3PO_4$ | $PhCF_3$ | 70 |
| 29[d] | $Ni(PPh_3)_2Cl_2$ | $K_3PO_4$ | $PhCF_3$ | 50 |
| 30[e] | $Ni(PPh_3)_2Cl_2$ | $K_3PO_4$ | $PhCF_3$ | 72 |

[a] Conditions: **1a** (0.2 mmol), catalyst (10 mol %), **2** (1.5 equiv), Xantphos (10 mol %), base (1.5 equiv), solvent ( 2.0 mL) under $N_2$ atmosphere at 130°C for 24 h in seal tube; Yields were determined by $^{1}$H NMR with $CH_2Br_2$ as internal standard, isolated yield is in the parentheses; N.D. = not detected; N.R. = not reaction; [b] at 90°C; [c] at 120°C ;[d] reacted for 6 h; [e] Under air atmosphere.

## X-ray Crystallographic data of compound 3h

Crystal of compound **3h** were prepared in a solvent mixture of DCM and petroleum ether (v/v = 1/1) respectively. **3h** (20 mg) were firstly dissolved in DCM (1 mL) in a vial, then petroleum ether (1 mL) was added dropwise to it. The vial was not fully screwed down and the sample was carefully setting in room temperature. The crystal was obtained in about 48 h.
The X-ray crystallographic structure for **3h**. ORTEP representation with 50% probability thermal ellipsoids. Crystal data have been deposited to CCDC number 2474292.

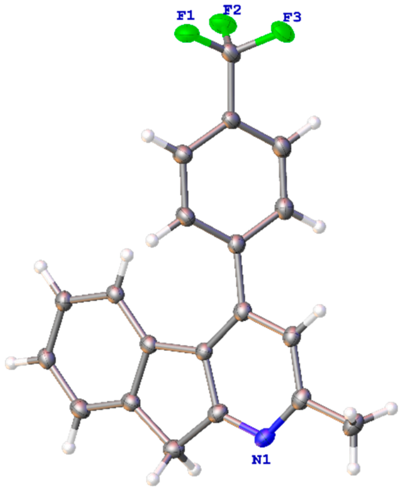


**Table S1 Crystal data and structure refinement for 3h**

| | |
|---|---|
| Identification code | **3h** |
| Empirical formula | $C_{20}H_{14}F_3N$ |
| Formula weight | 325.32 |
| Temperature/K | 100.00(10) |
| Crystal system | orthorhombic |
| Space group | $P2_12_12_1$ |
| a/Å | 4.62760(10) |

| | |
|---|---|
| b/Å | 12.3432(2) |
| c/Å | 26.5525(5) |
| α/° | 90 |
| β/° | 90 |
| γ/° | 90 |
| Volume/Å$^3$ | 1516.66(5) |
| Z | 4 |
| $\rho_{calc}$g/cm$^3$ | 1.425 |
| μ/mm$^{-1}$ | 0.916 |
| F(000) | 672.0 |
| Crystal size/mm$^3$ | 0.15 × 0.12 × 0.1 |
| Radiation | Cu Kα (λ = 1.54184) |
| 2Θ range for data collection/° | 7.898 to 145.686 |
| Index ranges | $-3 \le h \le 5$, $-14 \le k \le 15$, $-32 \le l \le 32$ |
| Reflections collected | 12635 |
| Independent reflections | 2947 [$R_{int}$ = 0.0459, $R_{sigma}$ = 0.0326] |
| Data/restraints/parameters | 2947/0/218 |
| Goodness-of-fit on F$^2$ | 1.043 |
| Final R indexes [I>=2σ (I)] | $R_1$ = 0.0472, $wR_2$ = 0.1305 |
| Final R indexes [all data] | $R_1$ = 0.0510, $wR_2$ = 0.1333 |
| Largest diff. peak/hole / e Å$^{-3}$ | 0.63/-0.18 |
| Flack parameter | 0.01(15) |

# References


[1] Zhang, N.; Zhang, C.; Hu, X.; Xie, X.; Liu, Y. Nickel-Catalyzed C(sp$^3$)-H Functionalization of Benzyl Nitriles: Direct Michael Addition to Terminal Vinyl Ketones. *Org. Lett.* **2021**, *23*, 6004–6009.

[2] Chen, X.; He, Q.; Xie, Y.; Yang, C. Palladium(II)-Catalyzed Synthesis of Functionalized Indenones via Oxidation and Cyclization of 2-(2-Arylethynylphenyl)acetonitriles. *Org. Biomol. Chem.* **2013**, *11*, 2582–2585.

[3] Chen, Z.; Nie, B.; Li, X.; Liu, T.; Li, C.; Huang, J. Ligand-controlled Regiodivergent Ni-Catalyzed trans-Hydroboration/Carboboration of Internal Alkynes with $B_2pin_2$. *Chem. Sci.* **2024**, *15*, 2236–2242.

[4] Chen, L.L.; Zhang, J.W.; Yang, W.W.; Fu, J.Y.; Zhu, J.Y.; Wang, Y.B. Synthesis of 1-Cyano-3-acylnaphthalenes via Formal [4+2] Benzannulation of 2-(2-Alkynylphenyl)acetonitriles and Alkynones. *J. Org. Chem.* **2019**, *84*, 8090–8099.

[5] Lin, H.S.; Pan, Y.Z.; Tian, Y.H.; Pan, Y.M.; Wang, X. Palladium-Catalyzed Tandem Cyclization of 2-(2 Ethynylphenyl)acetonitriles and Isocyanides: Access to Indeno[2,1-b]pyrroles. *Adv. Synth. Catal.* **2022**, *364*, 1117–1121.

[6] Sedelmeier, J.; Ley, S.V.; Lange, H.; Baxendale, I.R. Pd-EnCat™ TPP30 as a Catalyst for the Generation of Highly Functionalized Aryl- and Alkenyl-Substituted Acetylenes via Microwave-Assisted Sonogashira Type Reactions. *Eur. J. Org. Chem.* **2009**, 26, 4412–4420.

[7] Chen, W.; Liu, T.; Li, S.; Li, G.; Wu, G.; Gao, Y.; Xu, Z.; Wu, Y.; Peng, X.; Huang, J. Redox-Neutral Nickel-Catalyzed Selective Hydroalkynylation of Internal Alkyne and Its Application in Anticancer Agent Discovery. *Chin. J. Chem.* **2024**, *42*, 3317–3323.

## NMR Spectra for all compounds

### $^1$H NMR (400 MHz, Chloroform-*d*) of compound 1w

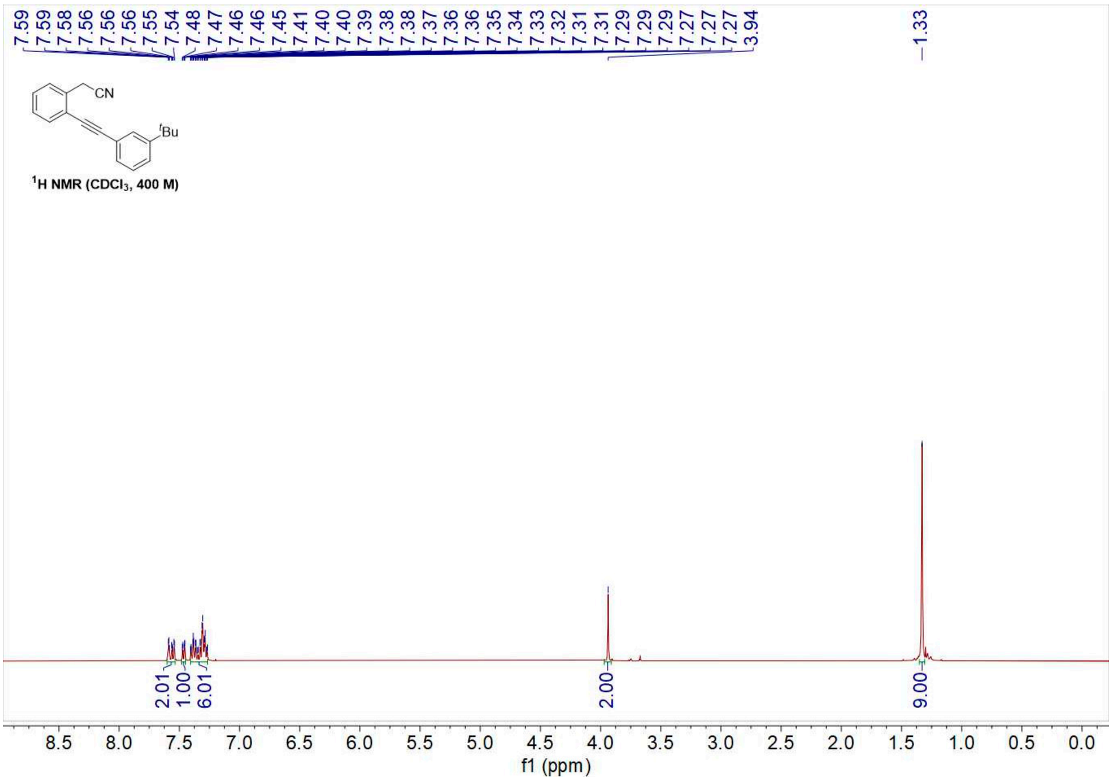

### $^{13}$C NMR (101 MHz, Chloroform-*d*) of compound 1w

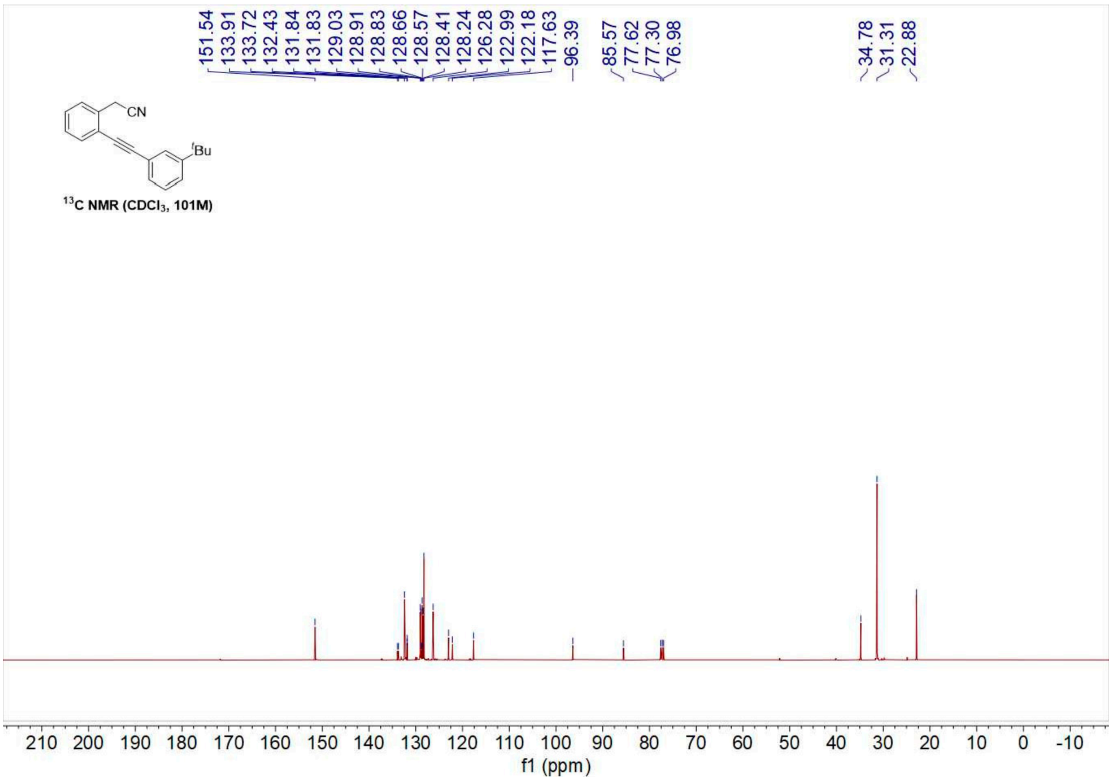

**$^{1}$H NMR (400 MHz, Chloroform-*d*) of compound 1x**

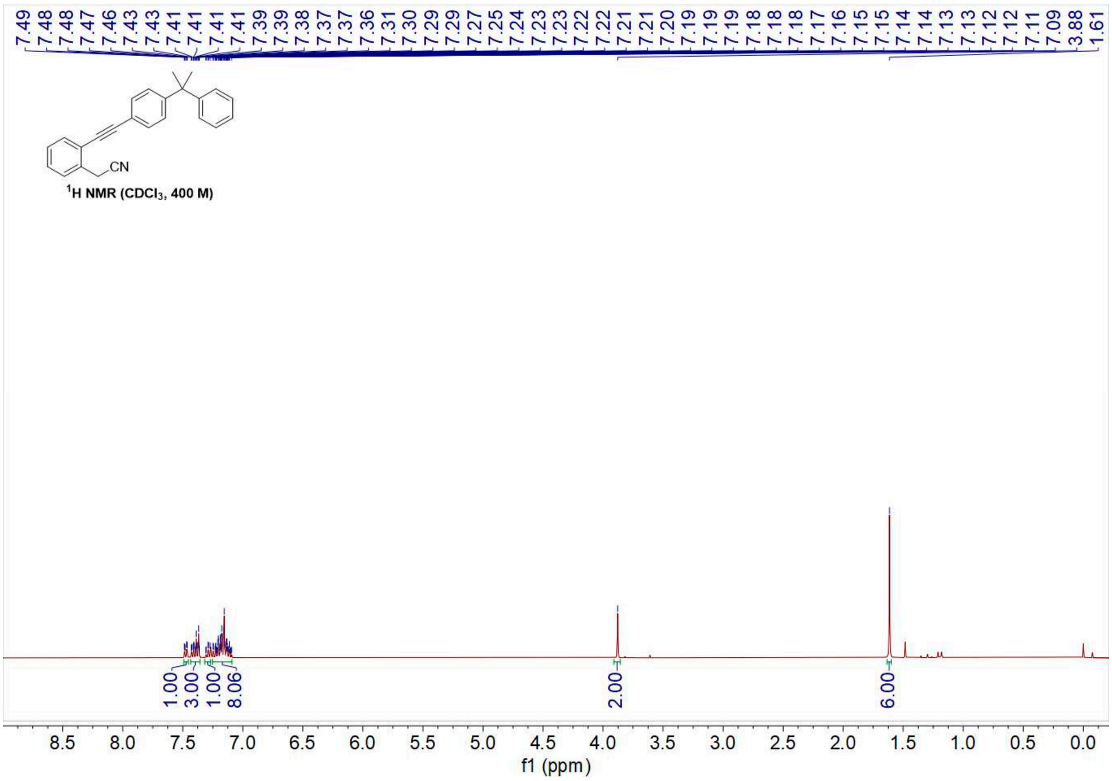

**$^{13}$C NMR (101 MHz, Chloroform-*d*) of compound 1x**

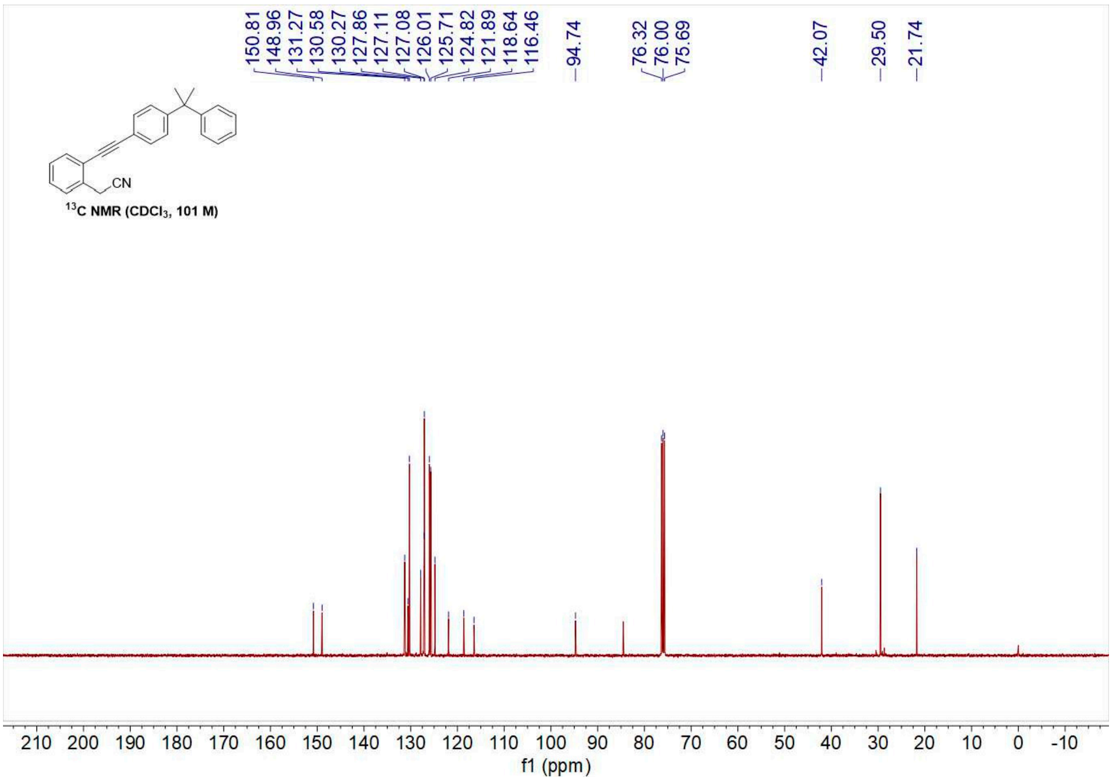

## $^{1}$H NMR (400 MHz, Chloroform-*d*) of compound 1y

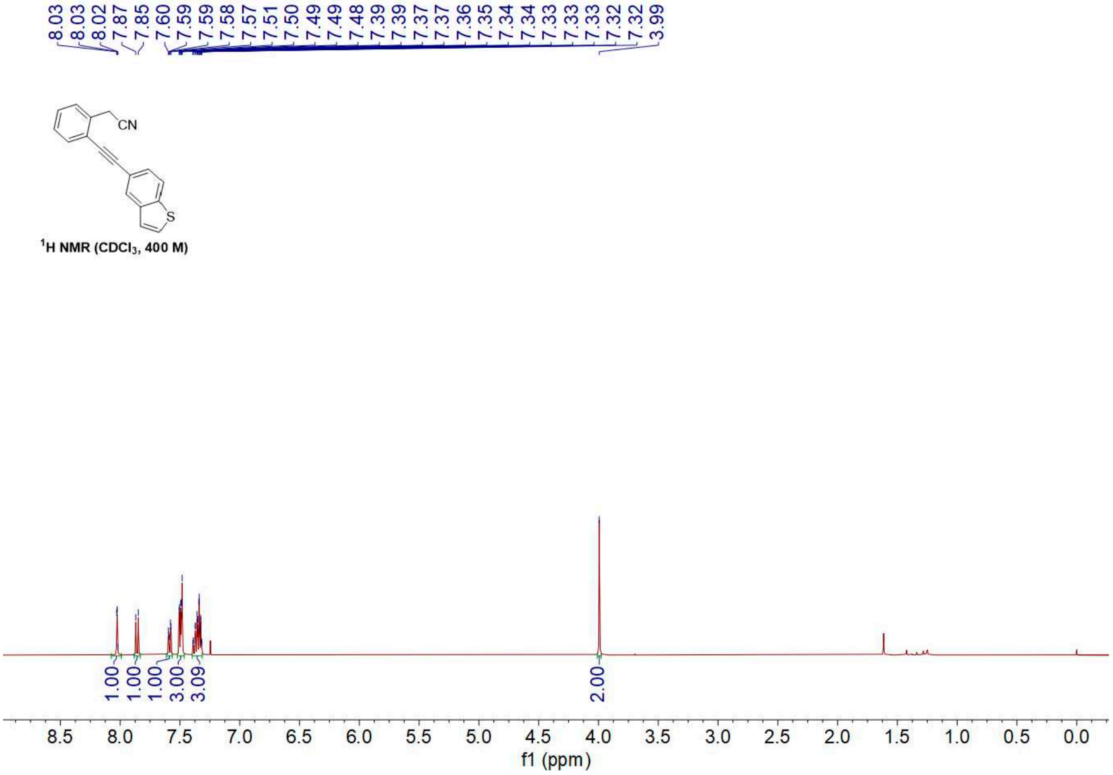


## $^{13}$C NMR (101 MHz, Chloroform-*d*) of compound 1y

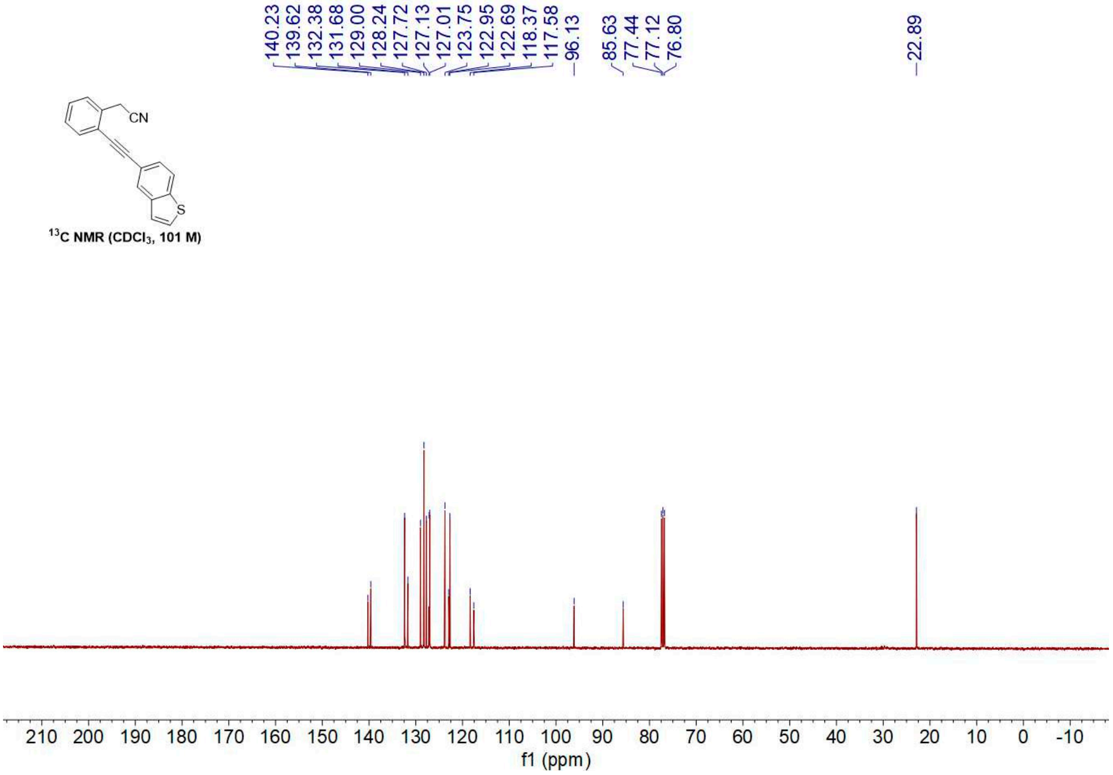

$^{1}$H NMR (400 MHz, Chloroform-*d*) of compound 3a

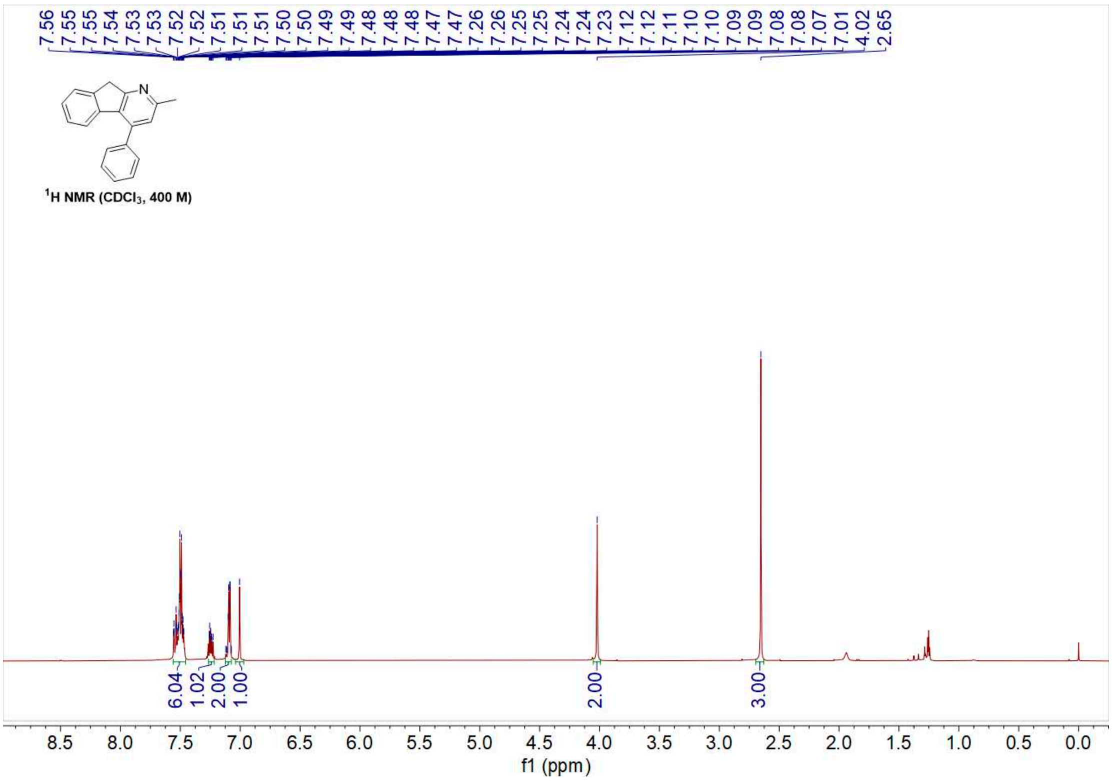


$^{13}$C NMR (101 MHz, Chloroform-*d*) of compound 3a

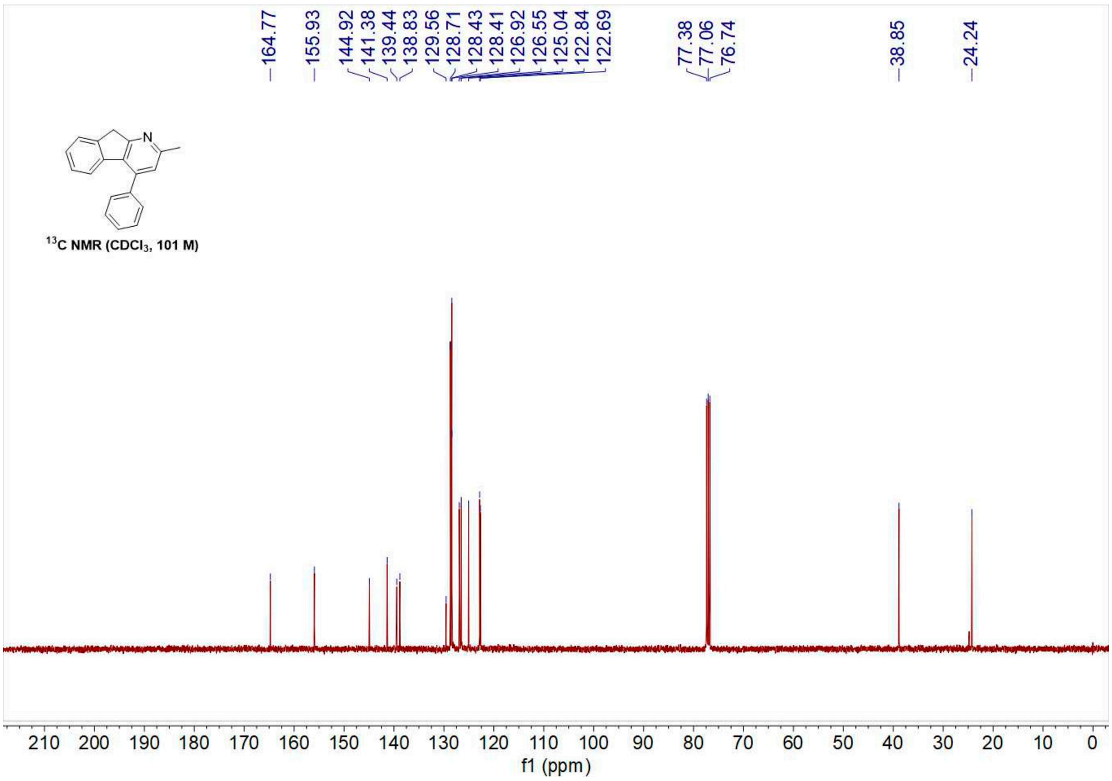

## $^{1}H$ NMR (400 MHz, Chloroform-*d*) of compound 3b

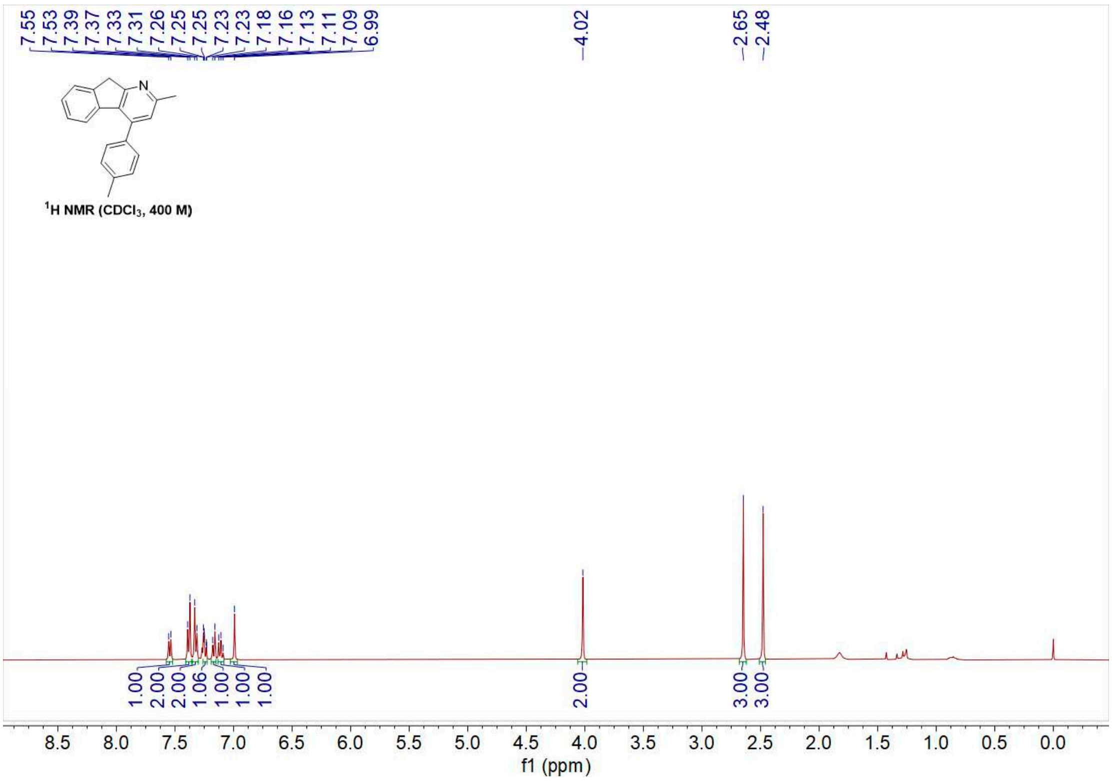


## $^{13}C$ NMR (101 MHz, Chloroform-*d*) of compound 3b

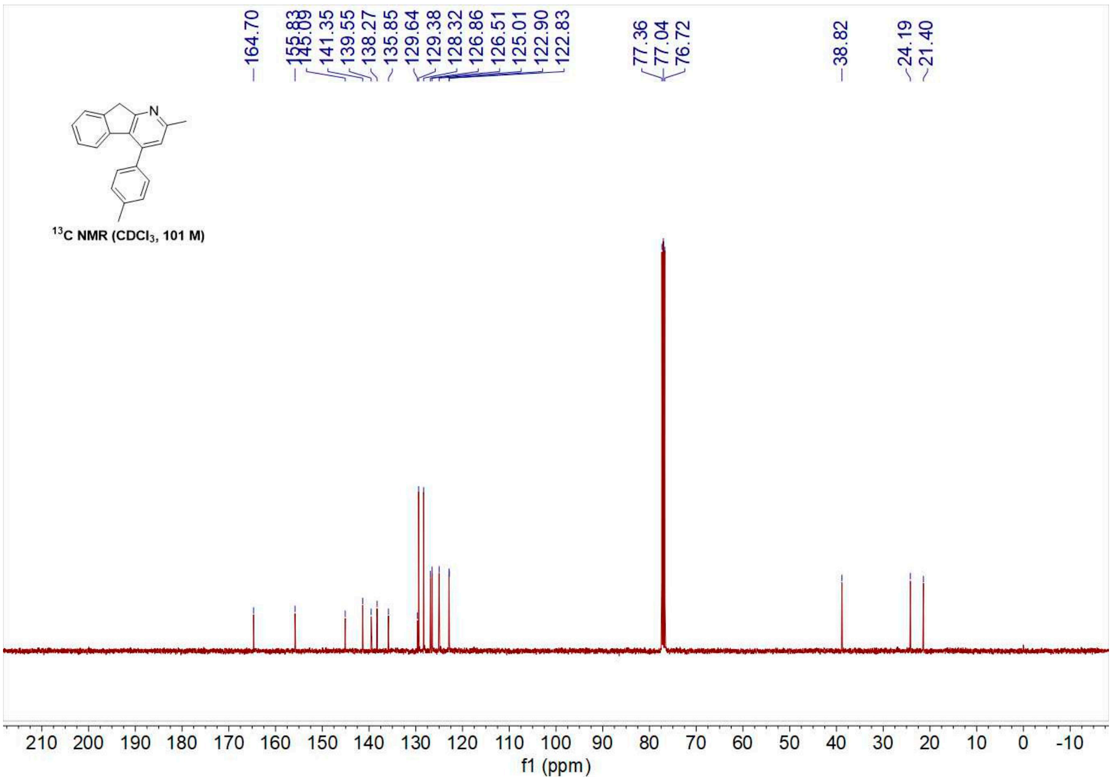

**$^{1}$H NMR (400 MHz, Chloroform-*d*) of compound 3c**

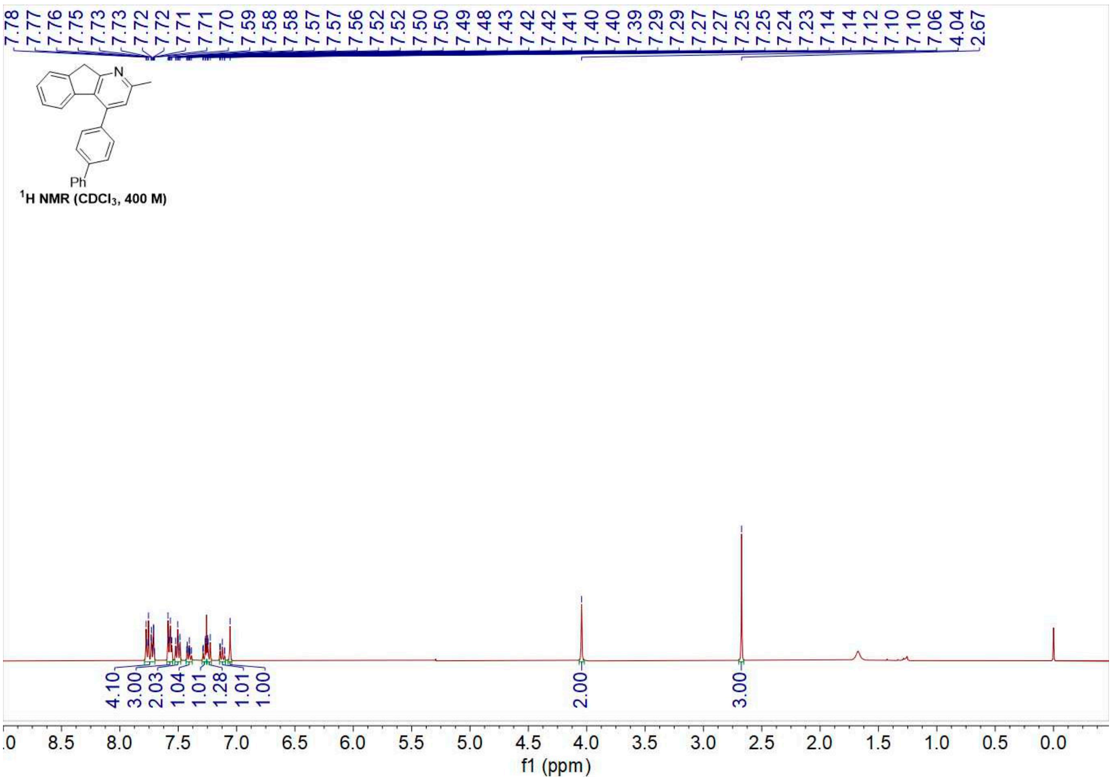


**$^{13}$C NMR (101 MHz, Chloroform-*d*) of compound 3c**

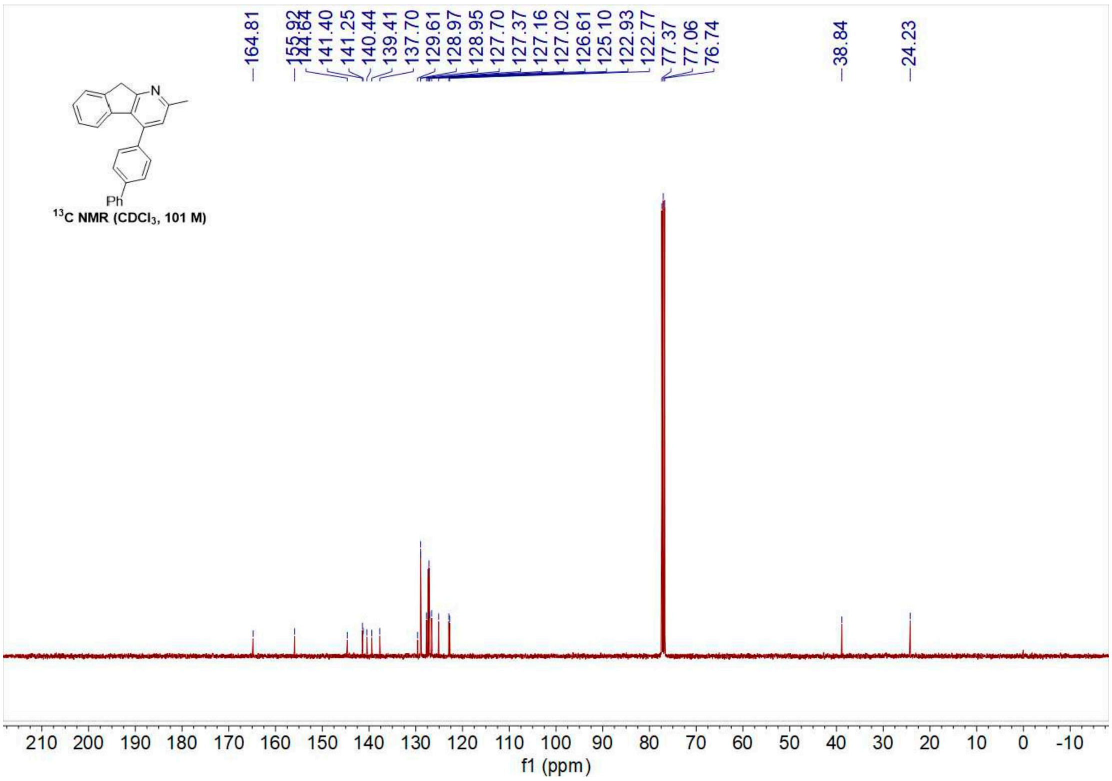

## $^{1}$H NMR (400 MHz, Chloroform-*d*) of compound 3d

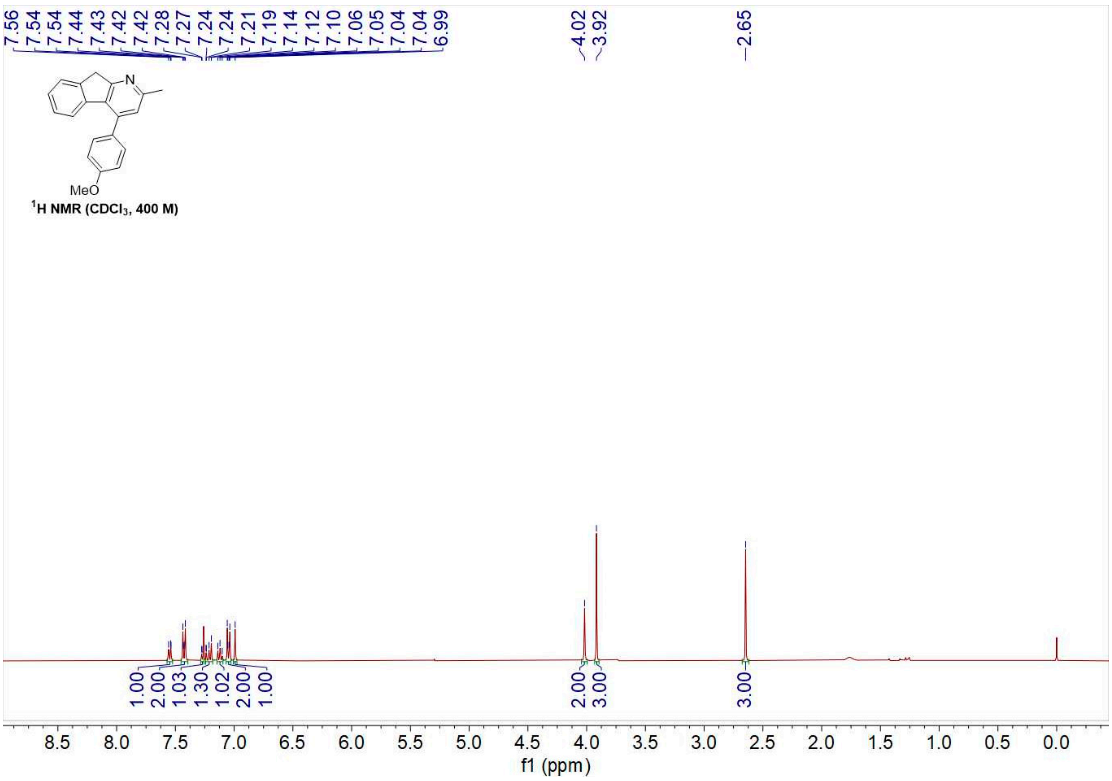


## $^{13}$C NMR (101 MHz, Chloroform-*d*) of compound 3d

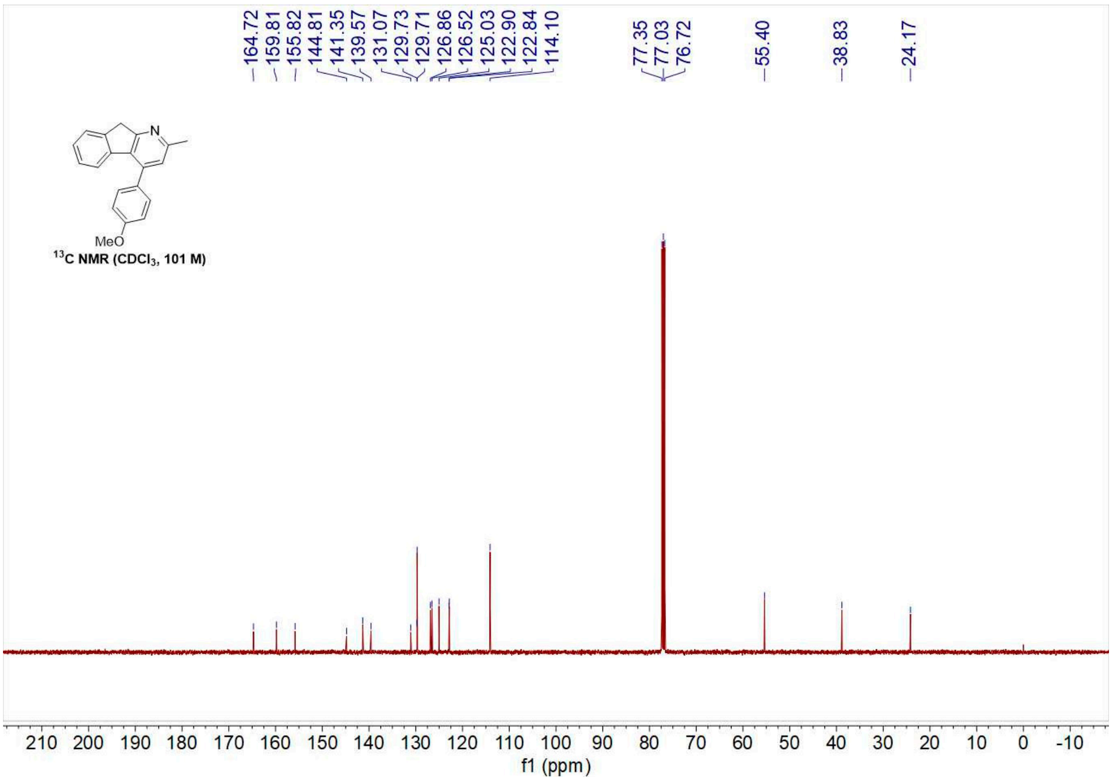

## $^1$H NMR (400 MHz, Chloroform-*d*) of compound 3e

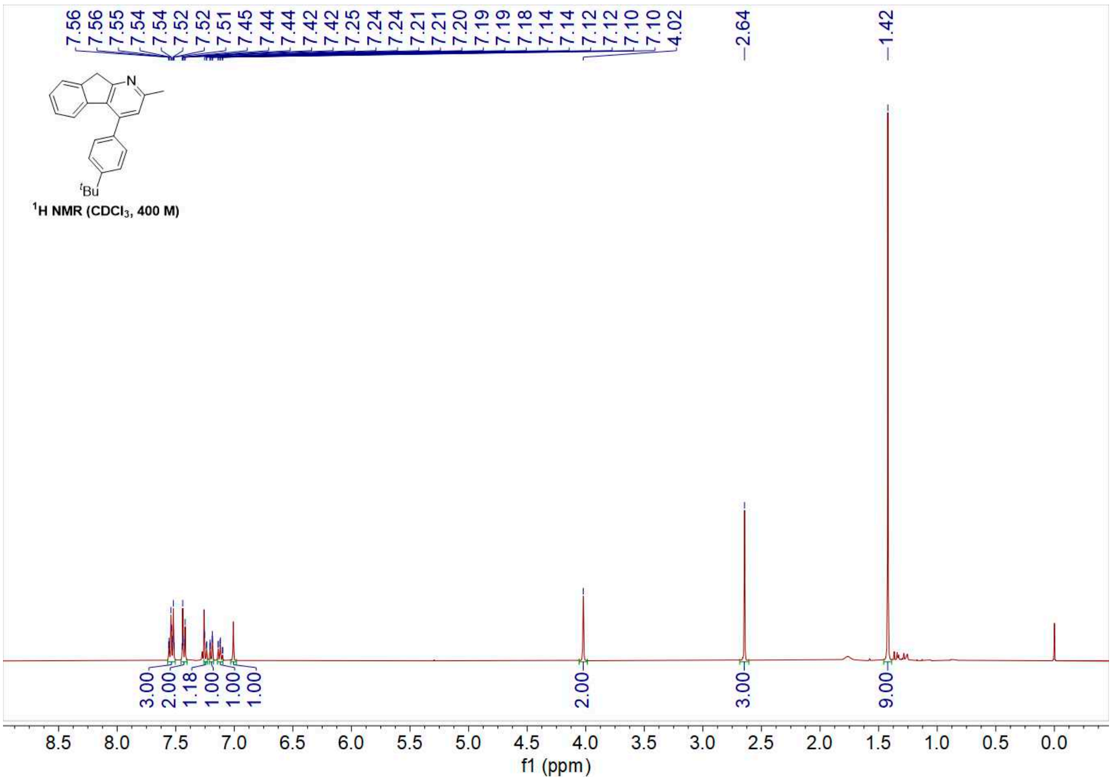


## $^{13}$C NMR (101 MHz, Chloroform-*d*) of compound 3e

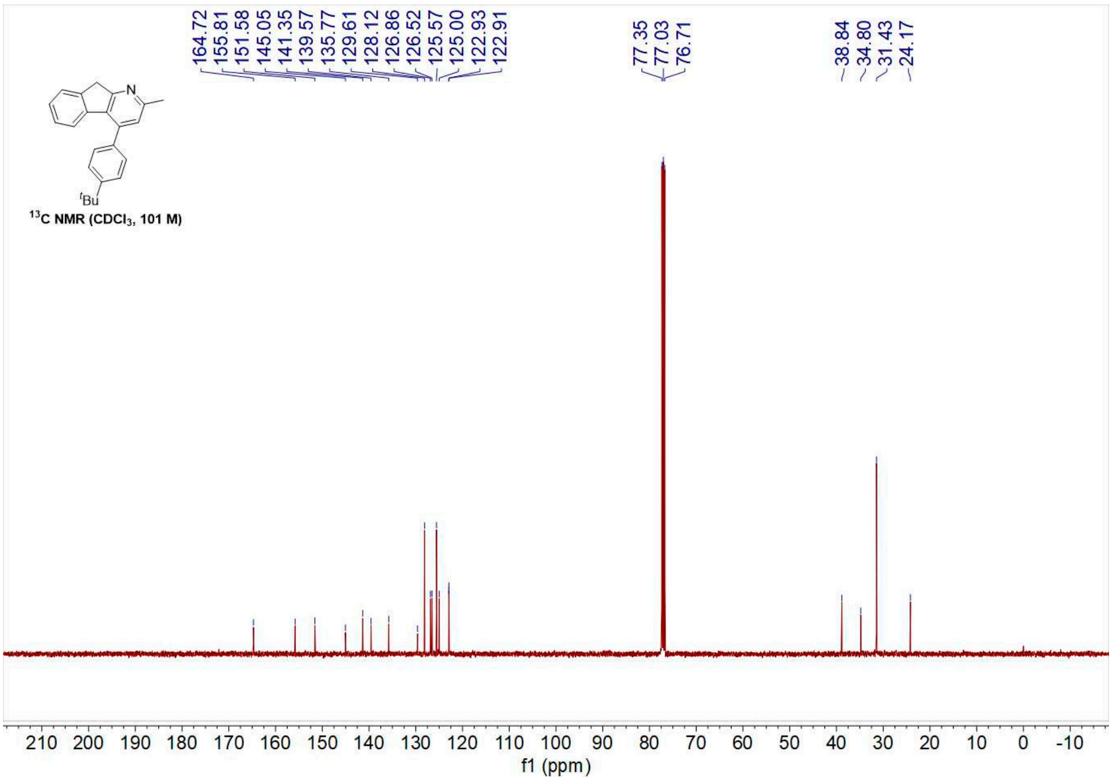

## $^{1}$H NMR (400 MHz, Chloroform-*d*) of compound 3f

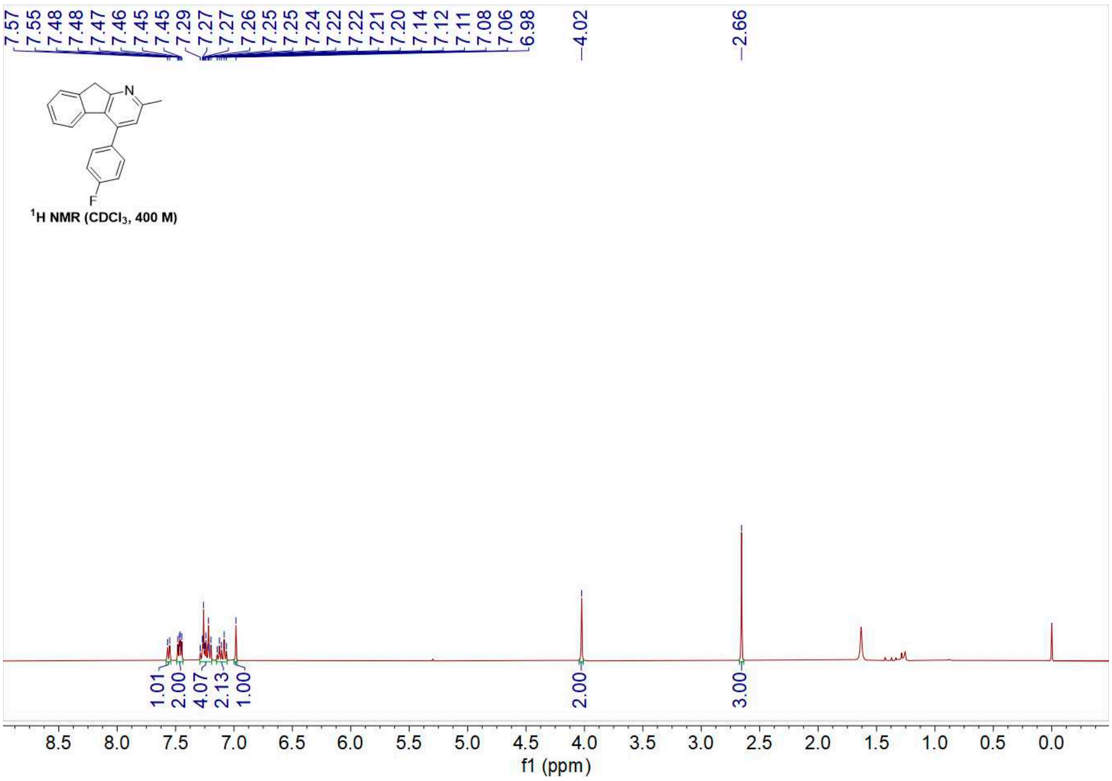


## $^{13}$C NMR (101 MHz, Chloroform-*d*) of compound 3f

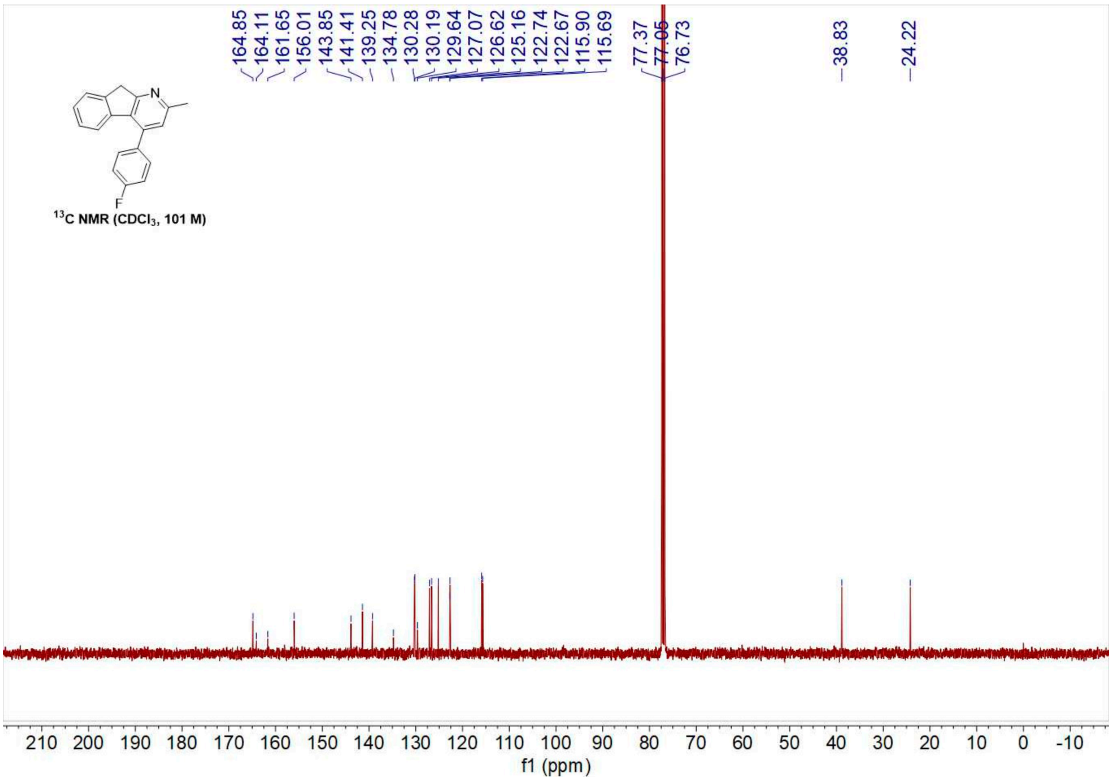

**$^{19}$F NMR (377 MHz, Chloroform-*d*) of compound 3f**

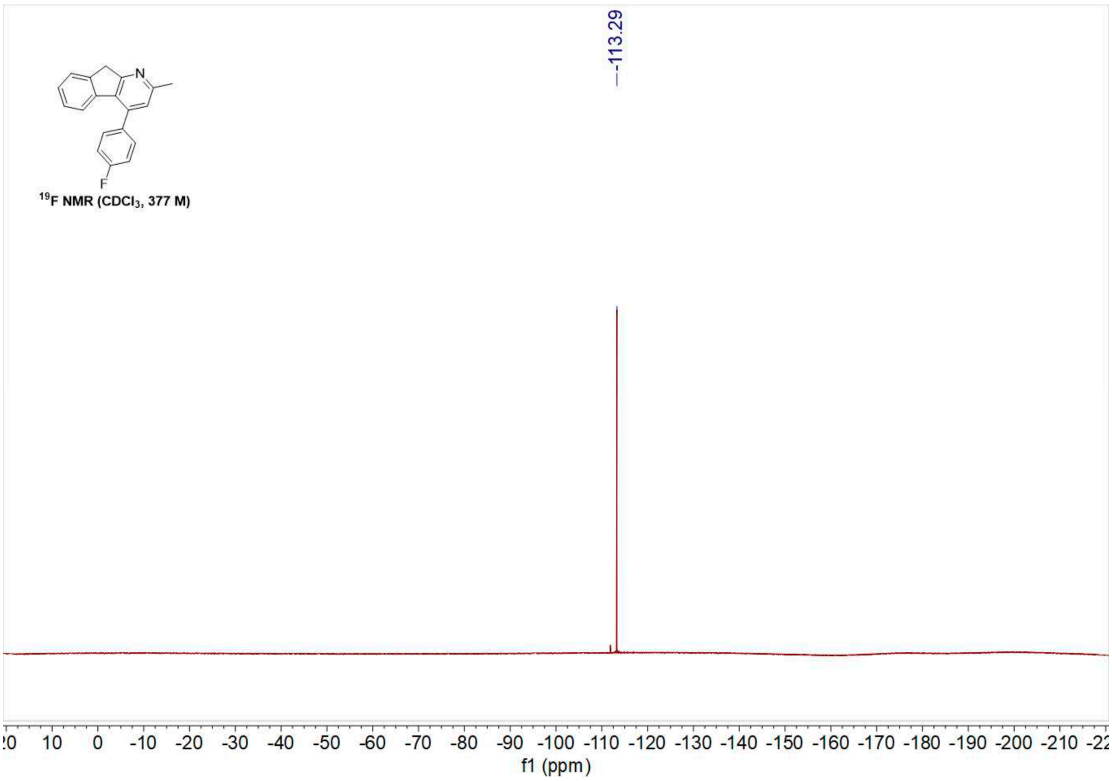


**$^{1}$H NMR (400 MHz, Chloroform-*d*) of compound 3g**

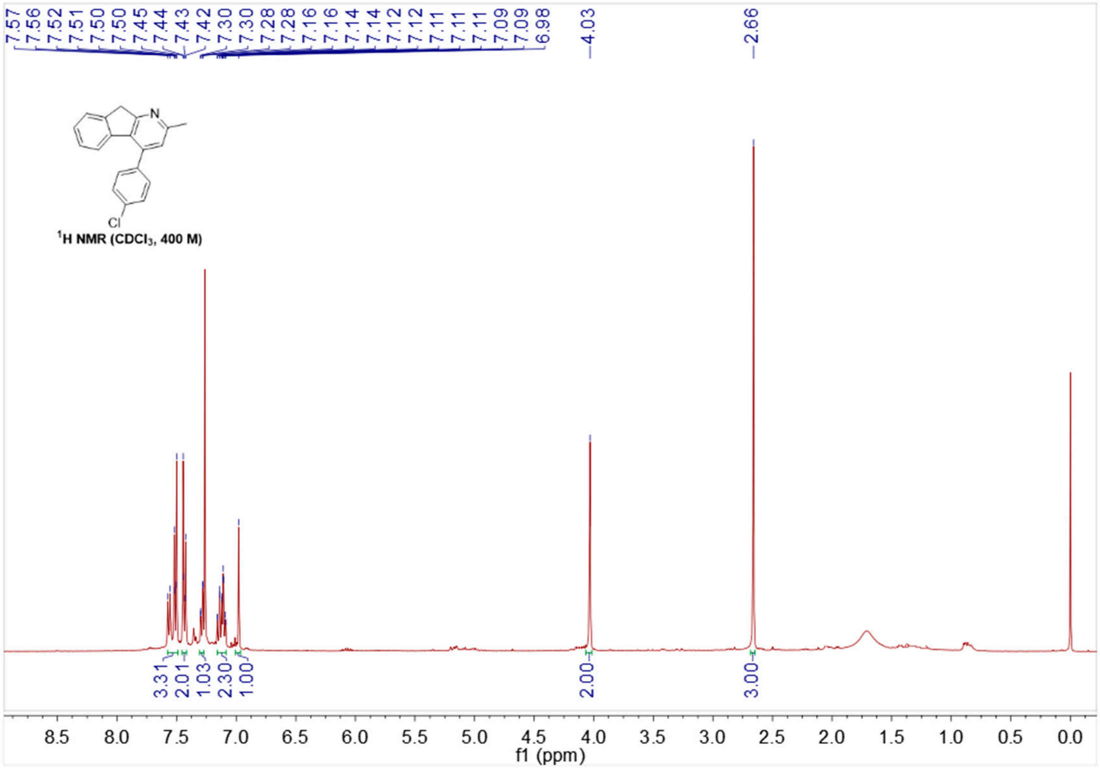

## $^{13}$C NMR (101 MHz, Chloroform-*d*) of compound 3g

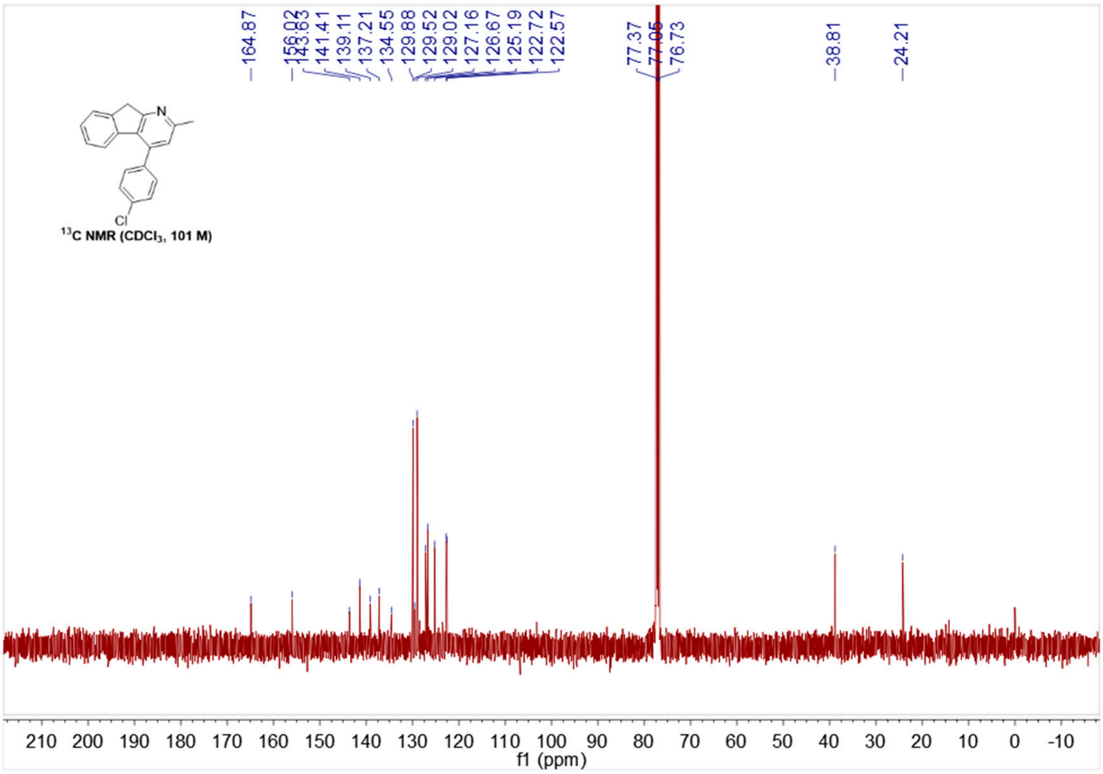


## $^{1}$H NMR (400 MHz, Chloroform-*d*) of compound 3h

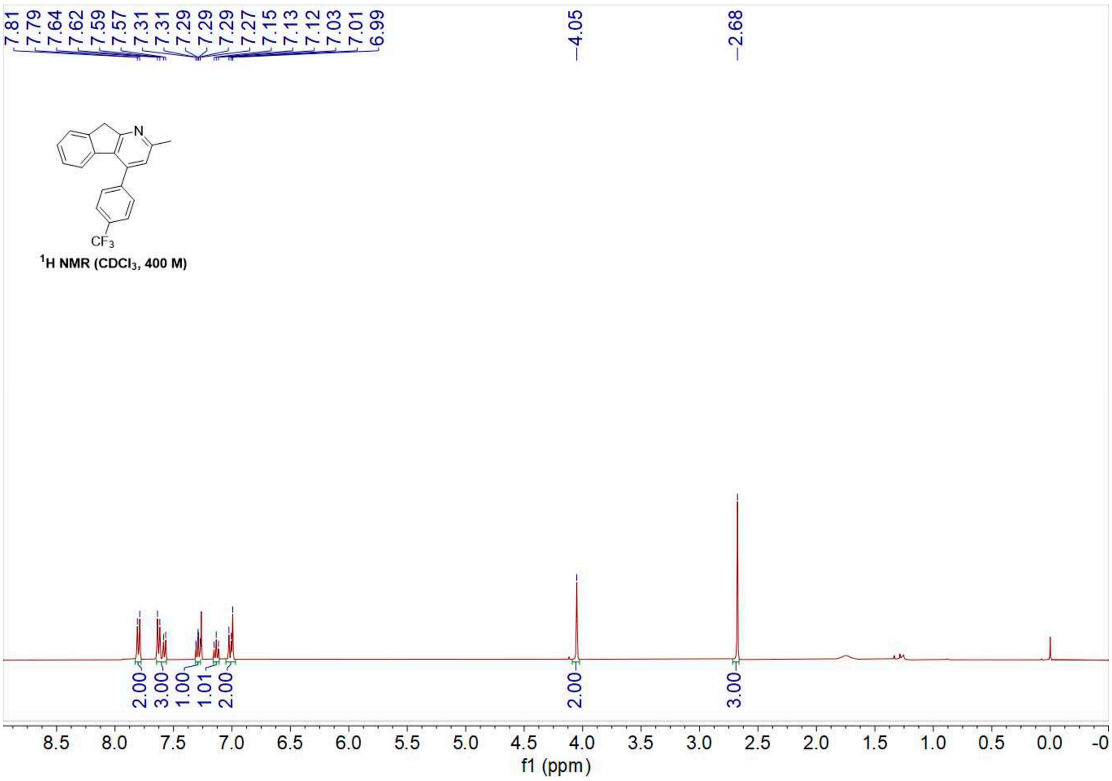

## $^{13}$C NMR (101 MHz, Chloroform-*d*) of compound 3h

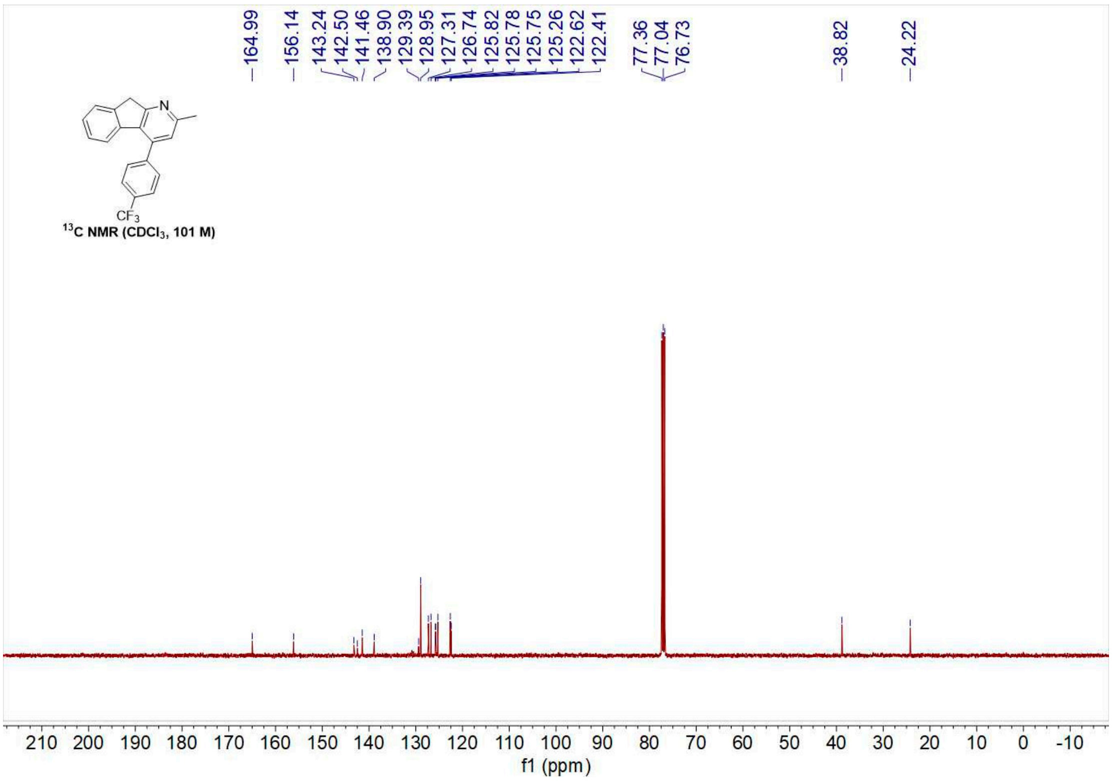


## $^{19}$F NMR (377 MHz, Chloroform-*d*) of compound 3h

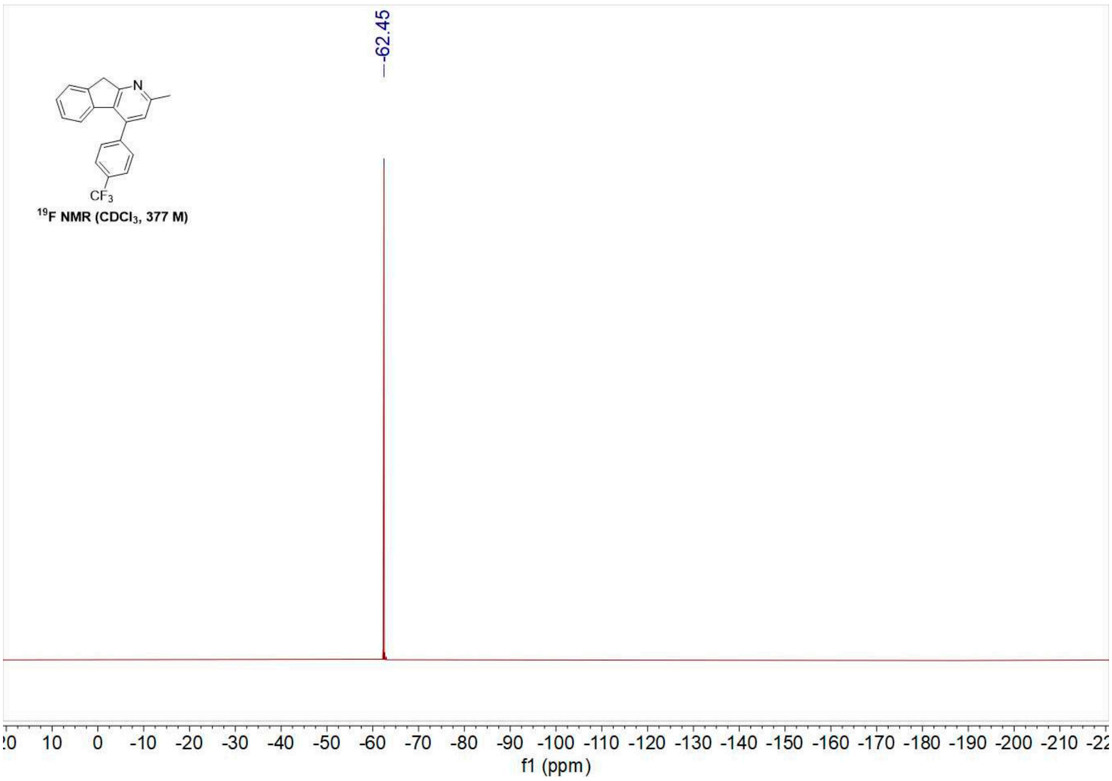

**$^1$H NMR (400 MHz, Chloroform-*d*) of compound 3i**

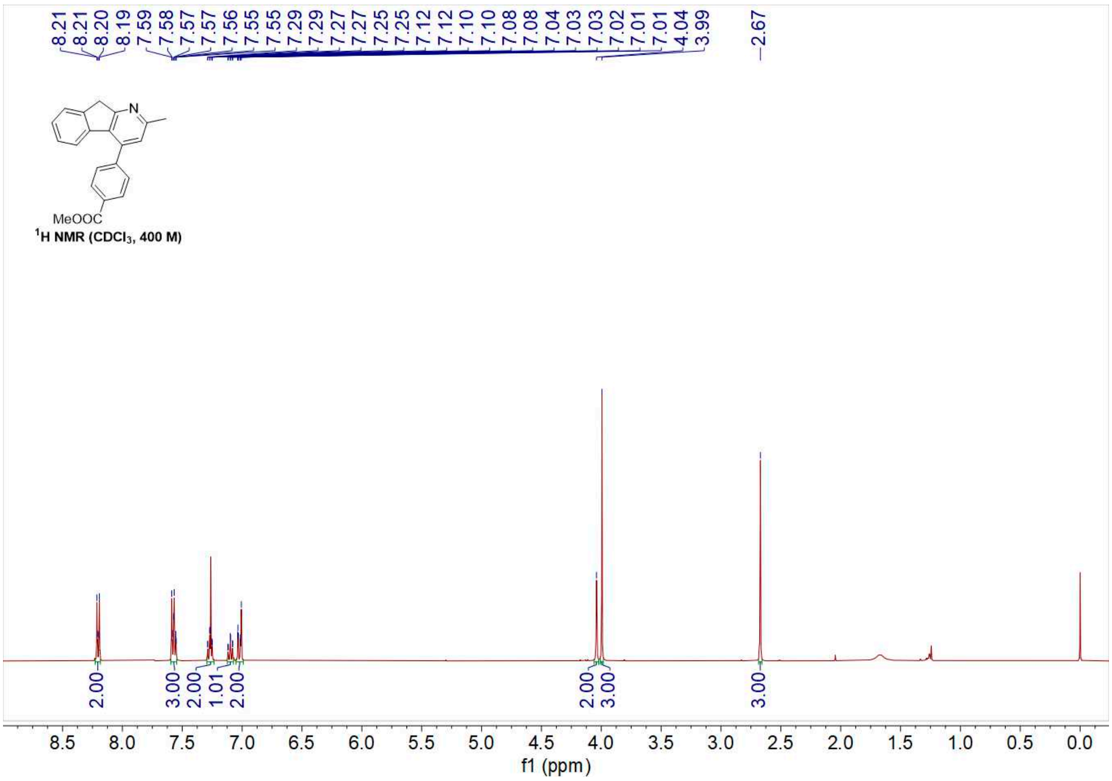

**$^{13}$C NMR (101 MHz, Chloroform-*d*) of compound 3i**

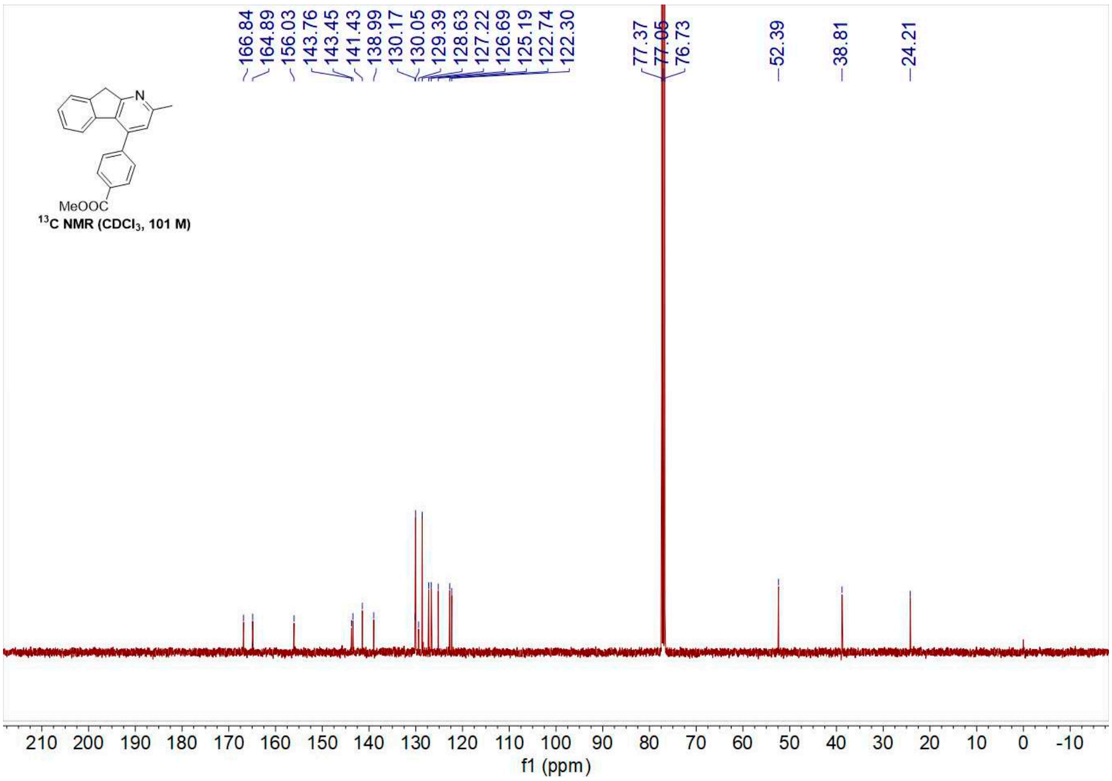

## $^{1}$H NMR (400 MHz, Chloroform-*d*) of compound 3j

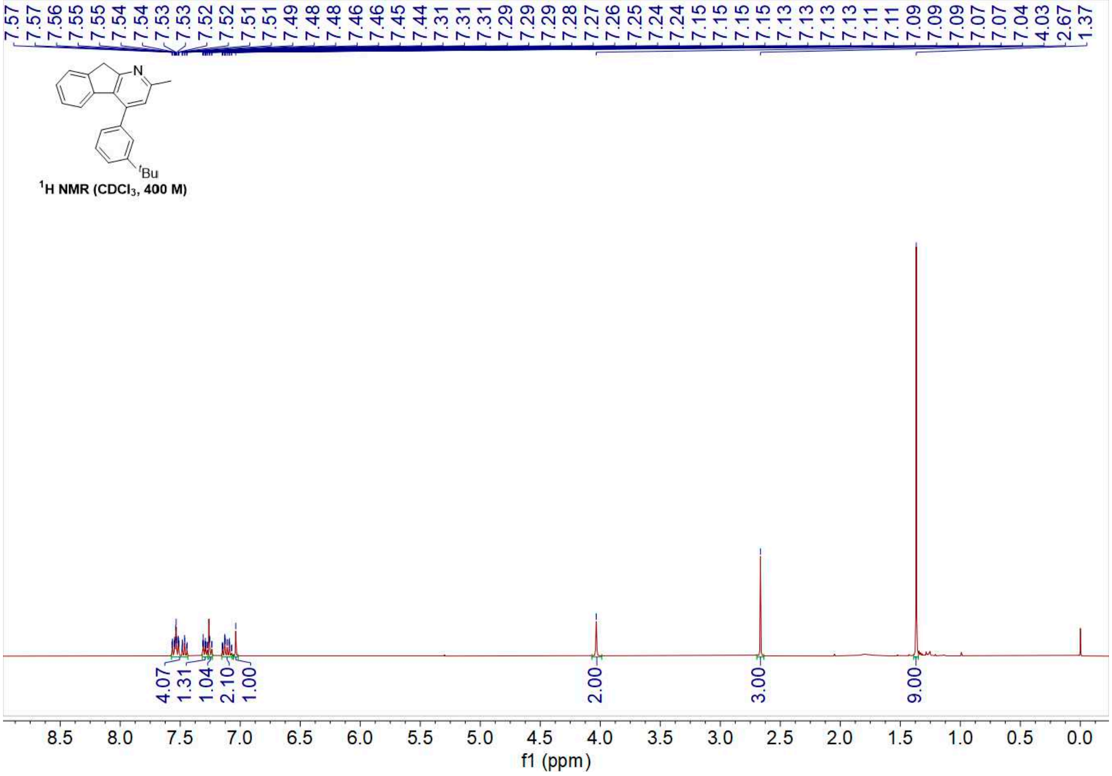

## $^{13}$C NMR (101 MHz, Chloroform-*d*) of compound 3j

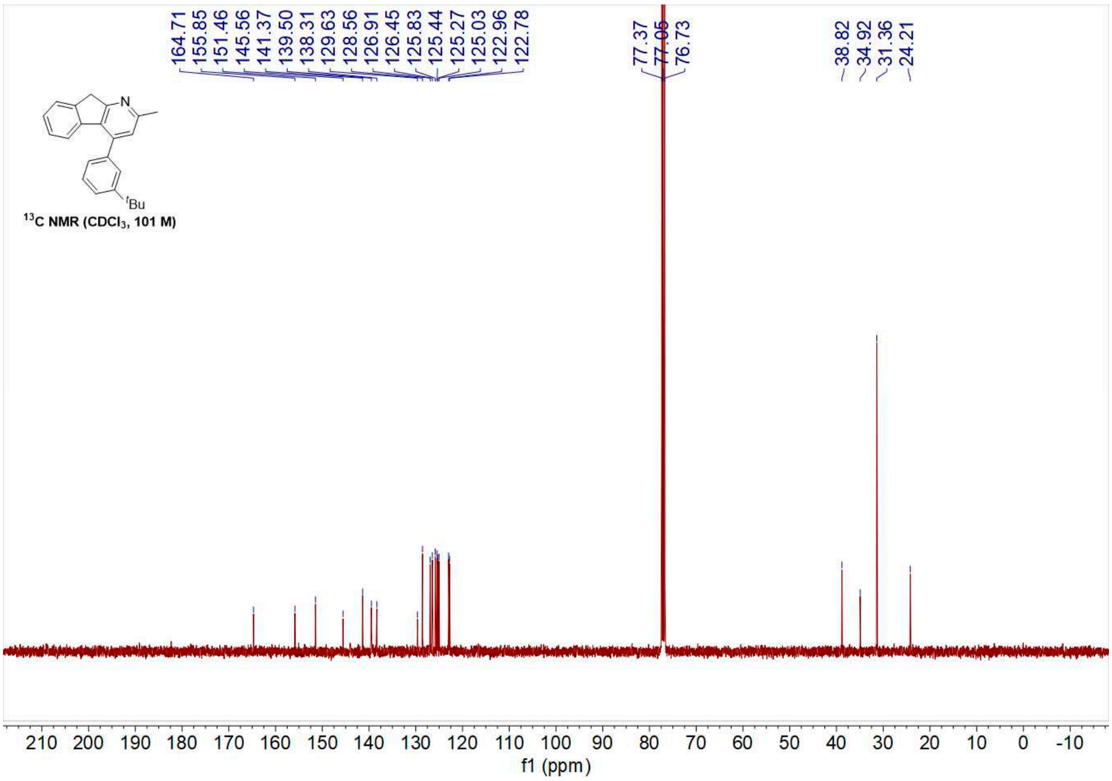

## $^1H$ NMR (400 MHz, Chloroform-*d*) of compound 3k

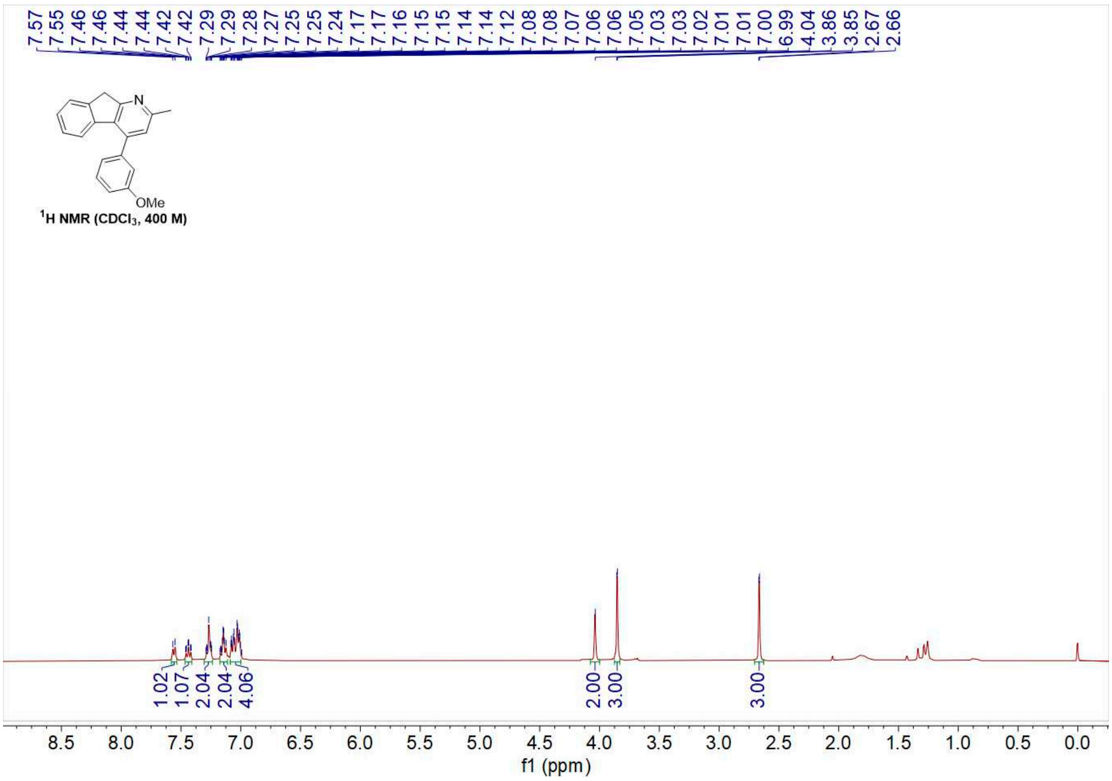


## $^{13}C$ NMR (101 MHz, Chloroform-*d*) of compound 3k

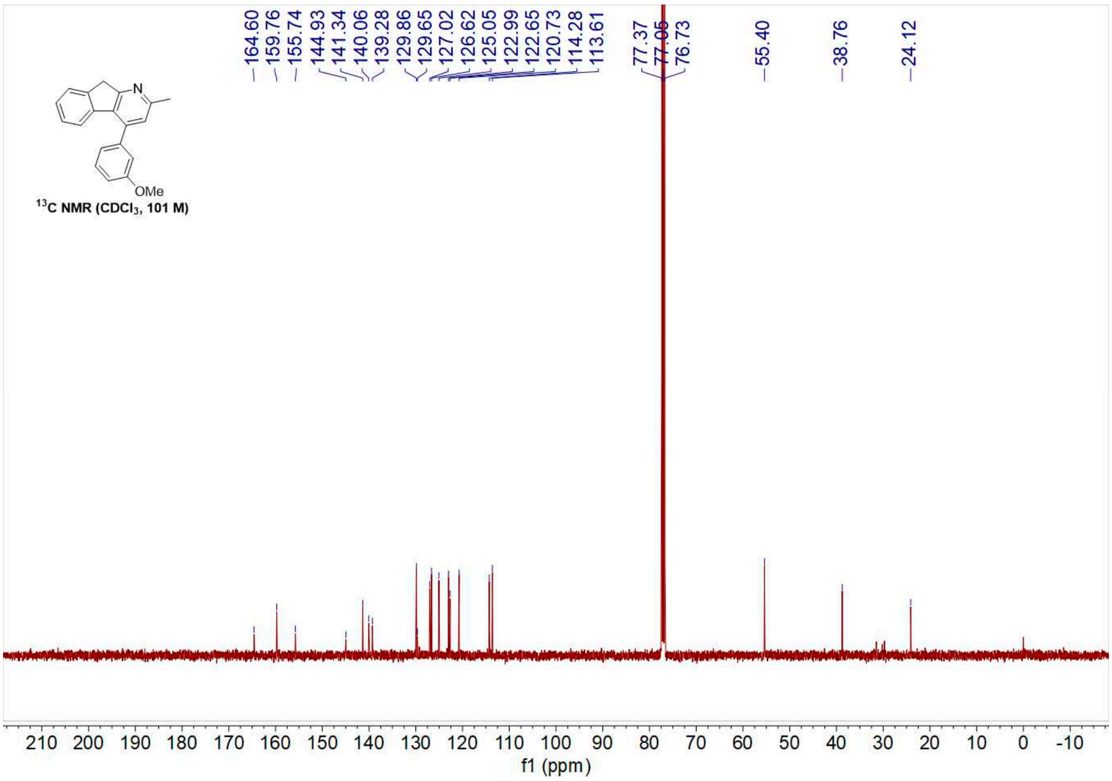

## $^{1}$H NMR (400 MHz, Chloroform-*d*) of compound 3l

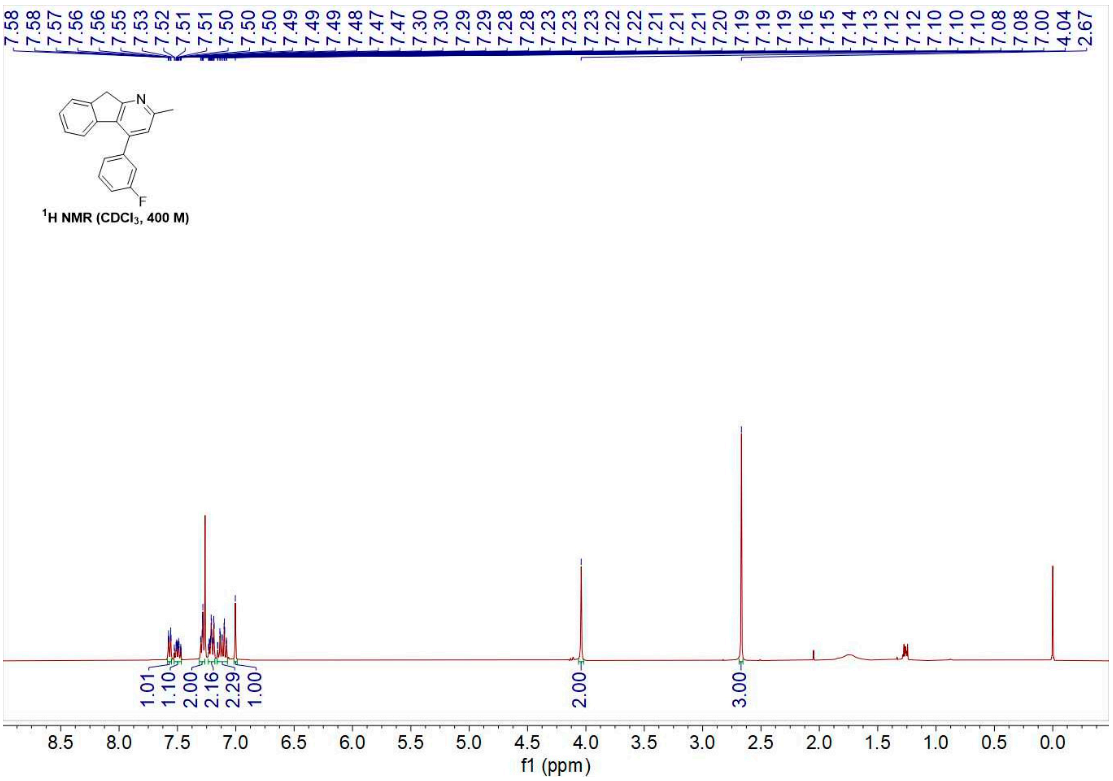

## $^{13}$C NMR (101 MHz, Chloroform-*d*) of compound 3l

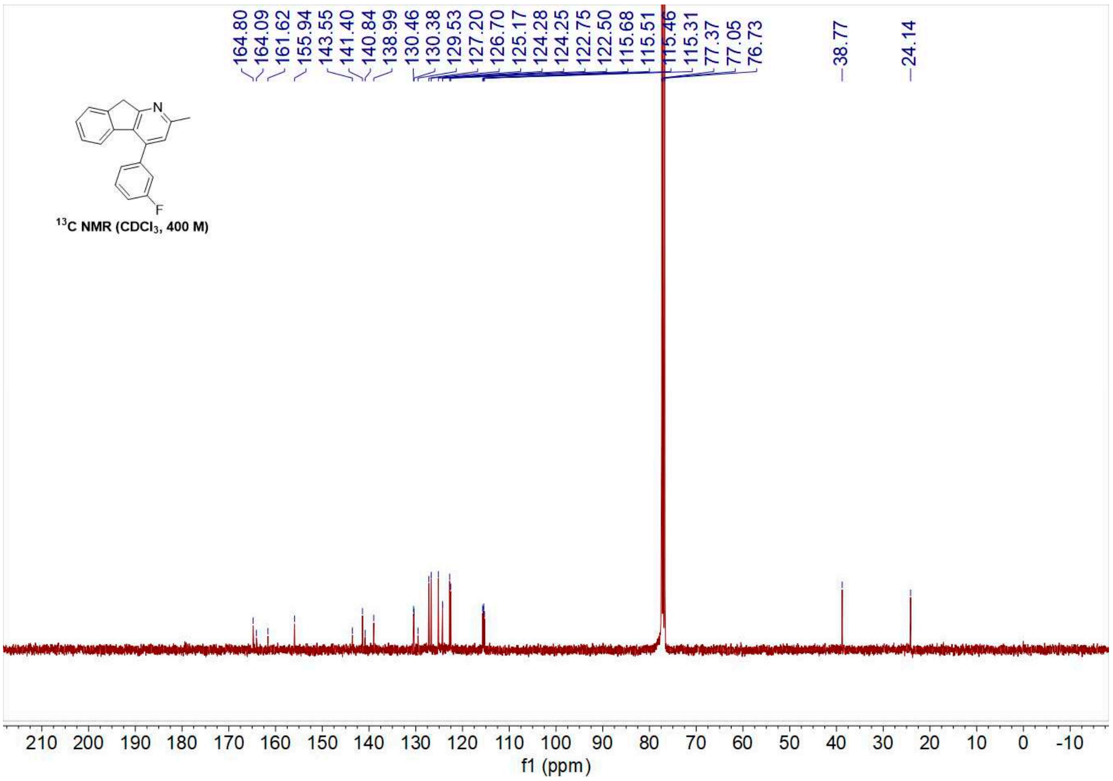

**$^{19}$F NMR (377 MHz, Chloroform-*d*) of compound 3l**

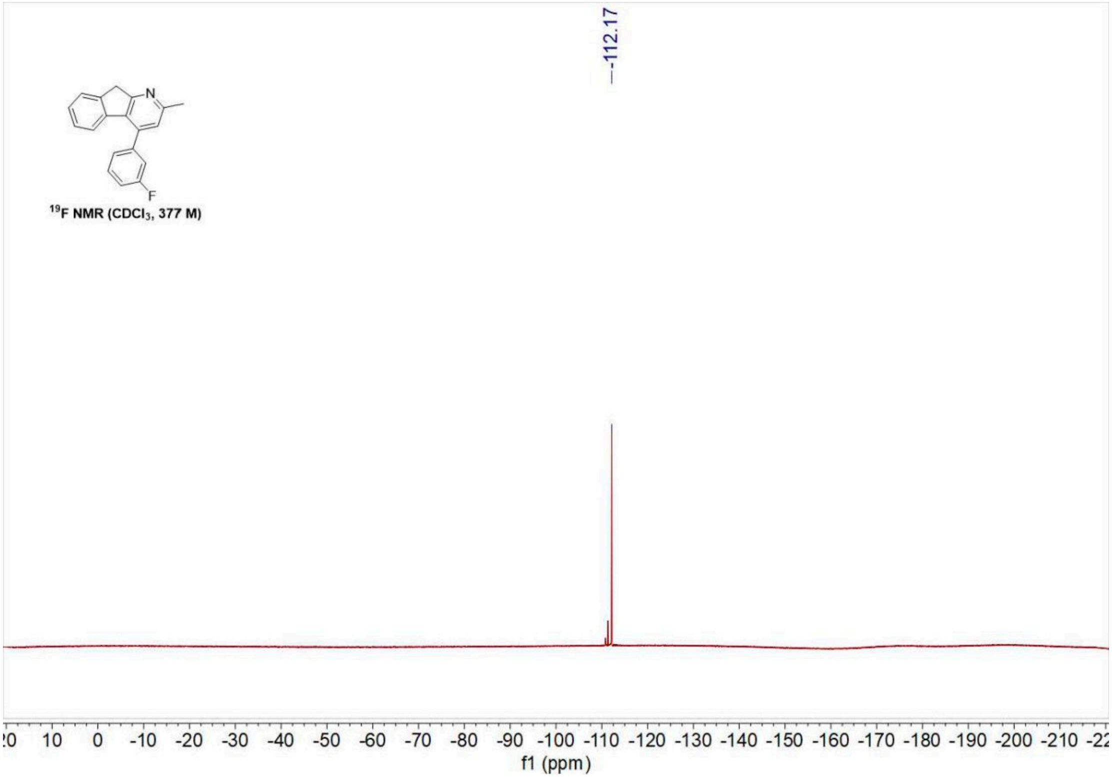

**$^{1}$H NMR (400 MHz, Chloroform-*d*) of compound 3m**

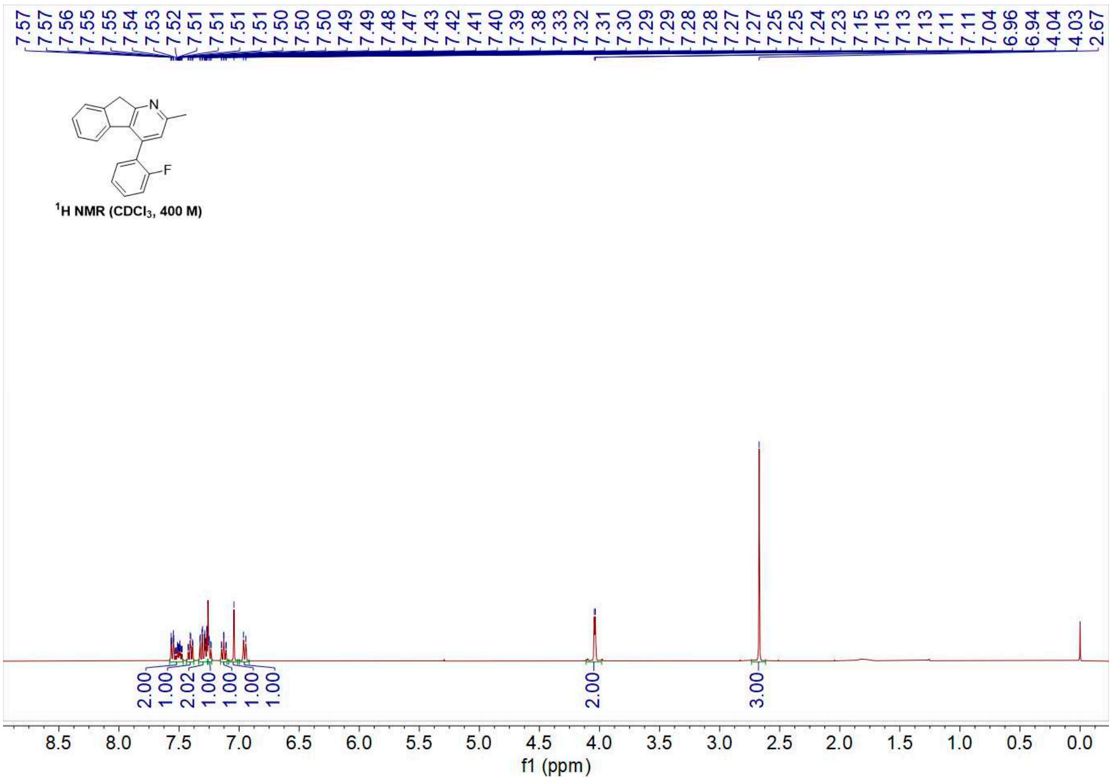

## $^{13}$C NMR (101 MHz, Chloroform-*d*) of compound 3m

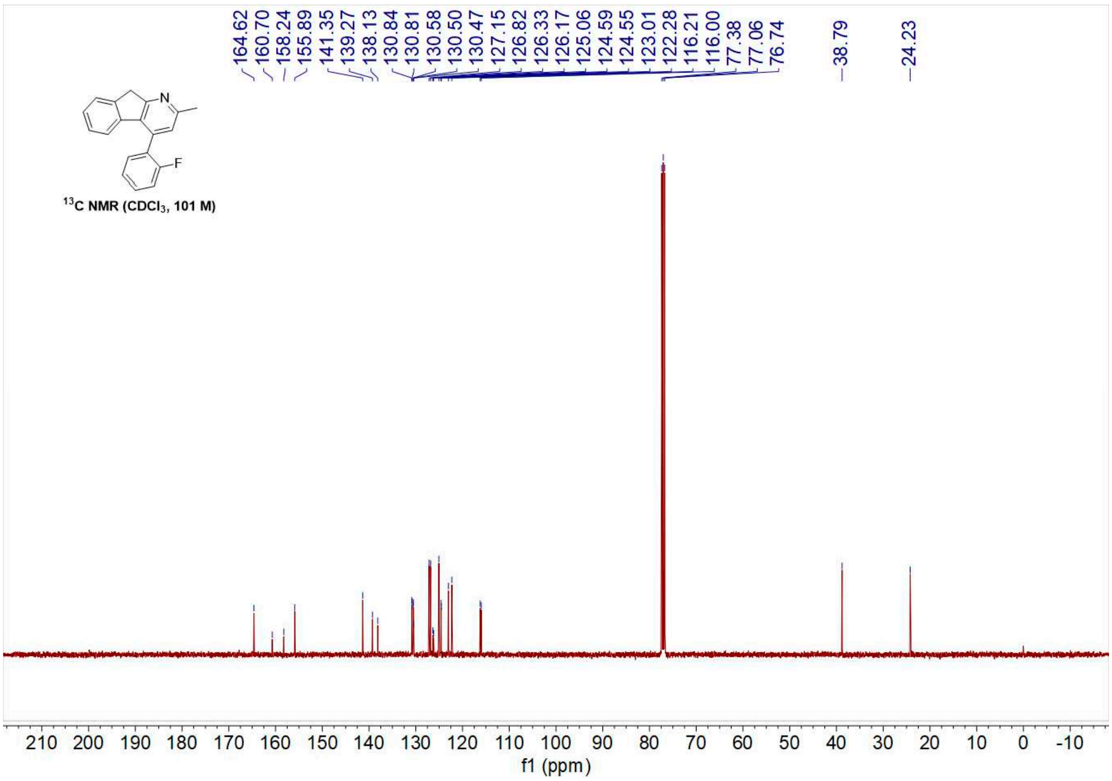


## $^{19}$F NMR (377 MHz, Chloroform-*d*) of compound 3m

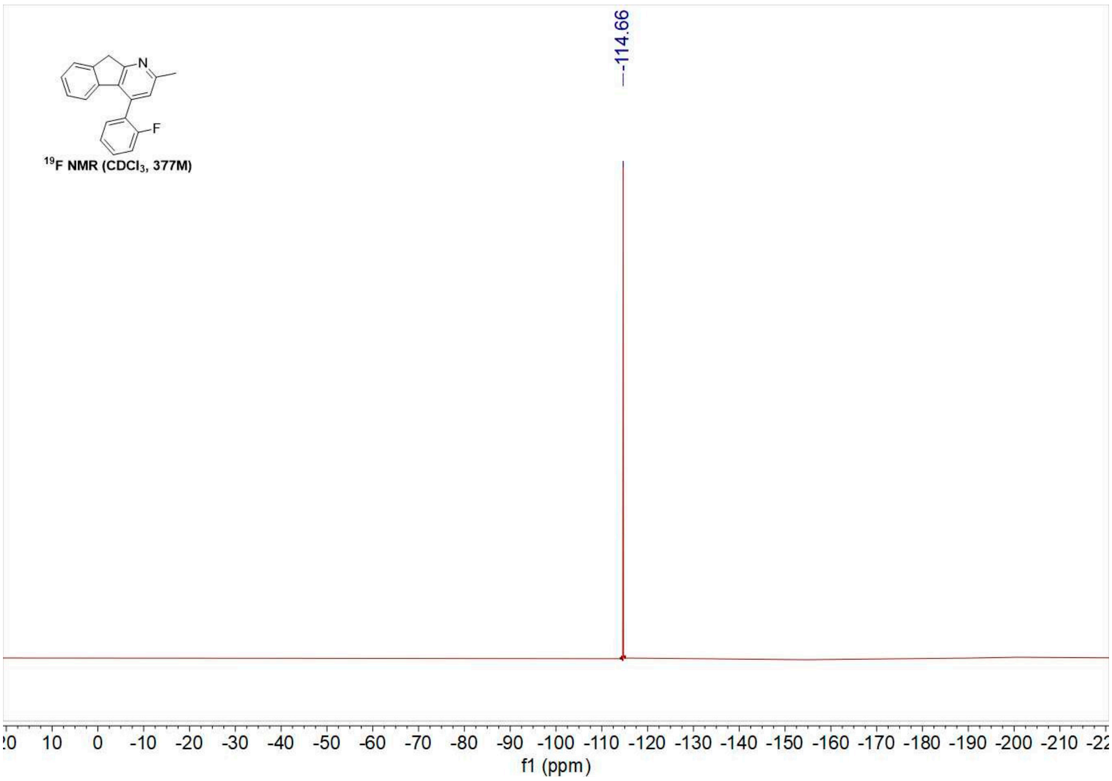

$^{1}$H NMR (400 MHz, Chloroform-*d*) of compound 3n

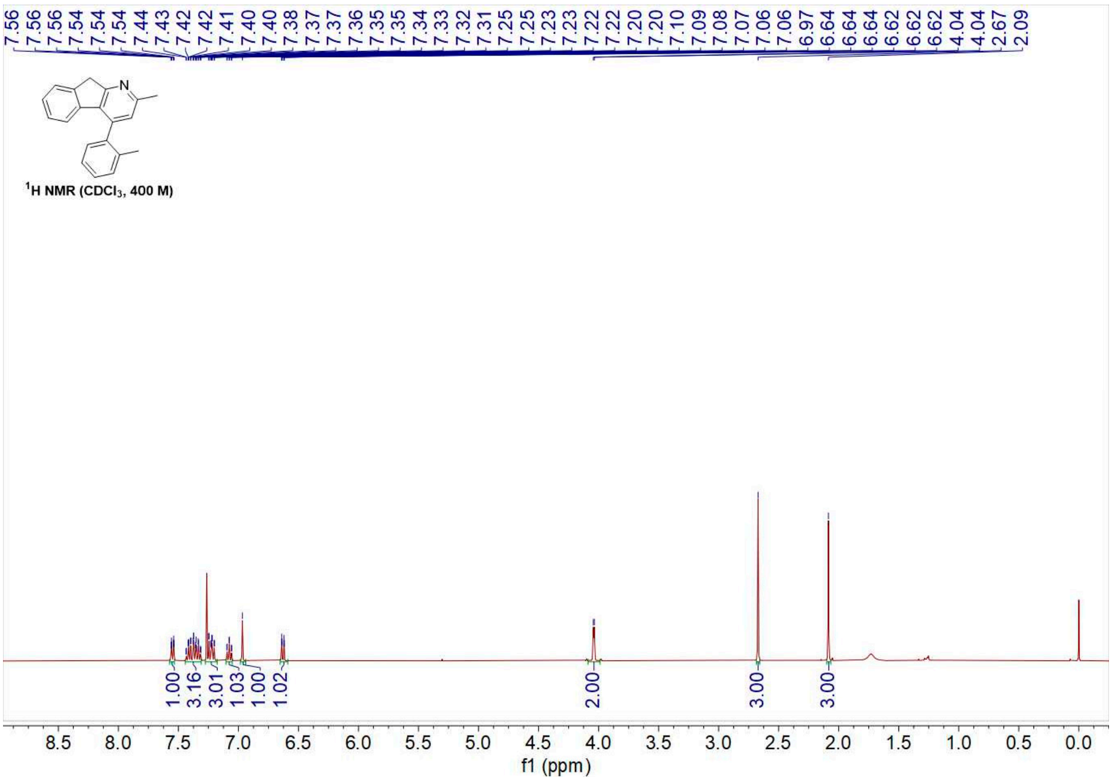

$^{13}$C NMR (101 MHz, Chloroform-*d*) of compound 3n

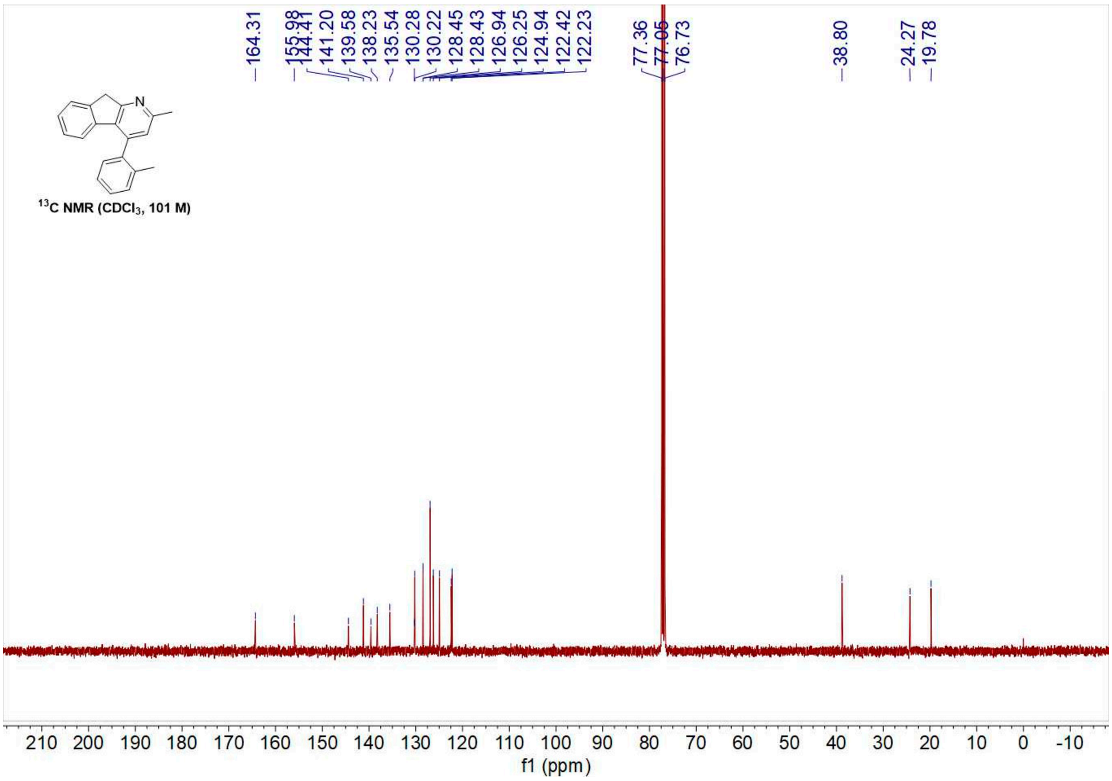

**$^{1}$H NMR (400 MHz, Chloroform-*d*) of compound 3o**

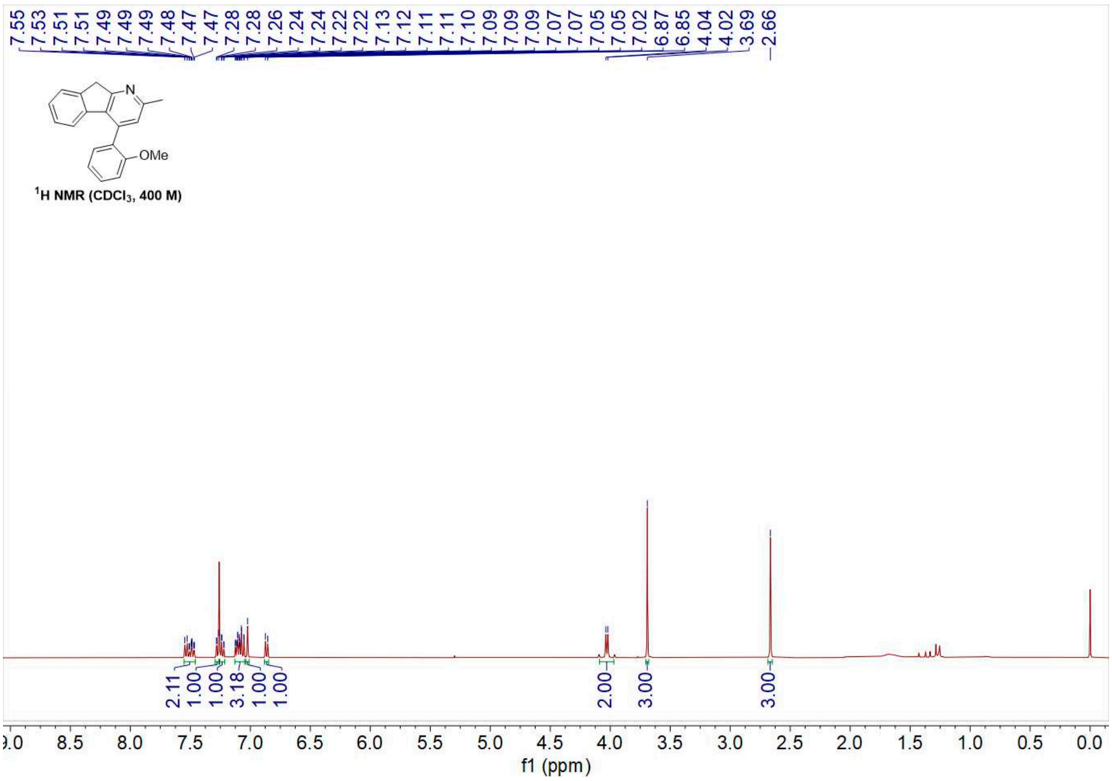


**$^{13}$C NMR (101 MHz, Chloroform-*d*) of compound 3o**

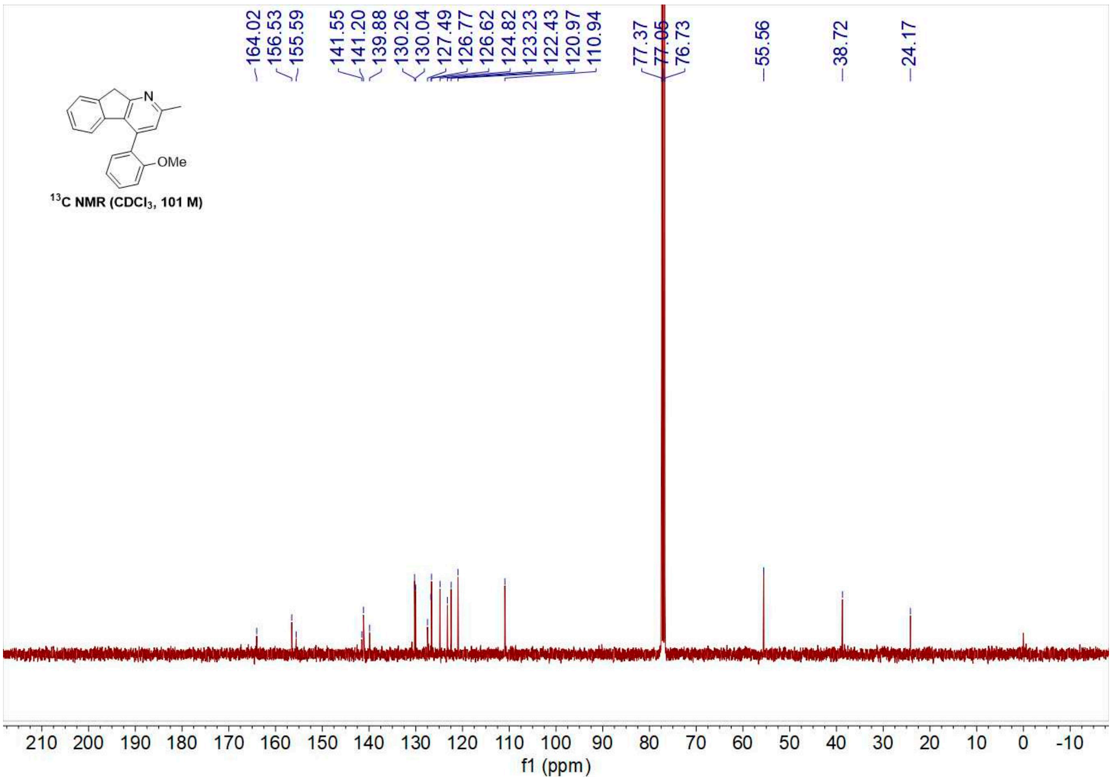

## $^{1}$H NMR (400 MHz, Chloroform-*d*) of compound 3p

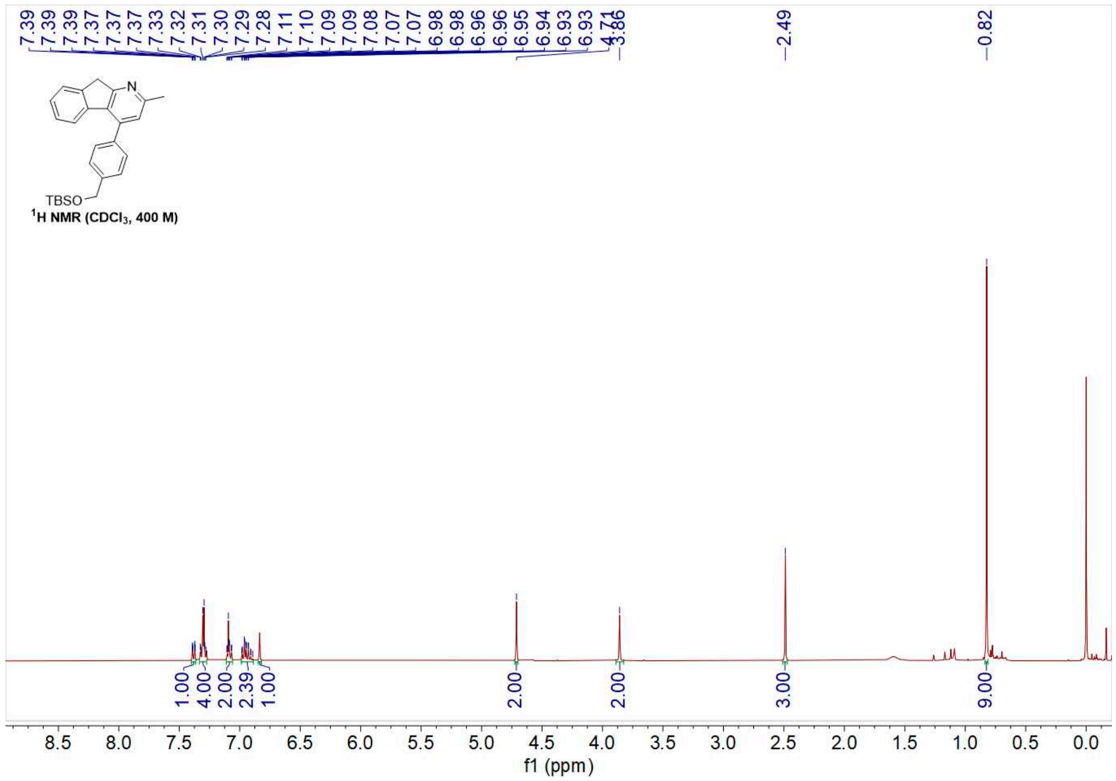

## $^{13}$C NMR (101 MHz, Chloroform-*d*) of compound 3p

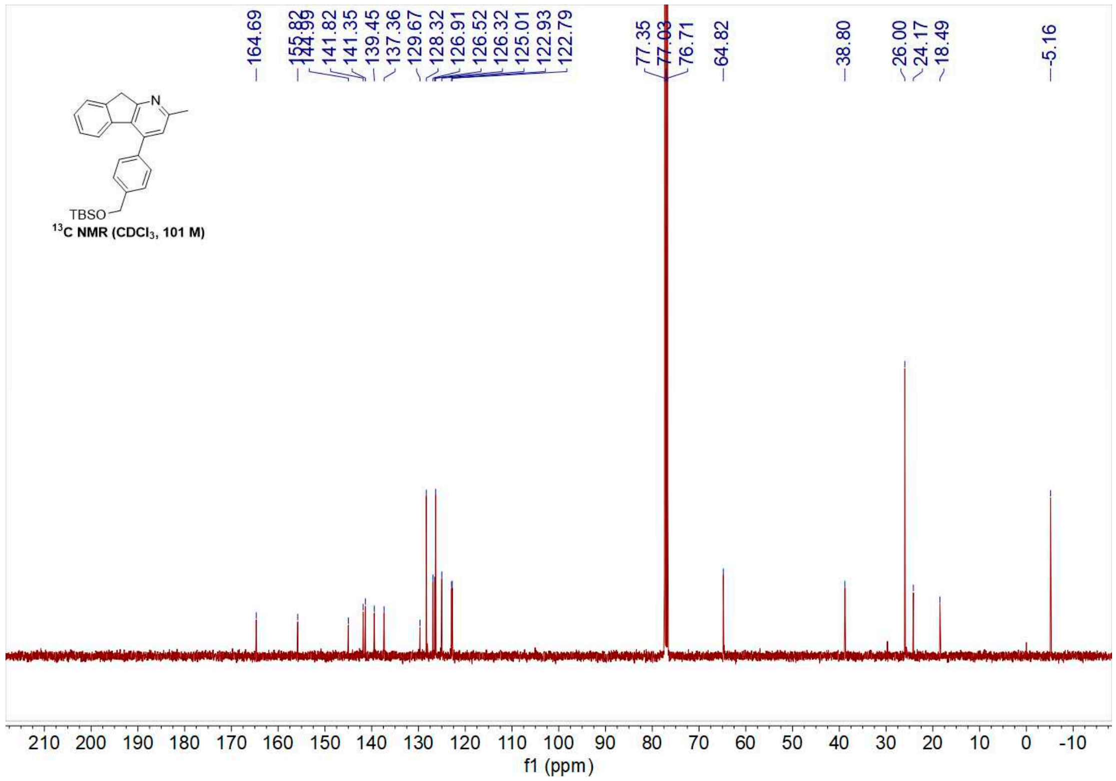

## $^{1}$H NMR (400 MHz, Chloroform-*d*) of compound 3q

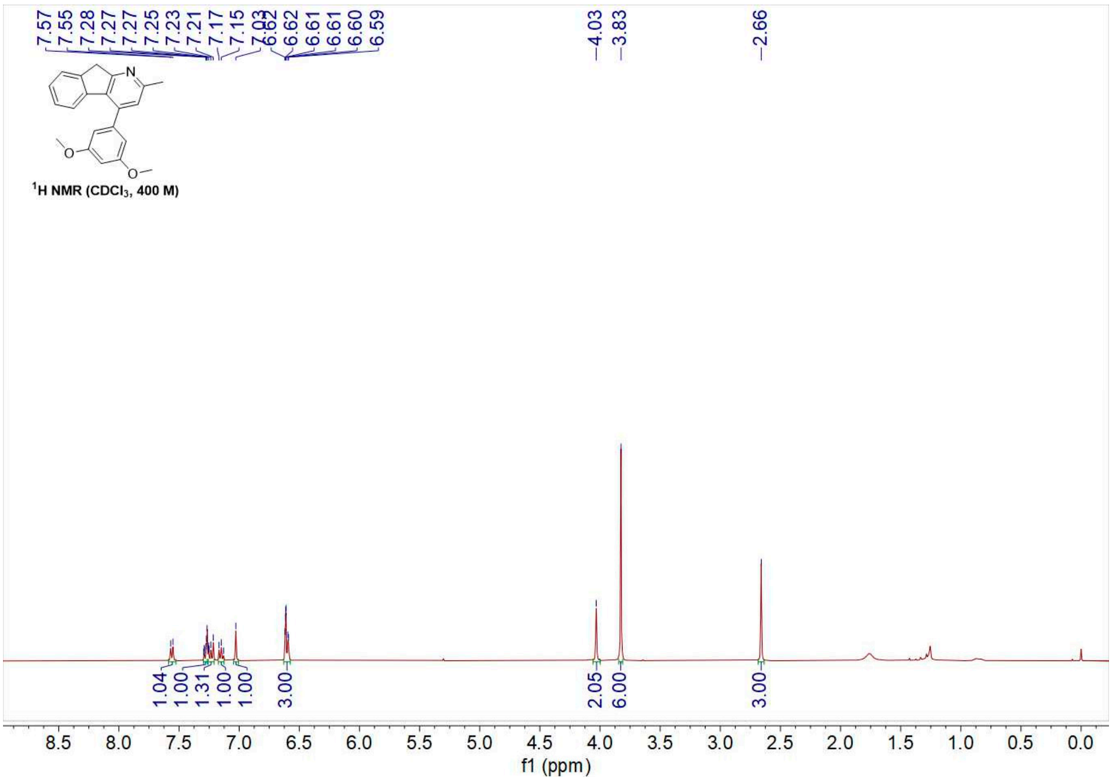


## $^{13}$C NMR (101 MHz, Chloroform-*d*) of compound 3q

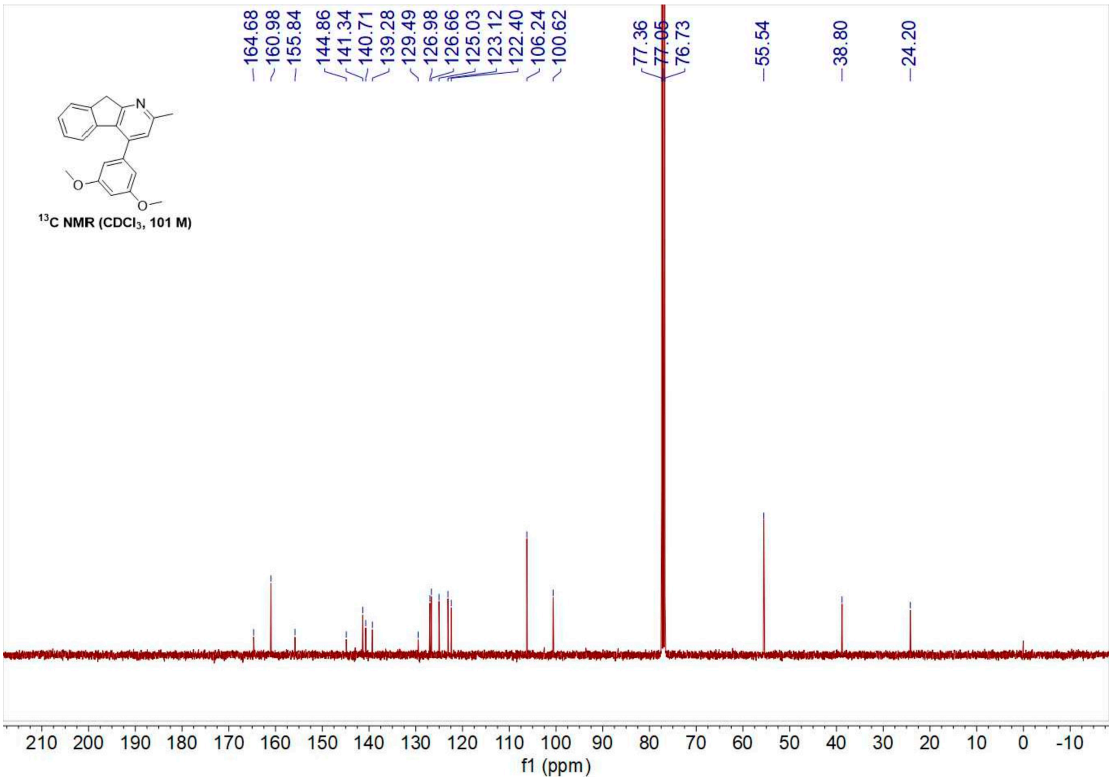

## $^{1}$H NMR (400 MHz, Chloroform-*d*) of compound 3r

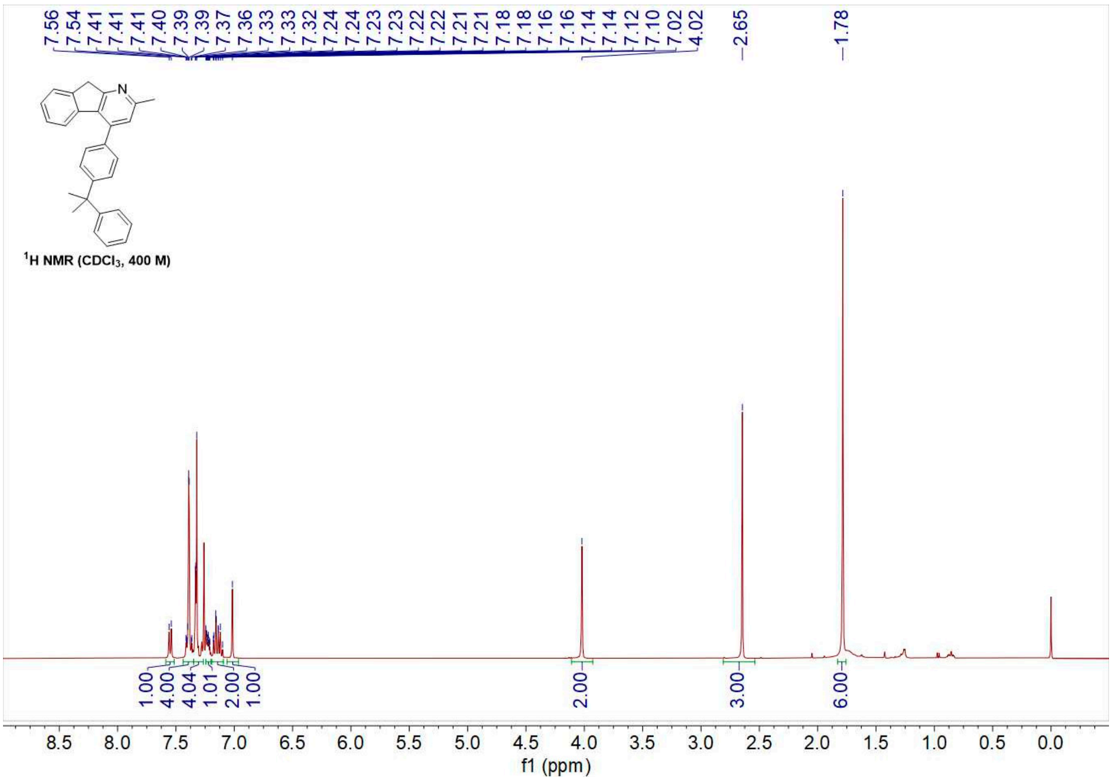


## $^{13}$C NMR (101 MHz, Chloroform-*d*) of compound 3r

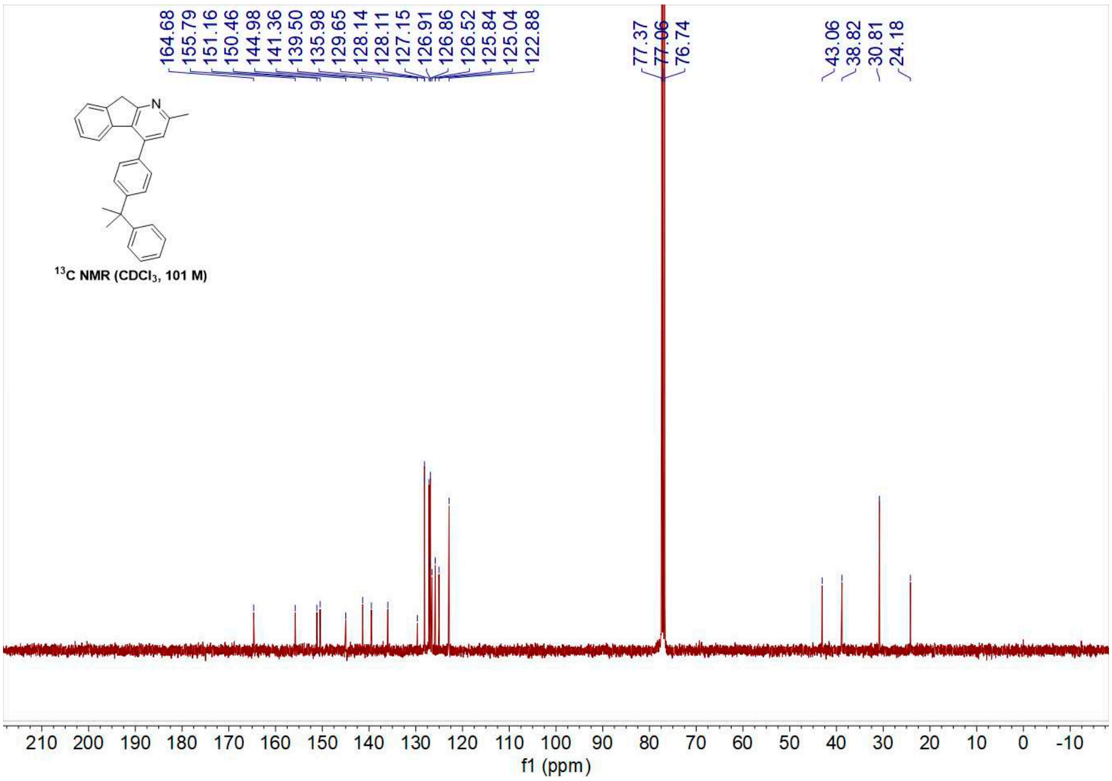

## $^{1}$H NMR (400 MHz, Chloroform-*d*) of compound 3s

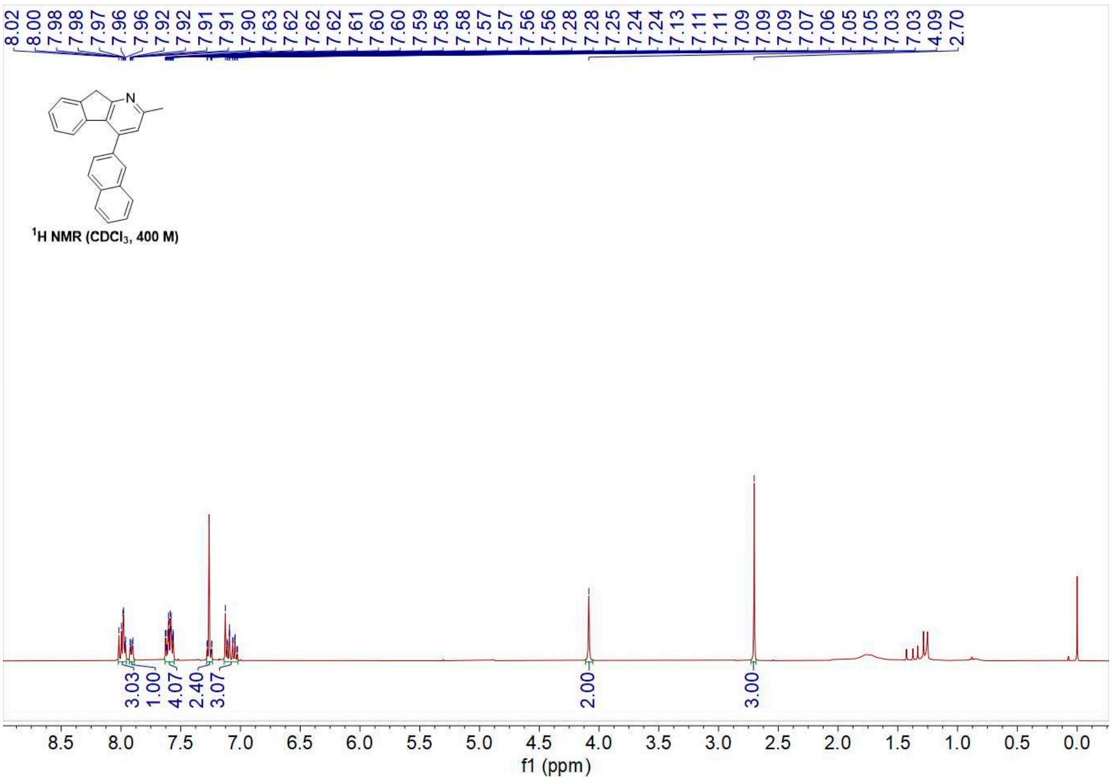


## $^{13}$C NMR (101 MHz, Chloroform-*d*) of compound 3s

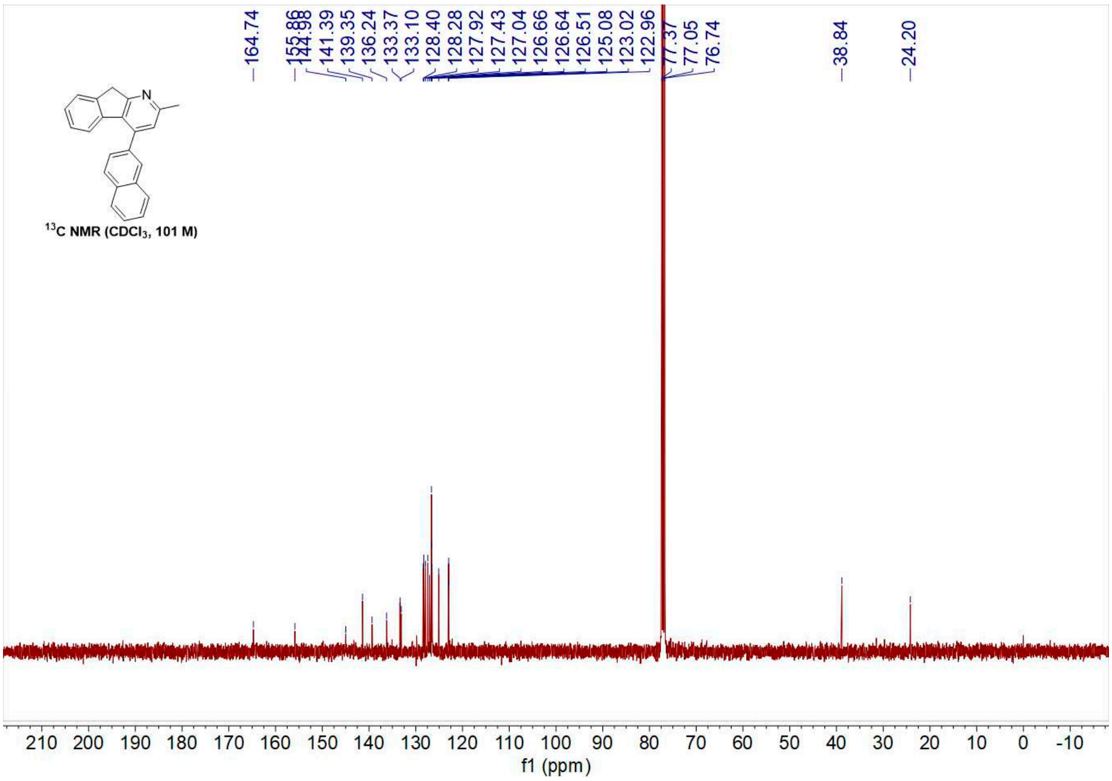

## $^{1}$H NMR (400 MHz, Chloroform-*d*) of compound 3t

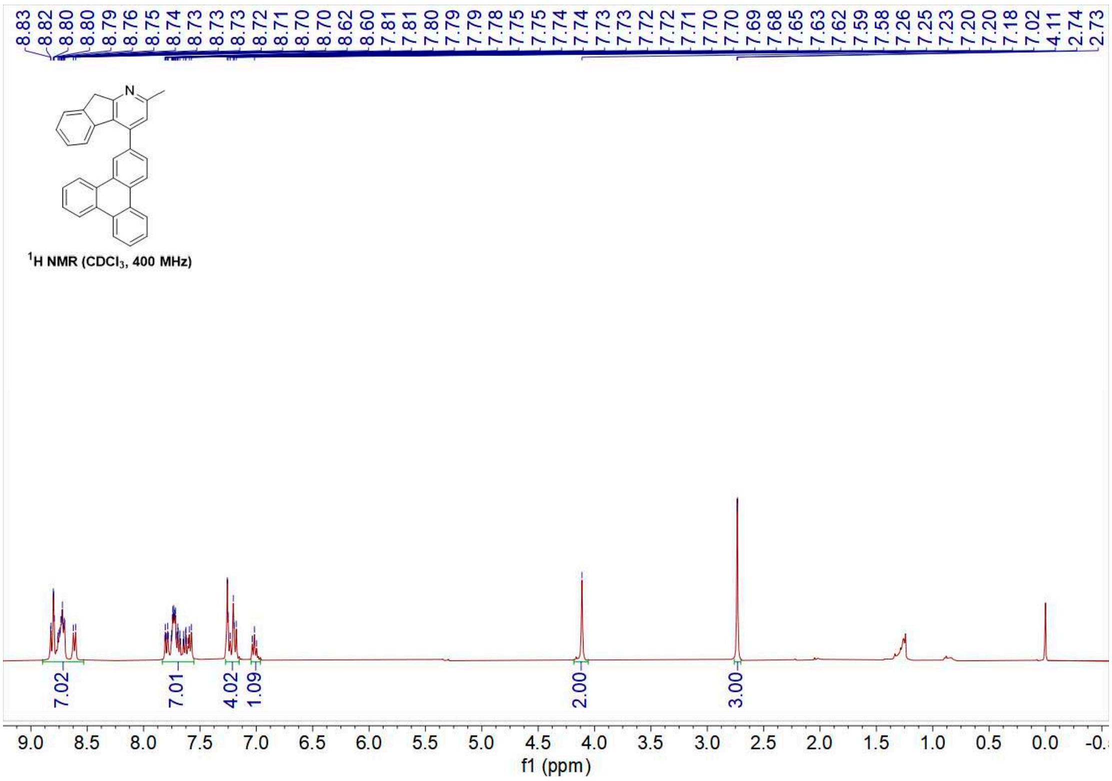


## $^{13}$C NMR (101 MHz, Chloroform-*d*) of compound 3t

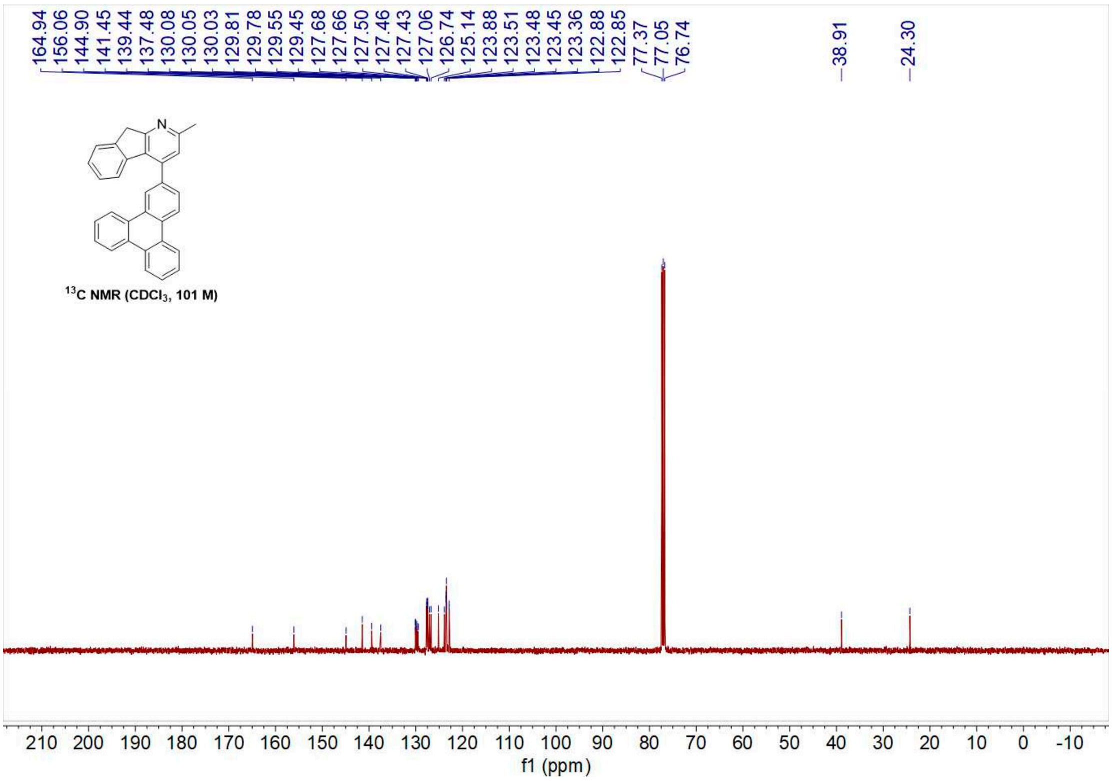

## $^1$H NMR (400 MHz, Chloroform-*d*) of compound 3u

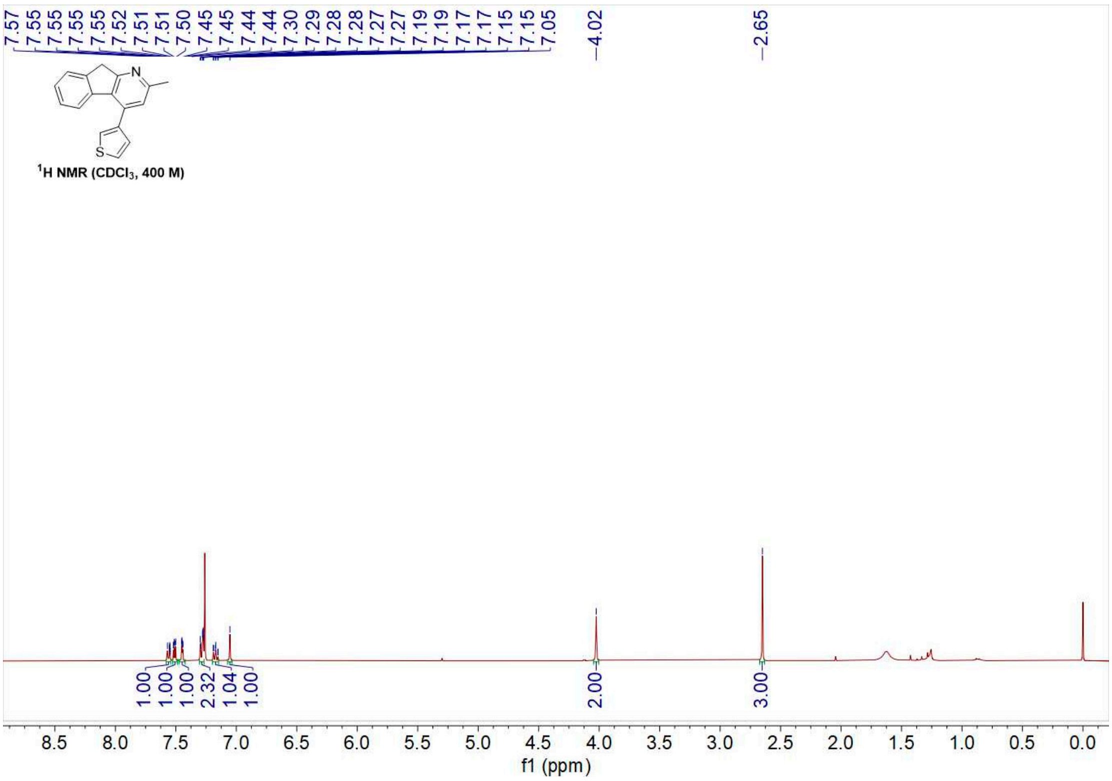

## $^{13}$C NMR (101 MHz, Chloroform-*d*) of compound 3u

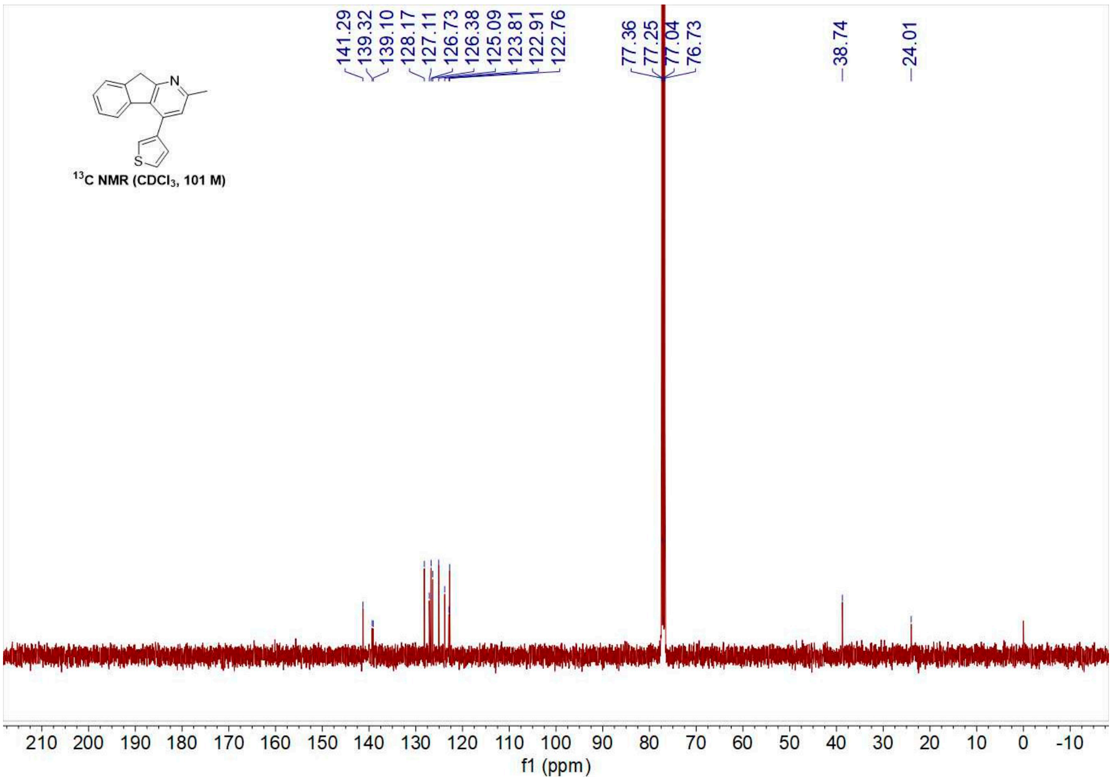

## $^{1}$H NMR (400 MHz, Chloroform-*d*) of compound 3v

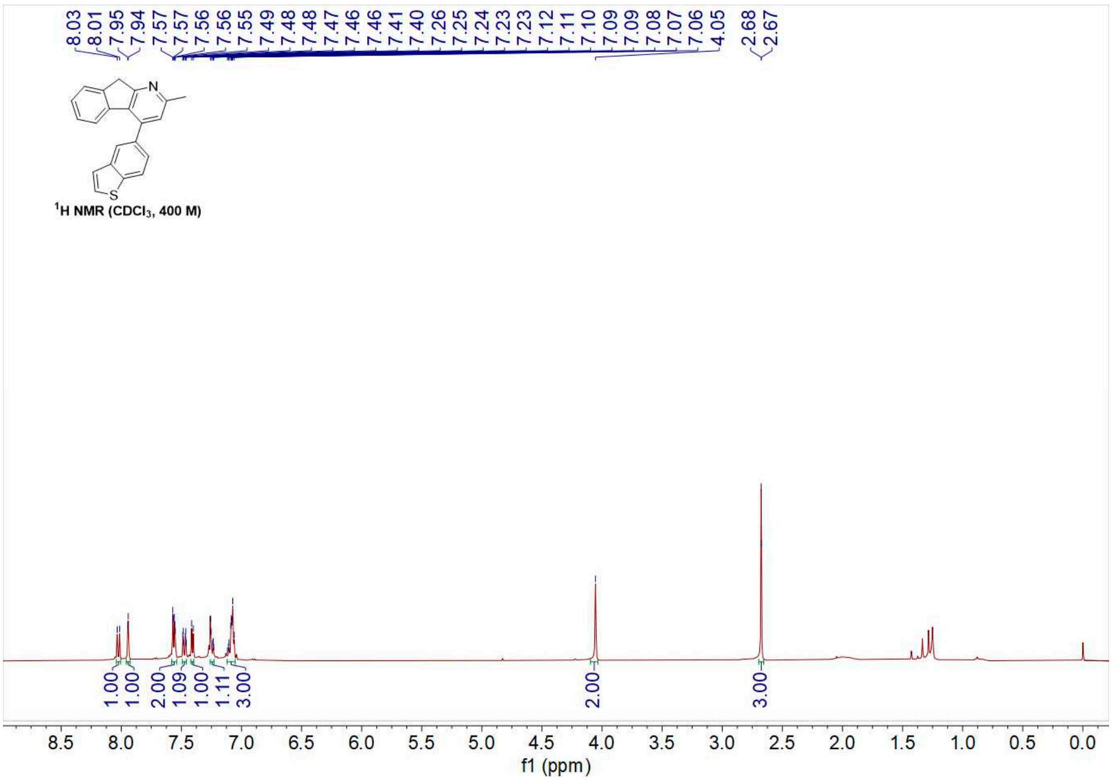

## $^{13}$C NMR (101 MHz, Chloroform-*d*) of compound 3v

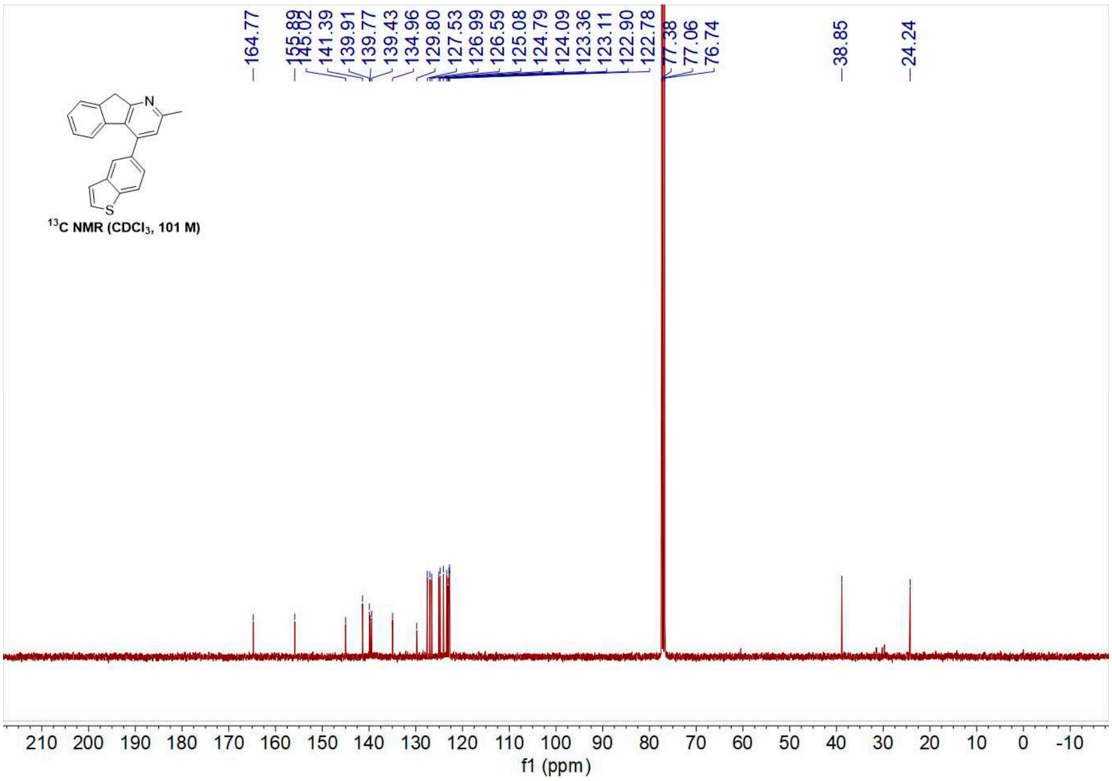

## $^{1}$H NMR (400 MHz, Chloroform-*d*) of compound 3w

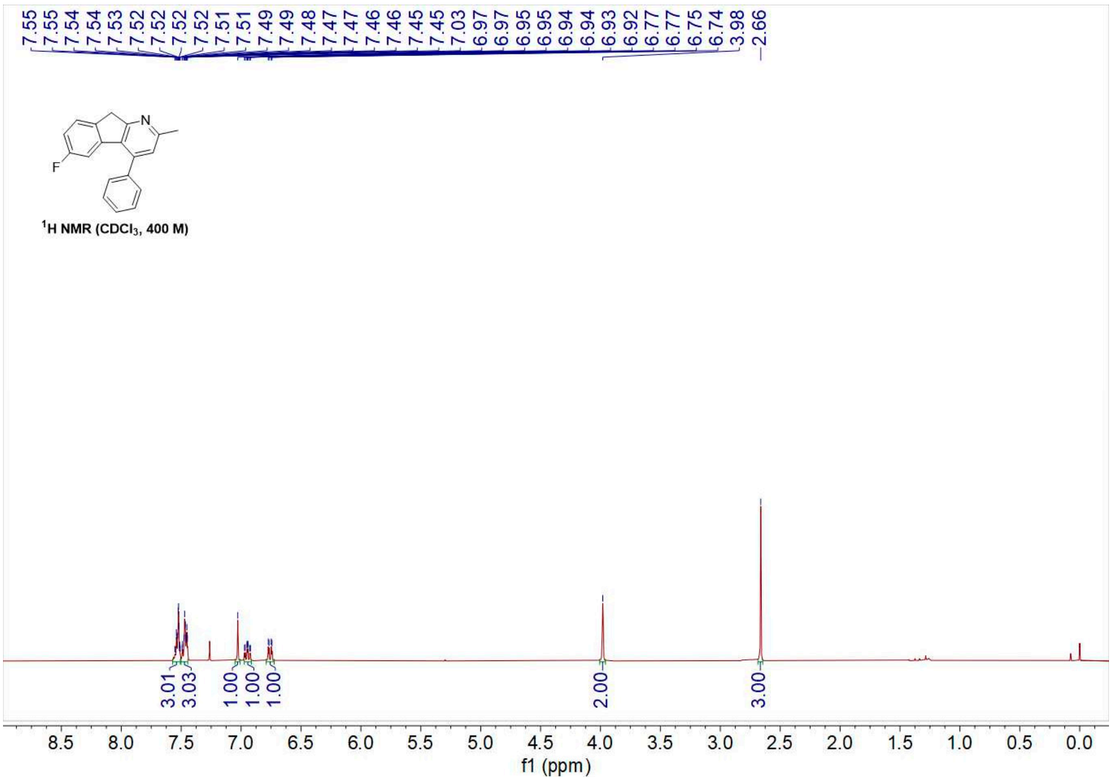


## $^{13}$C NMR (101 MHz, Chloroform-*d*) of compound 3w

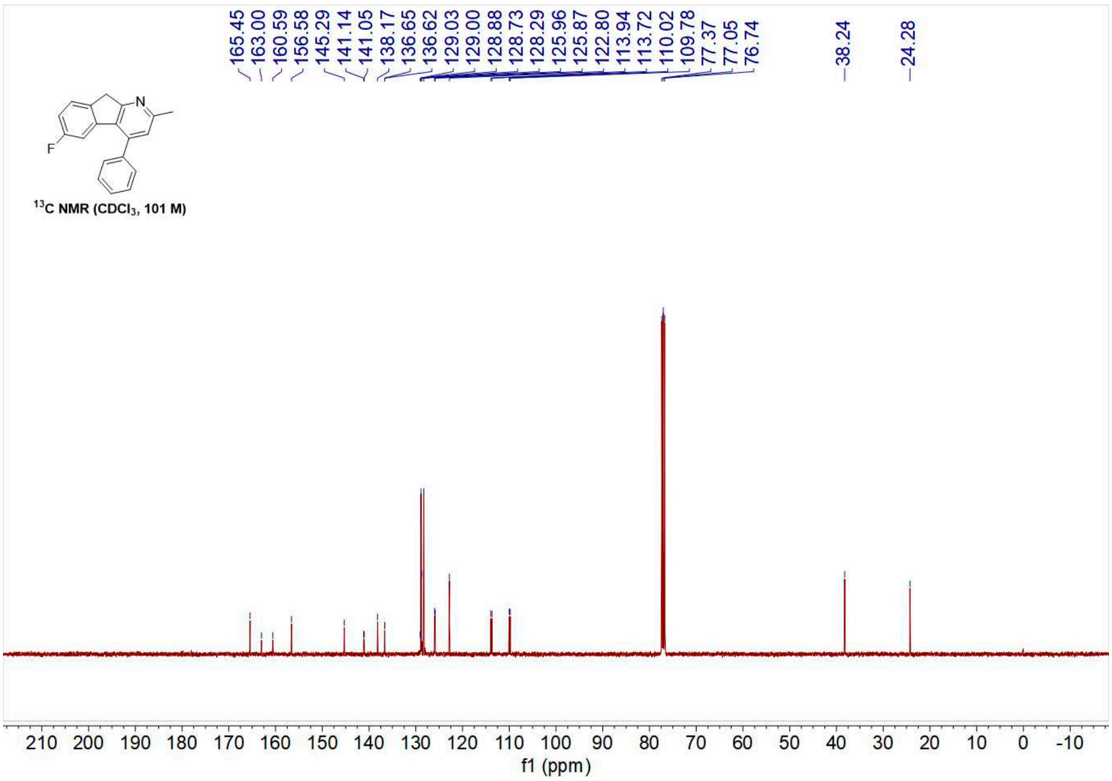

## $^{19}$F NMR (377 MHz, Chloroform-*d*) of compound 3w

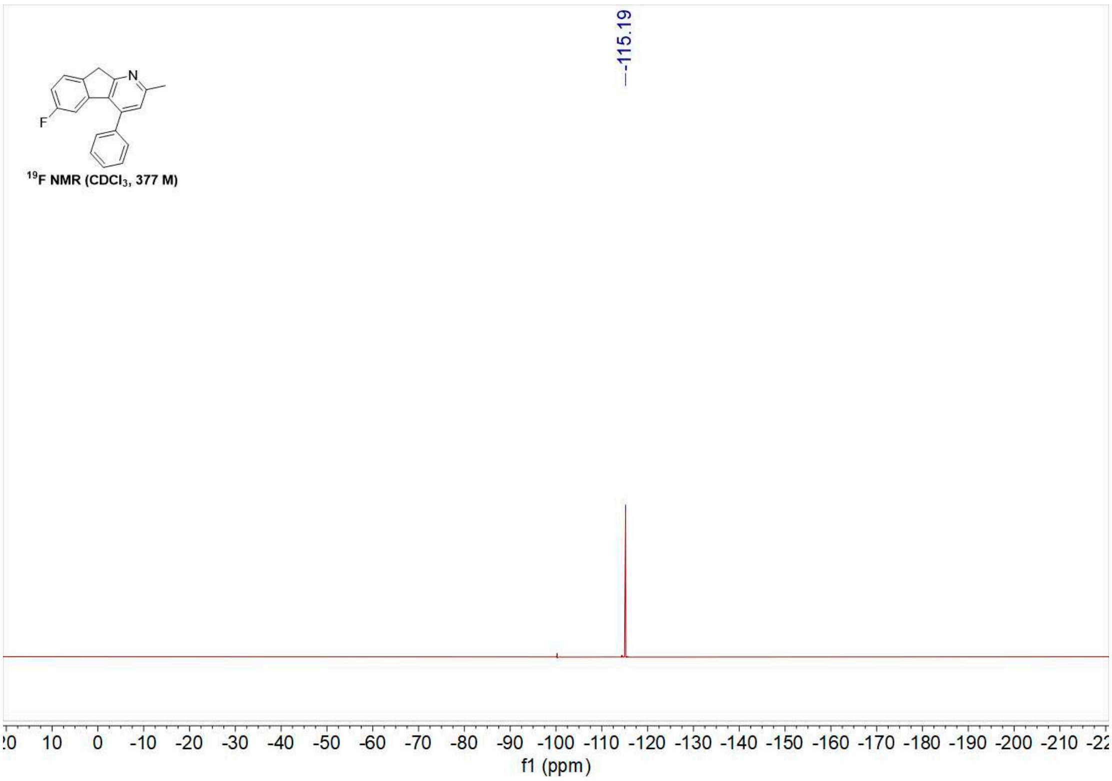


## $^{1}$H NMR (400 MHz, Chloroform-*d*) of compound 3x

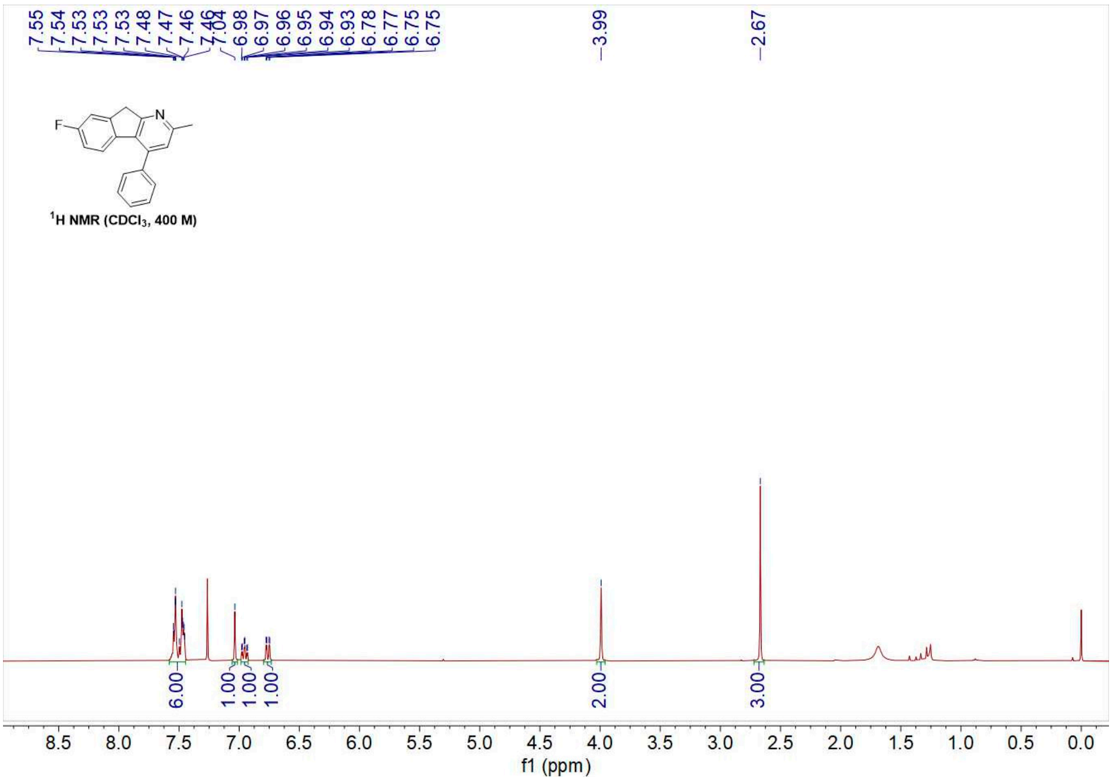

**$^{13}$C NMR (101 MHz, Chloroform-*d*) of compound 3x**

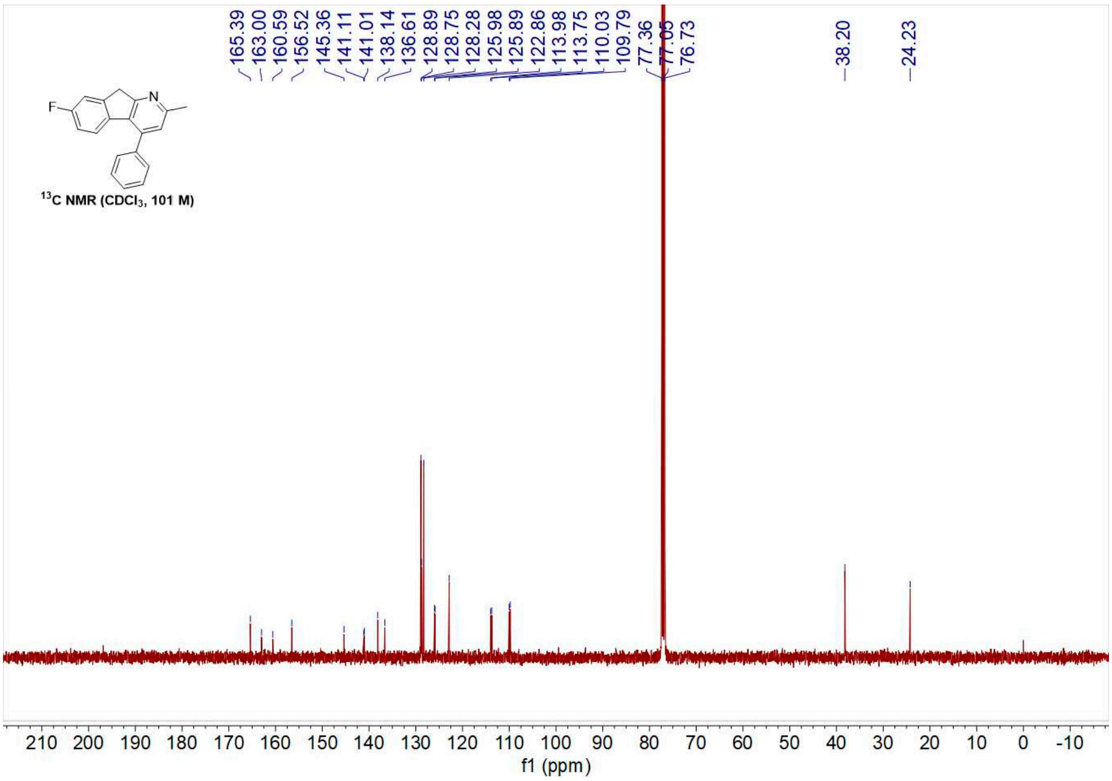


**$^{19}$F NMR (377 MHz, Chloroform-*d*) of compound 3x**

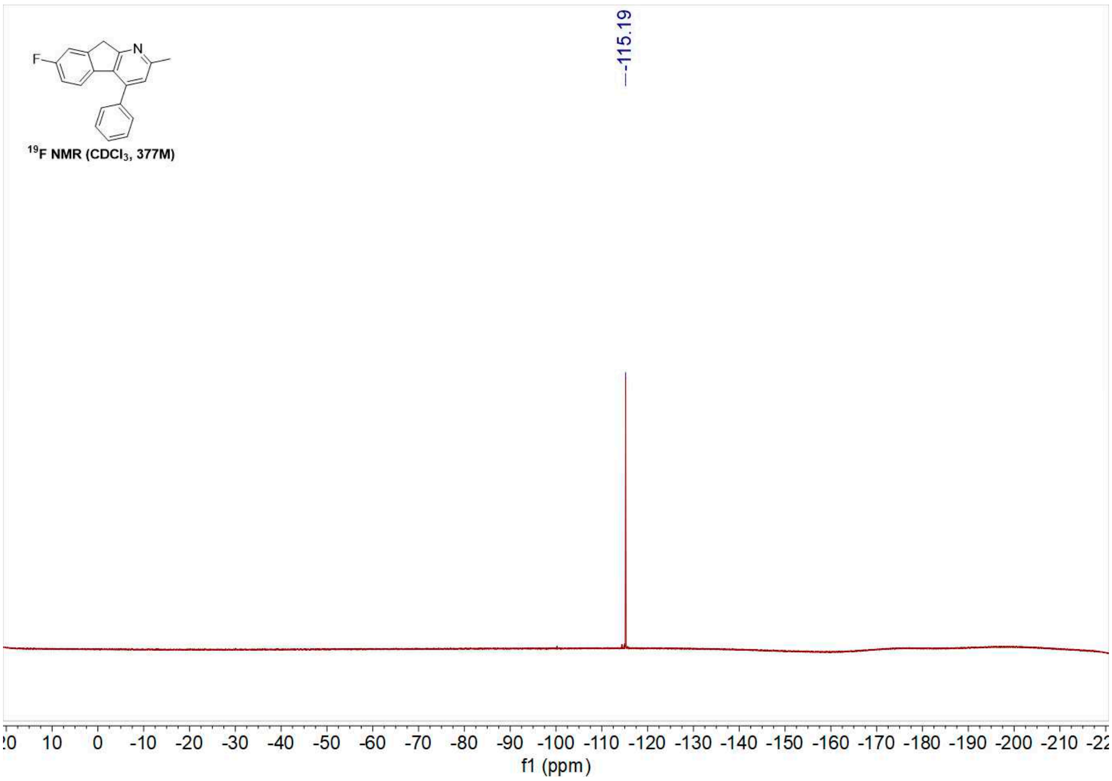

## $^{1}$H NMR (400 MHz, Chloroform-*d*) of compound 3y

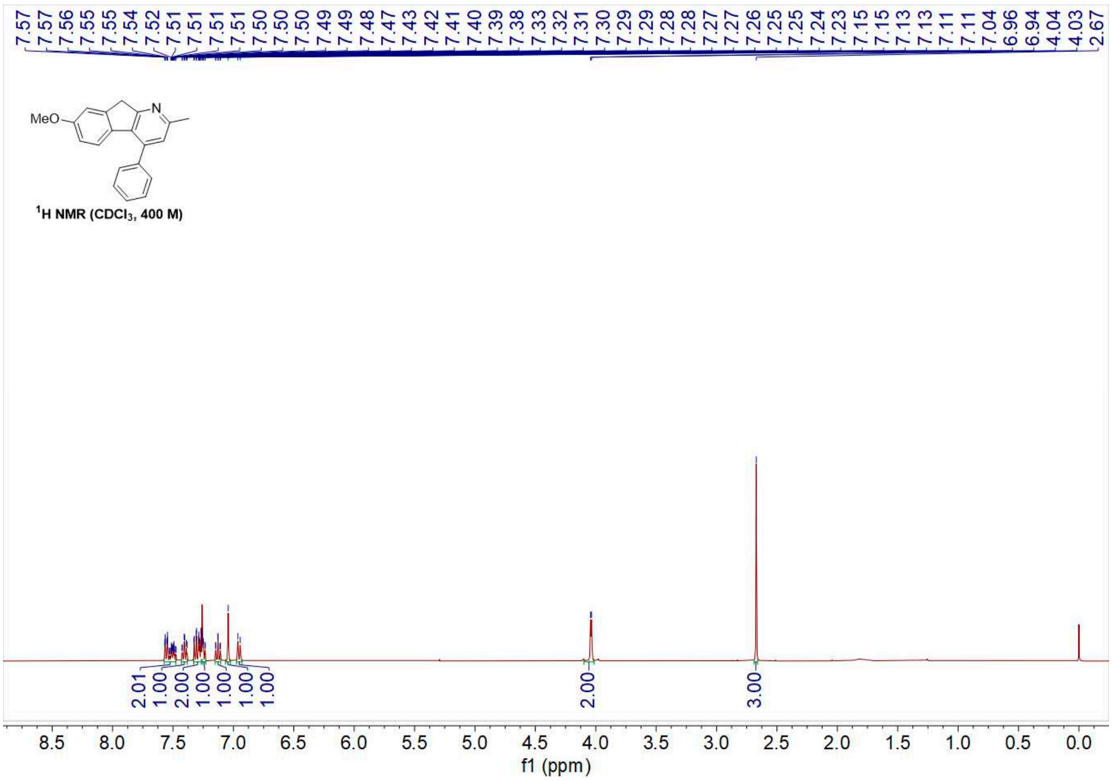


## $^{13}$C NMR (101 MHz, Chloroform-*d*) of compound 3y

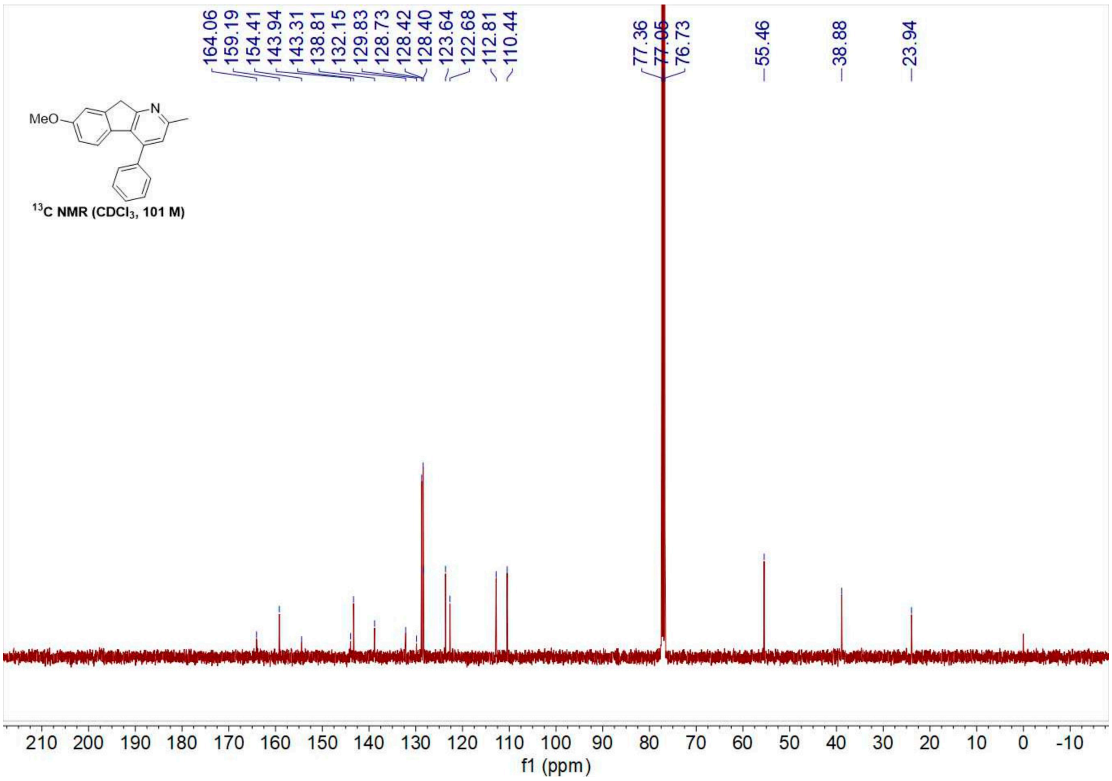

## $^1$H NMR (400 MHz, Chloroform-*d*) of compound 4a

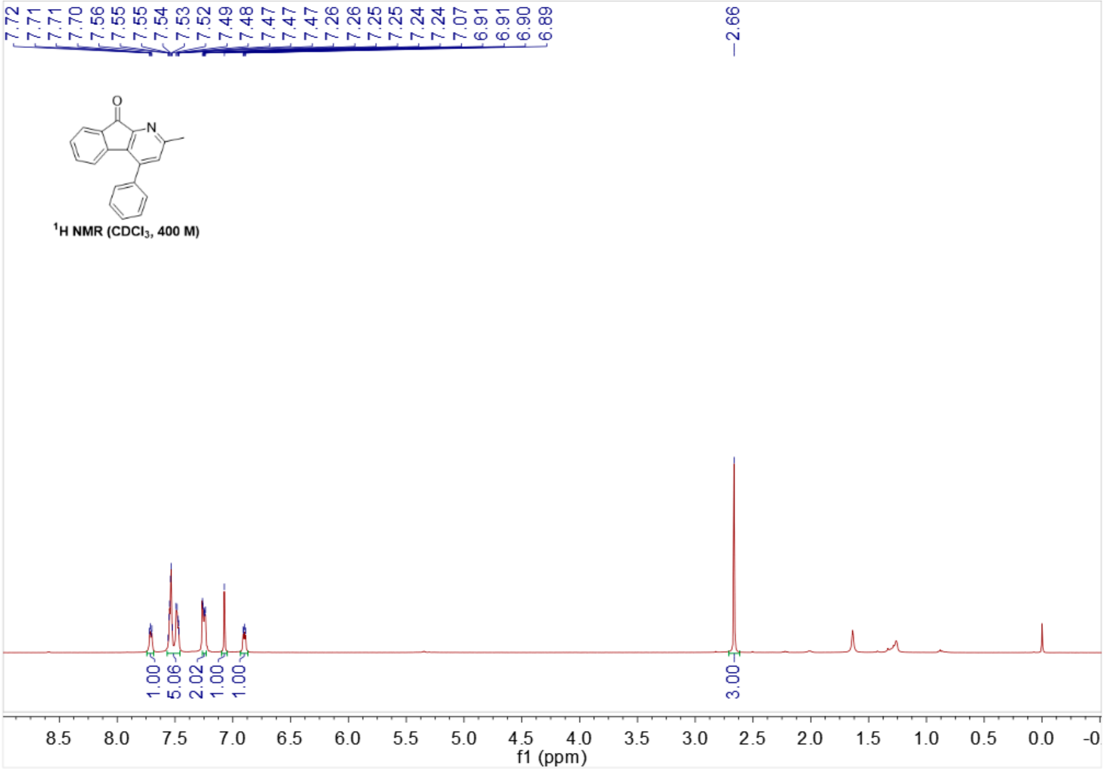


## $^{13}$C NMR (101 MHz, Chloroform-*d*) of compound 4a

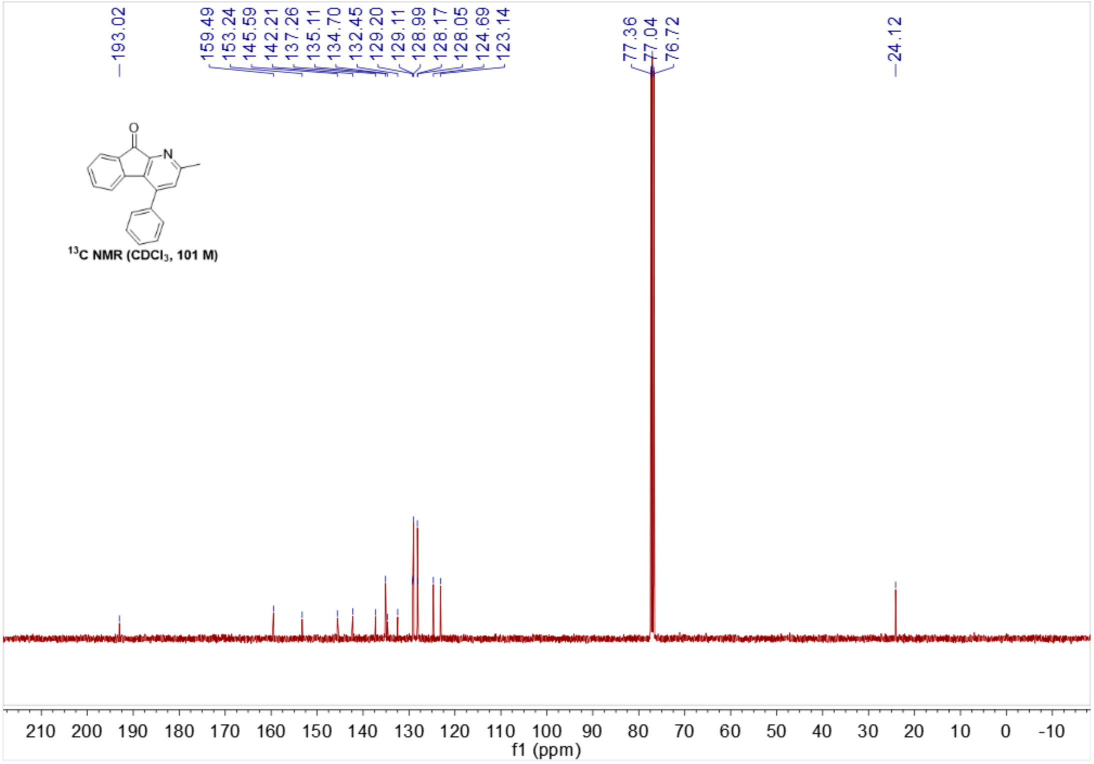

### $^{1}H$ NMR (400 MHz, Chloroform-*d*) of compound 4i

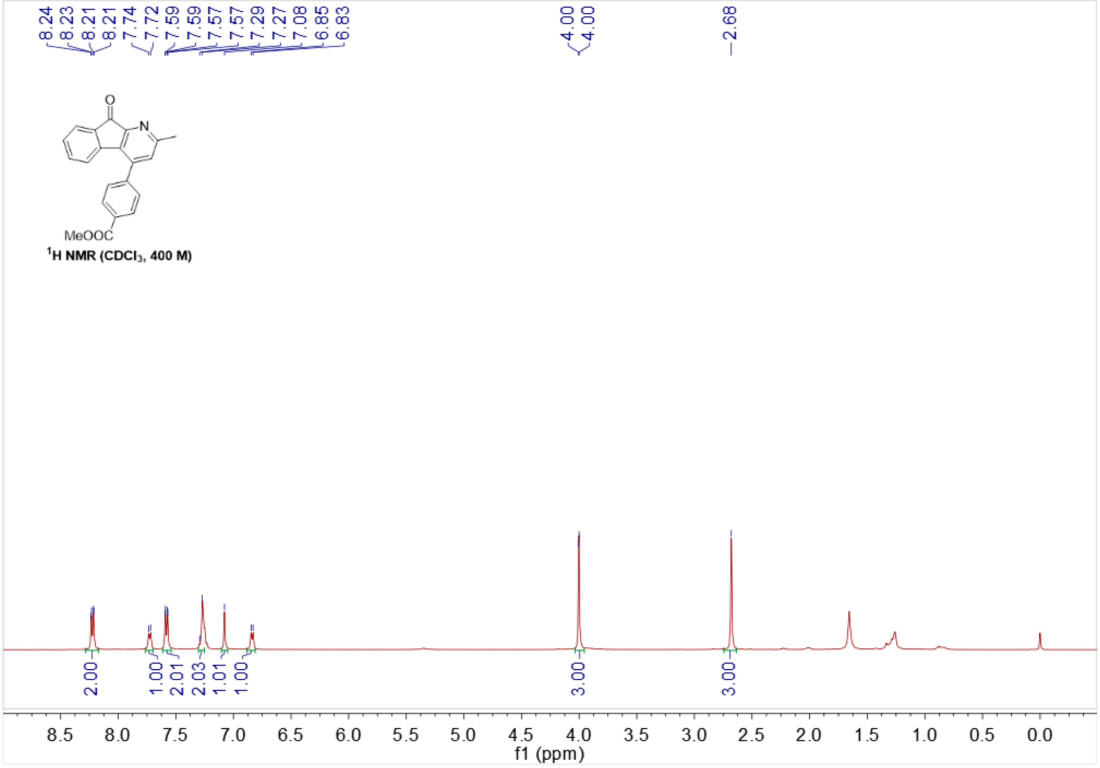


### $^{13}C$ NMR (101 MHz, Chloroform-*d*) of compound 4i

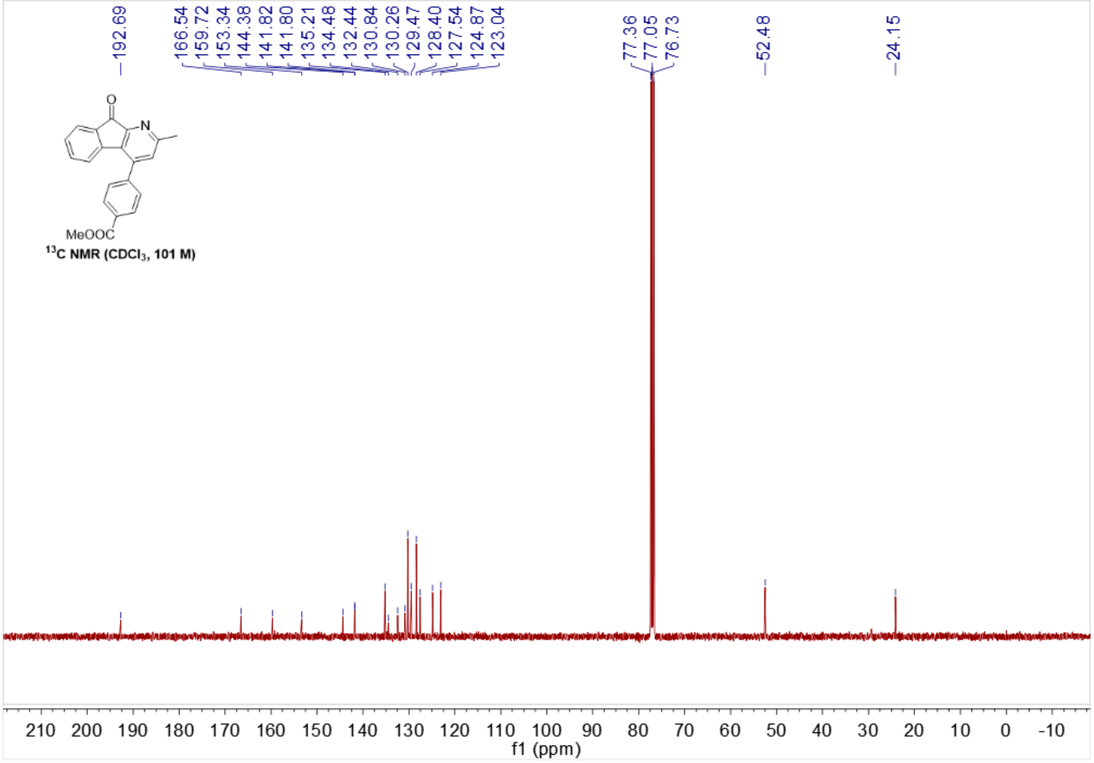

Supplement: Supplementary file 1 [file molecules-30-03629-s001.zip › molecules-3819661-supplementary.pdf]
